# Genome sequence of the Japanese oak silk moth, *Antheraea yamamai*: the first draft genome in the family Saturniidae

**DOI:** 10.1093/gigascience/gix113

**Published:** 2017-11-27

**Authors:** Seong-Ryul Kim, Woori Kwak, Hyaekang Kim, Kelsey Caetano-Anolles, Kee-Young Kim, Su-Bae Kim, Kwang-Ho Choi, Seong-Wan Kim, Jae-Sam Hwang, Minjee Kim, Iksoo Kim, Tae-Won Goo, Seung-Won Park

**Affiliations:** 1 Department of Agricultural Biology, National Academy of Agricultural Science, Rural Development Administration, 166, Nongsaengmyeong-ro, Iseo-myeon, Wanju_Gun, Jeollabuk-do, 55365, Republic of Korea; 2 C&K Genomics, Main Bldg. #420, SNU Research Park, Gwanak-ro 1, Gwanak gu, Seoul, 08826, Republic of Korea; 3 Department of Agricultural Biotechnology and Research Institute of Agriculture and Life Sciences, Seoul National University, Gwanak-ro 1, Gwanak gu, Seoul, 08826, Republic of Korea; 4 College of Agriculture and Life Sciences, Chonnam National University, Yongbong-ro 77, Buk-gu, Gwangju, 61186, Republic of Korea; 5 Department of Biochemistry, Dongguk University College of Medicine, Gyeongju-si, Gyeongsangbuk-do, 38066, Republic of Korea; 6 Department of Biotechnology, Catholic University of Daegu, Hayang-ro 13-13, Hayang-eup, Gyeongsan-si, Gyeongsangbuk-do, 38430, Republic of Korea

**Keywords:** *Antheraea yamamai*, genome assembly, Japanese silk moth, Japanese oak silk moth, wild silkworm

## Abstract

**Background:**

*Antheraea yamamai*, also known as the Japanese oak silk moth, is a wild species of silk moth. Silk produced by *A. yamamai*, referred to as tensan silk, shows different characteristics such as thickness, compressive elasticity, and chemical resistance compared with common silk produced from the domesticated silkworm, *Bombyx mori*. Its unique characteristics have led to its use in many research fields including biotechnology and medical science, and the scientific as well as economic importance of the wild silk moth continues to gradually increase. However, no genomic information for the wild silk moth, including *A. yamamai*, is currently available.

**Findings:**

In order to construct the *A. yamamai* genome, a total of 147G base pairs using Illumina and Pacbio sequencing platforms were generated, providing 210-fold coverage based on the 700-Mb estimated genome size of *A. yamamai*. The assembled genome of *A. yamamai* was 656 Mb (>2 kb) with 3675 scaffolds, and the N50 length of assembly was 739 Kb with a 34.07% GC ratio. Identified repeat elements covered 37.33% of the total genome, and the completeness of the constructed genome assembly was estimated to be 96.7% by Benchmarking Universal Single-Copy Orthologs v2 analysis. A total of 15 481 genes were identified using Evidence Modeler based on the gene prediction results obtained from 3 different methods (*ab initio*, RNA-seq-based, known-gene-based) and manual curation.

**Conclusions:**

Here we present the genome sequence of *A. yamamai*, the first genome sequence of the wild silk moth. These results provide valuable genomic information, which will help enrich our understanding of the molecular mechanisms relating to not only specific phenotypes such as wild silk itself but also the genomic evolution of Saturniidae.

## Data Description

**Figure 1:**
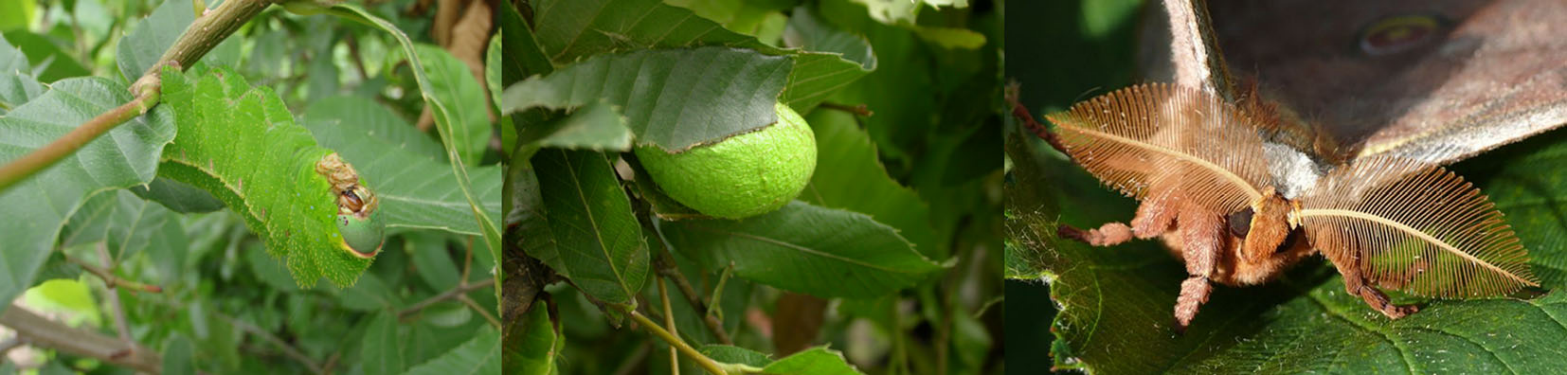
Photograph of *Antheraea Yamamai*. From left: larva, cocoon, and adult *A. yamamai*, respectively. Green color is one of the representative characteristics of tensan silk.

*Antheraea yamamai* (NCBI Taxonomy ID: 7121), also known as the Japanese oak silk moth, is a wild silk moth species belonging to the Saturniidae family (Fig. 1). Silk moths can be categorized into 2 families—Bombycidae and Saturniidae. Saterniidae has been estimated to contain approximately 1861 species, with 162 genera [1], and is known as the largest family in the Lepidoptera. Among the many species in family Saturniidae, only a few species, including *A. yamamai*, can be utilized for silk production. Previous phylogenetic studies have shown that the family Saturniidae shares common ancestors with the family Sphingidae, including the hawk moth (*Macroglossum stellatarum*), and the Bombycidae family, including the most representative silkworm, *Bombyx mori* [2]. The estimated divergence time between *A. yamamai* and *B. mori* is 84 million years ago (MYA), making it similar to the 88 MYA estimated divergence time between the human and mouse [3, 4].

*A. yamamai* produces a specific silk called tensan silk [5], which shows distinctive characteristics compared with common silk from *B. mori*, including characteristics such as thickness, bulkiness, compressive elasticity, and resistance to dyeing chemicals [6–8]. These characteristics have received the attention of researchers as a new biomaterial for use in various fields [9–11]. Additionally, tensan silk also has been studied for its applications to human health [12–15]. However, despite the potential importance of the wild silk moth in research and economic fields, no whole genomic information is currently available for this or any other species from the family Saturniidae.

In this study, we present the annotated genome sequence of *A. yamamai*, the first published genome in the family Saturniidae, with transcriptome datasets collected from 10 different body organ tissues. These data will be a fundamental resource for future studies and provide more insight into the genome evolution and molecular phylogeny of the family Saturniidae.

### Sample preparation and sequencing

For whole-genome sequencing, we selected 1 male sample (Ay-7-male1) from a breeding line (Ay-7) of *A. yamamai* raised at the National Academy of Agricultural Science, Rural Development Administration, Korea. In lepidopterans, males are homogametic (ZZ), and selecting a male sample can reduce the complexity of assembly from excessive repeats on the W chromosome in females. For genomic library construction, we removed the guts of *A. yamamai* to prevent contamination of genomes from other organisms such as gut microbes and oak, the main food source of *A. yamamai*. Details of the sample preparation process used in this study are presented in the Supplementary Data. Genomic DNA was extracted using a DNeasy Animal Mini Kit (Qiagen, Hilden, Germany), and the quality of extracted DNA was checked using trenean, picogreen assay, and gel electrophoresis (1% agarose gel/40 ng loading). After quality control processing, we were left with a total of 61.5 ug of *A. yamamai* DNA for genome sequencing. Using standard Illumina whole-genome shotgun (WGS) sequencing protocol (paired-end and mate-pair), we added 2 long read sequencing platforms, Moleculo (Illumina synthetic long read) and RS II (Pacific Bioscience). Tables 1–3 show a summary of generated data for each library used in this study. RNA-seq libraries were also constructed for 10 different tissues (hemocyte, malpighian tube, midgut, fat body, anterior-middle/silk gland, posterior/silk gland, head, integument, testis, ovary) with 3 biological replicates following standard manufacturer protocol (Illumina, San Diego, CA, USA). For this, more than 100 individual *A. yamamai* samples in 5 instar stages from the same breeding line were used for tissue anatomy, and 3 samples from each tissue were selected based on the quality of extracted RNA. Details of transcriptome library construction are shown in the Supplementary Data. Information of libraries and generated data is provided in Table 4, and a total of 147 Gb of genomic data and 76 Gb of transcriptomic data was generated for this study.

**Table 1:** Summary statistics of generated whole-genome shotgun sequencing data using Illumina Nextseq 500

Library name	Library type	Insert size	Platform	Read length	No. of reads	Total base, bp	Reads retained after trimming
350 bp	Paired-end	350 bp	Nextseq500	151	293 176 268	44 269 616 468	291 070 362
700 bp	Paired-end	700 bp	Nextseq500	151	246 945 900	37 288 830 900	244 698 580
3 Kbp	Mate-pair	3 Kbp	Nextseq500	76	284 204 762	21 599 561 912	195 095 164
6 Kbp	Mate-pair	6 Kbp	Nextseq500	76	246 238 370	18 714 116 120	152 496 372
9 Kbp	Mate-pair	9 Kbp	Nextseq500	76	239 919 538	18 233 884 888	148 612 724
Total					1 310 484 838	140 106 010 288	1 031 973 202

**Table 2:** Summary statistics of generated Illumina synthetic long read (Moleculo) library

	500–1499 bp	≥1500 bp
No. of assembled reads	302 132	342 738
No. of bases in assembled read	268 853 717	1 205 349 082
N50 length of assembled read	960	4031

**Table 3:** Summary statistics of generated long reads data using Pacbio RS II system

No. of reads	1005,571
Total bases	5836 969 225
Length of longest (shortest) read	50 132 (50)
Average read length	5804.63

**Table 4:** Summary statistics of generated transcriptome data obtained from 6 organ tissues using Illumina platform

Tissue	Sample name	Read length	Read count	Total base, bp
Hemocyte	Hemocyte_1	76	20 815 674	1 581 991 224
	Hemocyte_2	76	26 704 666	2 029 554 616
	Hemocyte_2	76	53 068 562	4 033 210 712
Malpighian tube	Malpighi_1	76	22 635 428	1 720 292 528
	Malpighi_2	76	24 893 788	1 891 927 888
	Malpighi_3	76	45 213 164	3 436 200 464
Midgut	Midgut_1	76	23 350 138	1 774 610 488
	Midgut_2	76	24 597 972	1 869 445 872
	Midgut_3	76	50 949 986	3 872 198 936
Head	Head_1	76	26 526 276	2 015 996 976
	Head_2	76	26 581 124	2 020 165 424
	Head_3	76	40 900 456	3 108 434 656
Integument	Skin_1	76	24 592 846	1 869 056 296
	Skin_2	76	42 775 430	3 250 932 680
	Skin_3	76	35 043 570	2 663 311 320
Fat body	Fat Body_1	76	24 637 810	1 872 473 560
	Fat Body_2	76	24 037 494	1 826 849 544
	Fat Body_3	76	40 817 582	3 102 136 232
Anterior-middle/silk gland	AM/Silk Gland_1	76	21 399 638	1 626 372 488
	AM/Silk Gland_2	76	24 292 386	1 846 221 336
	AM/Silk Gland_3	76	37 331 530	2 837 196 280
Posterior/silk gland	P/Silk Gland_1	76	27 359 580	2 079 328 080
	P/Silk Gland_2	76	23 300 962	1 770 873 112
	P/Silk Gland_3	76	39 421 430	2 996 028 680
Testis	Testis_1	76	40 890 404	3 107 670 704
	Testis_2	76	45 733 846	3 475 772 296
	Testis_3	76	44 985 224	3 418 877 024
Ovary	Ovary_1	76	40 797 628	3 100 619 728
	Ovary_2	76	40 409 752	3 071 141 152
	Ovary_3	76	42 417 892	3 223 759 792

### Genome assembly and evaluation

**Figure 2:**
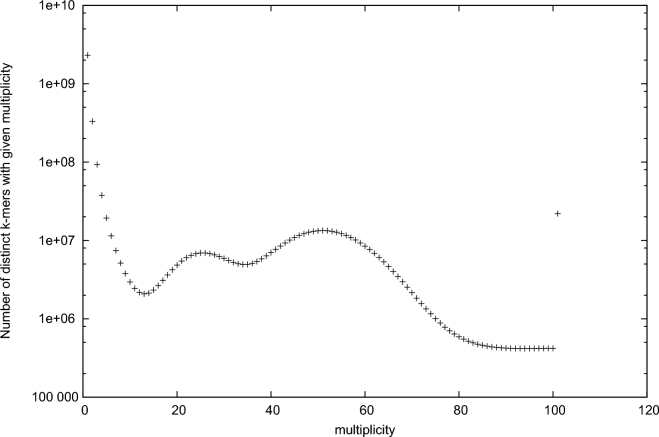
19-mer distribution of *A. yamamai* genome using jellyfish with 350-bp paired-end whole genome sequencing data.

Before conducting genome assembly, we conducted k-mer distribution analysis using a 350-bp paired-end library in order to estimate the size and characteristics of the *A. yamamai* genome. The quality of our generated raw data was checked using FASTQC (FastQC, RRID:SCR_014583) [16]. Sequencing artifacts such as adapter sequences and low-quality bases were removed using Trimmomatic (Trimmomatic, RRID:SCR_011848) [17]. Jellyfish [18] was used to count the k-mer frequency for estimation of the genome size of *A. yamamai*. Figure 2 shows the 19-mer distribution of the *A. yamamai* genome using a 350-bp paired-end library. In the 19-mer distribution, the second peak, at approximately half the coverage value (x-axis) of the main peak, indicates heterozygosity. Although the inbred line used in this study was the single pair sib-mating maintained for more than 10 generations, high heterozygosity still remains. This phenomenon has been observed in a previous genomic study of the Diamondback moth (*Plutella xylostella*), and sustained heterozygosity as an important genomic characteristic was hypothesized to be a result of environmental adaption [19]. The underlying mechanism of the sustained heterozygosity is unclear, but associative overdominance can be one of the candidate explanations of this phenomenon [20, 21]. Based on the result of 19-mer distribution analysis, the genome size of *A. yamamai* was estimated to be 709 Mb. However, this size might be larger than the real genome size of *A. yamamai* because high heterozygosity could affect the estimation of genome size based on the K-mer distribution. Next, we conducted error correction on Illumina paired-end libraries using the error correction module of Allpaths-LG [22] before the initial contig assembly process (ALLPATHS-LG, RRID:SCR_010742). After error correction, initial contig assembly with 350-bp and 700-bp libraries was conducted using SOAP denovo2 [23] with the parameter option set at K = 19; this approach showed the best assembly statistics compared with other assemblers and parameters (SOAPdenovo2, RRID:SCR_014986). Quality control processing for mate-pair libraries and scaffolding was conducted using Nxtrim [24] and SSPACE (SSPACE, RRID:SCR_011848) [25], respectively. At each scaffolding step, SOAP Gapcloser [23] with -l 155 and -p 31 parameters was repeatedly used to close the gaps within each scaffold. In order to obtain a higher-quality genome assembly of *A. yamamai*, we employed several long read scaffolding strategies using SSPACE-LongRead [26]. First, we used an Illumina synthetic long read sequencing platform called Moleculo, which has been proven valuable for the study of highly heterozygous genomes in previous studies [27, 28]. After scaffolding was performed using SSPACE-LongRead with Illumina synthetic long read data, the total number of assembled scaffolds was effectively reduced from 398 446 to 24 558. The average scaffold length was also extended from 1.7 Kb to 24.8 Kb. However, there was no impressive improvement in N50 length (approximately 91 Kb to 112 Kb) of assembled scaffolds. Therefore, we employed another type of long read data generated from 10 cells of Pacbio RS II system with P6-C4 chemistry. After final scaffolding processing using Pacbio long reads, the number of scaffolds was reduced to 3675, and N50 length was effectively extended from 112 Kb to 739 Kb. Summary statistics of the assembled *A. yamamai* genome are provided in Table 5. Final assembly of the *A. yamamai* genome was 656 Mb (>2 kb) long with 3675 scaffolds, and the N50 length of assembly was 739 Kb with a 34.07% GC ratio. To evaluate the quality of the assembled genome, we conducted Benchmarking Universal Single-Copy Orthologs (BUSCO) analysis [29] using BUSCO v2.0 with insecta_odb9 including 1658 BUSCOs from 42 species (BUSCO, RRID:SCR_015008). From BUSCO analysis, 96.7% of BUSCOs were completely detected in the assembled genome (1576: complete and single-copy; 27: complete and duplicated) among 1658 tested BUSCOs. The numbers of fragmented and missing BUSCOs were 21 and 34, respectively. Based on the result of BUSCO analysis, the genome of *A. yamamai* presented here was considered properly constructed for downstream analysis.

**Table 5:** Summary statistics of the *A. yamamai* genome (>2 kb)

Assembled genome	
Size, 1n	656 Mb
GC level	34.07
No. of scaffolds	3675
N50 of scaffolds, bp	739 388
No. of bases in scaffolds, %	19 257 439 (2.93)
Longest (shortest) scaffolds, bp	3 156 949 (2003)
Average scaffold length, bp	178 657.53

### Repeat identification and comparative repeat analysis

**Figure 3:**
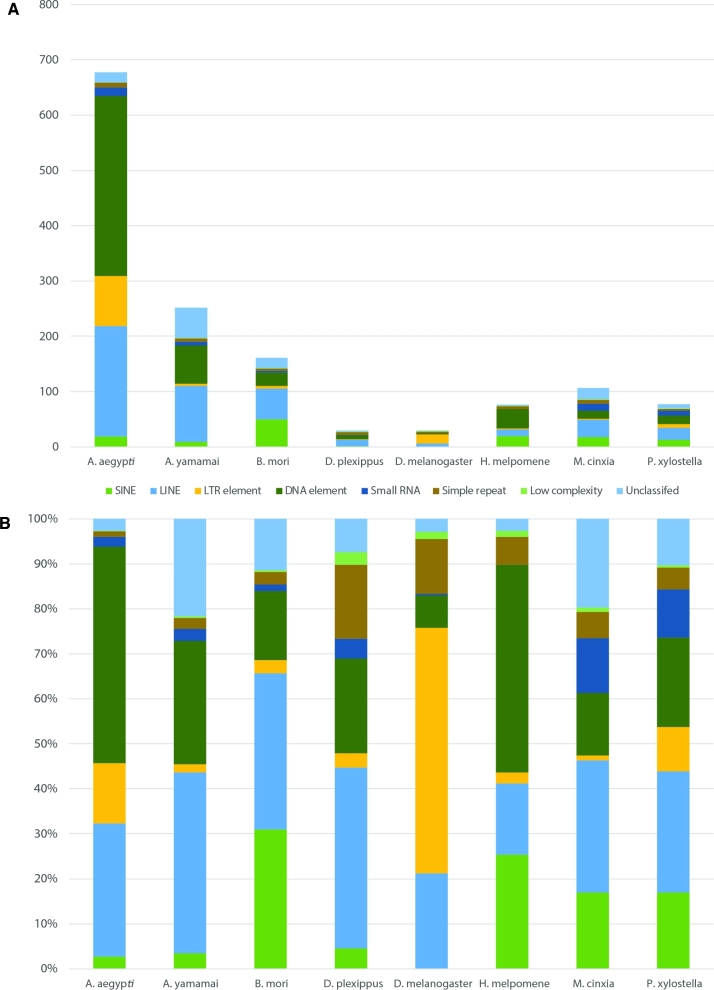
Amount and proportion of identified repeat element from 8 species including *A. yamamai.* A) Absolute amount of repeat element classified into 8 different categories. B) Proportion of each repeat element in identified total repeat element.

To identify repeat elements of the *A. yamamai* genome, a custom repeat library was constructed using RepeatModeler with RECON [30], RepeatScout [31], and TRF [32]. The resulting constructed custom repeat library for *A. yamamai* was further curated using the CENSOR [33] search, and the curated library was employed in RepeatMasker [34] with Repbase [35]. RepeatMasker (RepeatMasker, RRID:SCR_012954) was conducted with RMBlast and the “no_is” option for skipping bacterial insertion element check. Table 6 summarizes the proportion of identified mobile elements in the A. *yamamai* genome. The most prevalent repeat elements in the *A. yamamai* genome were LINE elements (101 Mb, 15.31% of total genome), and total repeat elements accounted for 37.33% of the total genome. In order to compare the repeat elements of *A. yamamai* with those of other genomes, we conducted the same process for 7 public genomes that are close neighbors of *A. yamamai*—*Aedes aegypti* [36], *Bombyx mori* [37], *Danaus plexippus* [38], *Drosophila melanogaster* [39], *Heliconius Melpomene* [40], *Melitaea cinxia* [41], and *Plutella xylostella* [19]. Figure 3 displays the amount and proportion of identified repeat elements from the 8 species. Despite the small genome size of *B. mori*, the total amount of identified SINE elements in the *B. mori* genome was 5.77 times larger than that of *A. yamamai*. The top 5 expanded repeat elements in the *A. yamamai* genome were DNA/RC, LINE/L2, LINE/RTE-BovB, DNA/TcMar-Mariner, and LINE/CR1. Among these, DNA/TcMar-Mariner was the specifically expanded repeat element in *A. yamamai* among the 8 species. In *B. mori*, SINE/tRNA-CR1, LINE/Jockey, DNA/RC, LINE/CR1-Zenon, and LINE/RTE-BovB were the top 5 expanded repeat elements. When comparing the repeat elements of *A. yamamai* with those of *B. mori*, which are both producers of the same type of silk, repeat elements showed family and species-specific patterns in the 2 silk moth linages. Particularly, we found that the mariner repeat element, which was found specifically expanded in the *A. yamamai* genome, was also included in the fibroin gene. A previous sequencing study also showed that the mariner repeat element was inserted in the 5’-end of the fibroin gene of *A. yamamai* [42]. Fibroin is the core component of the silk protein found in the silk moth, and the physical characteristics of silk mainly depend on the types and unique repeat motifs of the fibroin [43]. This gene is known to have hundreds of tandem repeat motifs, and these kinds of tandem repeats can be derived through transposable elements. This indicates that the mariner repeat element, specifically expanded in the *A. yamamai* genome, may play an important role in the development of the unique silk of *A. yamamai*, and the lineage-specific repeat elements may be one of the candidate evolution forces related to host-specific phenotype during genome evolution.

**Table 6:** Summary of identified repeat elements in the *A. yamamai* genome

Repeat element	No. of elements	Length, %
SINE	59 968	8 615 338 (1.30)
LINE	426 522	101 251 176 (15.31)
LTR element	53 977	4 552 386 (0.69)
DNA element	512 760	69 071 227 (10.44)
Small RNA	43 645	6 691 619 (1.01)
Simple repeat	135 989	6 256 839 (0.95)
Low complexity	19 937	932 829 (0.14)
Unclassified	294 190	54 552 009 (8.25)

### Gene prediction and annotation

Three different algorithms were used for gene prediction of the *A. yamamai* genome: *ab initio*, RNA-seq transcript-based, and protein homology-based approaches. For *ab initio* gene prediction, Augustus (Augustus: Gene Prediction, RRID:SCR_008417) [44], Geneid [45], and GeneMarks-ET [46] were employed. Augustus was trained using known genes of *A. yamamai* in the NCBI database, and mapping information of RNA-seq data obtained from Tophat (TopHat, RRID:SCR_013035) [47] was also utilized for gene prediction. Geneid was used with predefined parameters for *Drosophila melanogaster*. GeneMarks-ET was employed using junction information of genes from transcriptome data alignment. For RNA-seq transcript-based prediction, generated transcriptome data from 10 organ tissues of *A. yamamai* were aligned to the assembled genome and gene information was predicted using Cufflinks (Cufflinks, RRID:SCR_014597) [48]. The longest CDS sequences were identified from Cufflinks results using Transdecoder. For the homology-based approach, all known genes of the order Lepidoptera in the NCBI database were aligned using PASA [49]. Table 7 shows the gene prediction results from each method. Gene prediction results from different prediction algorithms were combined using Evidence Modeler [50], and a consensus gene set of the *A. yamamai* genome was created. Manual curation was performed based on the 5 types of evidence (3 *in silico*, known protein, and RNA-seq) using IGV [51] and BLASTP (BLASTP, RRID:SCR_001010). Using IGV with each gene evidence and comparing results with known genes via BLASTP, we mainly focused on removing false positively predicted genes that don’t have enough evidence. Merged and spliced gene structured were corrected by comparing the gene structure with known exon structure in the NCBI NR database. In addition, fibroin and sericin genes, which couldn’t be properly predicted because of their high repeat motif, were also manually identified with previously known sequences [42, 52] with RNA-seq data. The final gene set of the *A. yamamai* genome contains 15 481 genes. Summary statistics for the consensus gene set are provided in Table 8. The average gene length was 11 016.34 bp with a 34.38% GC ratio, and the number of exons per gene was 5.64. In order to identify the function of predicted genes in A yamamai, 3 nonredundant sequence databases (Swiss-Prot [53], Uniref100 [53], and NCBI NR [54]) as well as the gene information of 2 species (*B. mori* and *D. melanogaster*) were used for target databases using BLASTP. Additionally, protein domain searches were conducted on the consensus gene set using InterproScan5 (InterProScan, RRID:SCR_005829) [55]. Figure S1 shows the top 20 identified terms from 7 different InterproScan5 analyses. Among the various analyses conducted using InterproScan5, gene ontology analysis with the Pfam database showed that a large proportion of genes in the *A. yamamai* genome were related with the function of molecular binding, catalytic activity, internal component of membrane, metabolic process, oxidation-reduction process, and transmembrane transport.

**Table 7:** Summary statistics of *ab initio*, RNA-seq-based, and homology-based gene prediction results

Evidence type	Programs	Element	Total count	Exon/gene	Total length, bp	Mean length, bp
		Gene	14 576		142 415 318	9770.53
	Augustus			4.85		
		Exon	70 733		14 736 668	208.34
		Gene	10 946		46 119 402	4213.35
*ab_initio*	Geneid			2.25		
		Exon	24 686		3 925 563	159.01
		Gene	27 754		273 745 951	9863.29
	GeneMarks-ET			5.50		
		Exon	152 660		30 847 503	202.06
		Gene	36 213		840 429 061	23 207.94
RNA-seq	Cufflinks Transdecoder			7.03		
		Exon	254 770		201 721 675	791.77
Known gene (NCBI lepidoptera)	PASA (gmap)		44 561		22 484 151	504.57

**Table 8:** Summary statistics for the consensus gene set of the *A. yamamai* genome

Element	No. of elements	Exon/gene	Avg. length	Total length	Genome coverage, %
Gene	15 481		11 016.34	170 543 958	25.78
		5.64			
Exon	87 346		1346.23	20 840 925	3.31

### Comparative genome analysis

**Figure 4:**
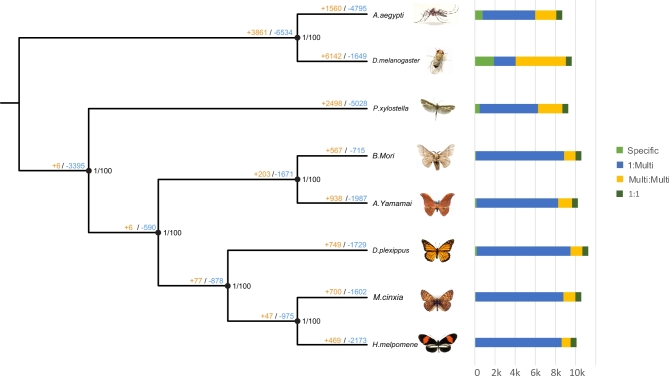
Constructed phylogenetic tree and comparative gene family analysis. Node values indicate Bayesian posterior probability, bootstrap and gene expansion, and contraction value. Orange and blue colors indicate expansion and contraction, respectively. Bar chart indicates the number of genes cauterized into 4 groups (Specific, 1:Multi, Multi:Multi, and 1:1) using OrthoMCL.

We used OrthoMCL [56] and Reciprocal Best Hit within BLASTP for identification of gene family clusters and 1:1 orthologous gene sets. Gene information of 7 taxa (*A. aegypti, B. mori, D. plexippus, D. melanogaster, H. melpomene, M. cinxia*, and *P. xylostella*), the same taxa used in repeat analysis, was employed for OrthoMCL with *A. yamamai*. A total of 17 406 gene family clusters were constructed, and 3586 1:1 orthologous genes were identified. Before conducting comparative genome analysis, we constructed phylogenetic trees for the 8 species. In order to build the phylogenetic tree, multiple sequence alignment for the 1:1 orthologous genes of all 8 species was conducted using PRANK [57], and Gblocks [58] was used to obtain conserved blocks for the phylogenetic tree. Conserved block sequences were sequentially concatenated to obtain 1 consensus sequence for each species. MEGA [59] was used for constructing Neighbor-Joining Trees (bootstrap 1000, maximum composite likelihood, transitions + transversions, and gamma distributed option), and MrBayes (MrBayes, RRID:SCR_012067) [60] was employed for the construction of Bayesian inference trees. To select the best evolution model for our data, Modeltest [61] was conducted and the GTR-based invariant model was chosen based on the AIC value of Modeltest. Figure 4 shows the constructed phylogenetic tree of the 8 species using 3586 orthologous genes. The bootstrap value and Bayesian poster probability value of all nodes were 100 and 1, respectively. The closest neighbor of *A. yamamai* was *B. mori*, which is included in Bombycidae family; this result is consistent with that of previous studies. Three butterfly species (*D. plexippus, M. cinxia*, and *H. meplmene*) included in Nymphalidae family were also shown to share a common ancestor with the families Saturniidae and Bombycidae.

**Figure 5:**
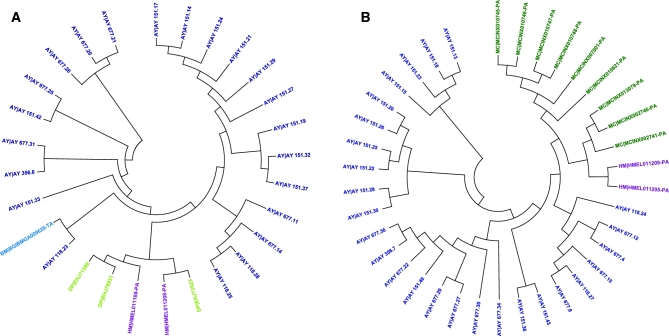
Expansion of chorion gene in the *A. yamamai* genome. A, B) The gene trees of chorion A and B in the rapid expanded gene family cluster, respectively. Color of terminal node indicates each taxon identified in the gene family cluster.

Based on the constructed phylogenetic tree, gene family expansion and contraction analysis were conducted using a 2-parameter model in CAFÉ [62] and the gene tree was constructed using protein sequence via MEGA [59]. Figure 4 shows the result of gene family expansion and contraction analysis of 8 species; 938 and 1987 gene families of *A. yamamai* and 567 and 715 gene families of *B. mori* were estimated to be expanded and contracted from the common ancestors, respectively. Among these, 15 gene families in *A. yamamai* were estimated to be under rapid expansion during the evolution process. Functions of genes in the rapidly expanded gene families of *A. yamamai* were transposase, fatty acid synthase, zinc finger protein, chorion (eggshell protein), reverse transcriptase, prostaglandin dehydrogenase, RNA-directed DNA polymerase, gag like protein, juvenile hormone acid methyltransferase, facilitated trehalose transporter, and glucose dehydrogenase. Figure 5 shows the gene tree of 2 chorion gene (chorion class A and B) family clusters rapidly expanded in the *A. yamamai* genome. Chorion, called eggshell protein, composes the surface of the egg and protects the embryo from environmental threats such as desiccation, flooding, freezing, infection of microorganisms, and physical destruction. It also provides channels, such as aeropyle, that enable gas exchange and maintain proper conditions for diapause of the egg [63]. These diverse functions of eggshell are implemented by the specific eggshell structure, and the surface structure of eggshell varies between species for the adaptation in a different environment. The ancestor of Antherea has the unique aeropyle structure called “aerophyle crown” on the eggshell surface [64]. This unique structure is formed by the circular vertical projection of lamellar chorion from the follicle cell, and it surrounds the aeropyles near the end of oogenesis [65]. Acquiring this kind of *de novo* complex structure requires numerous genetic changes, and a previous study about *Antheraea polyphemus* has shown that more than 100 chorion-specific polypeptides were involved in this unique ultra-structure [65]. Therefore, the specific rapid expansion of the chorion class A and B gene family in the *A. yamamai* genome might be one of the convincing molecular explanations for acquiring this unique ultrastructure in the eggshell surface of Antheraea genus. However, this unique ultrastructure tends to be reduced during the current evolution process of the Antheraea genus. Types of eggshell structures in the Antheraea genus can be categorized into multiple classes based on the morphology and regional distribution of the aeropyle [64]. The shape of the aeropyle in the *A. yamamai* egg is known to be converted to a mound shape from the crown shape, and these aeropyle mounds only exist in the narrow band surrounding the micropyle region [64]. Only a very few, small aeropyle crowns remain, and it is entirely different from the ancestral form of eggshell surface mostly covered by aeropyle crowns. These regional differences were known to be adjusted by regional differences of filler genes during choriogenesis [66], and the additional regulations of related genes for choriogenesis have to be considered. This indicates that the specifically expanded chorion gene families of *A. yamamai* may be one of the remaining evolutionary tracks in the genome of the Antheraea genus. However, further functional studies must be conducted to resolve the limited understanding about the relationship between these expanded chorion gene families and the current eggshell surface formation of *A. yamamai.*

**Figure 6:**
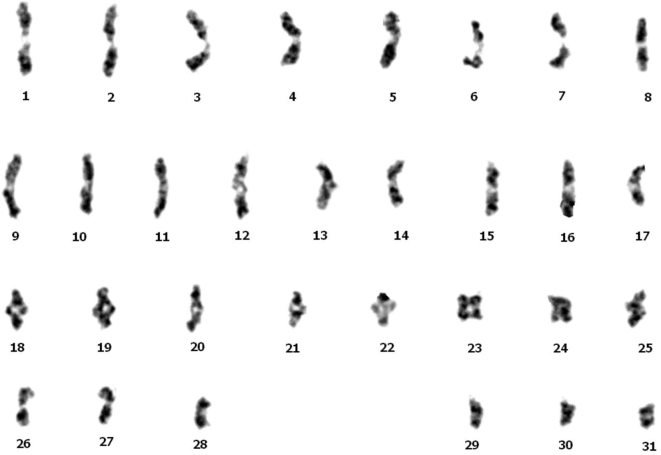
Karyotype of *A. yamamai* using a gamete of testis in metaphase.

The constructed genome of *A. yamamai* presented here is the first announced genome in the family Saturniidae, and the karyotyping analysis using gamete in the metaphase showed that the genome of *A. yamamai* consists of 31 chromosomes (Fig. 6). This constructed genome information provides more insight into the genome evolution and phylogeny of the family Saturniidae, which contains the largest number of species in Lepidoptera. For example, although 2 silk moths, *A. yamamai* and *B. mori*, appear similar, comparative genome analysis showed the significant differences in the genome size and the specific expansion of repeat elements and gene families between the families Saturniidae and Bombycidae. In case of molecular phylogeny, most previous phylogenetic studies were limited to few genes due to the lack of genomic information on the family Saturniidae. We expect that our study and resulting constructed genome will resolve some limitations of molecular phylogenetic and ecological research on the Saturniidae species. Additionally, constructed genome information will help researchers better understand the molecular background of wild silk and its production. Silk produced by *A. yamamai*, referred to as tensan silk, shows unique characteristics that have made it valuable in various fields. However, *A. yamam*ai has not been completely domesticated compared with *B. mori*, making mass production of tensan silk infeasible. Understanding of the molecular mechanisms behind the tensan silk production process is essential for mass production using biotechnology, and this genome sequence with manually curated gene information is a fundamental resource for related research and industrial improvement. Additionally, the transcriptome data of 10 different organ tissues with the 3 biological replications presented here may be also useful resources for uncovering the molecular mechanisms related to specific phenotypes of *A. yamamai* and the family Saturniidae.

## Availability of supporting data

The generated genome sequence and gene information of *A. yamamai* are available in *Giga*DB [67], and generated raw data are available under project accession PRJNA383008 and PRJNA383025 of the NCBI database.

## Additional file

Additional file 1: Figure S1. Top 20 terms in each 7 Interproscan5 analysis (CDD, Pfam, PRINTS, ProSitePatterns, ProSiteProfiles, TIGRF, Gene Ontology).

Additional file 1: Supplementrary_information2.xlsx.

## Competing interests

All authors report no competing interests.

## Abbreviations

BUSCO: Benchmarking Universal Single-Copy Orthologs; MYA: million years ago.

## Author contributions

Sampling: Kee-Young Kim, Su-Bae Kim; sequencing: Kwang-Ho Choi, Seong-Wan Kim genome assembly: Seong-Ryul Kim, Woori Kwak, Jae-Sam Hwang, Seung-Won Park repeat element analysis: Seong-Ryul Kim, Woori Kwak, Seung-Won Park gene prediction: Seong-Ryul Kim, Woori Kwak, Hyaekang Kim, Jae-Sam Hwang comparative genome analysis: Seong-Ryul Kim, Woori Kwak, Min-Jae Kim, Kelsey Caetano-Anolles funding and experimental design: Seong-Ryul Kim, Seung-Won Park.

## Supplementary Material

GIGA-D-17-00085_Original-Submission.pdf

GIGA-D-17-00085_Revision-1.pdf

GIGA-D-17-00085_Revision-2.pdf

GIGA-D-17-00085_Revision-3.pdf

Response-to-Reviewer-Comments_Original-Submission.pdf

Response-to-Reviewer-Comments_Revision-1.pdf

Response-to-Reviewer-Comments_Revision-2.pdf

Reviewer-1-Report-(Revision-1) -- Reuben William Nowell 09 Jun 2017 Reviewed

Reviewer-2-Report-(Revision-1) -- Simon Baxter 17 Jun 2017 Reviewed

Reviewer-2-Report-(Revision-2) -- Simon Baxter 18 Jun 2017 Reviewed

Additional files

## References

[1] Regier JC, Grant MC, Mitter C Phylogenetic relationships of wild silkmoths (Lepidoptera: Saturniidae) inferred from four protein-coding nuclear genes. Syst Entomol 2008;33(2):219–28.

[2] Regier JC, Mitter C, Zwick A A large-scale, higher-level, molecular phylogenetic study of the insect order Lepidoptera (moths and butterflies). PLoS One 2013;8(3):e58568. 23554903 10.1371/journal.pone.0058568 PMC3595289

[3] Hedges SB, Dudley J, Kumar S TimeTree: a public knowledge-base of divergence times among organisms. Bioinformatics 2006;22(23):2971–2. 17021158 10.1093/bioinformatics/btl505

[4] Kawahara AY, Barber JR Tempo and mode of antibat ultrasound production and sonar jamming in the diverse hawkmoth radiation. Proc Natl Acad Sci U S A 2015;112(20):6407–12. 25941377 10.1073/pnas.1416679112 PMC4443353

[5] Peigler RS Wild Silks of the World. Am Entomol 1993;39(3):151–62.

[6] Matsumoto Y-I, Saito H Load-extension characteristics of composite raw silk of *Antheraea yamamai* and *Bombyx mori*. J Sericult Sci Japan 1997;66(6):497–501.

[7] Nakamura S, Saegusa Y, Yamaguchi Y Physical properties and structure of silk. XI. Glass transition temperature of wild silk fibroins. J Appl Polym Sci 1986;31(3):955–6.

[8] Kweon H, Park Y Structural characteristics and physical properties of wild silk fibres; *Antheraea pernyi* and *Antheraea yamamai*. Korean J Sericult Sci (Korea Rep) 1994.

[9] Zheng Z, Wei Y, Yan S Preparation of regenerated *Antheraea yamamai* silk fibroin film and controlled-molecular conformation changes by aqueous ethanol treatment. J Appl Polym Sci 2010;116(1):461–7.

[10] Omenetto F, Kaplan D, Amsden J Silk based biophotonic sensors. 2011 Google Patents.

[11] Takeda S New field of insect science: research on the use of insect properties. Entomol Sci 2013;16(2):125–35.

[12] Omenetto F, Kaplan DL Silk-based multifunctional biomedical platform. 2012 Google Patents.

[13] Serban MA Silk medical devices. 2016 Google Patents.

[14] Jiang G-L, Collette AL, Horan RL Drug delivery platforms comprising silk fibroin hydrogels and uses thereof. 2010 Google Patents.

[15] Kamiya M, Oyauchi K, Sato Y Structure-activity relationship of a novel pentapeptide with cancer cell growth-inhibitory activity. J Pept Sci 2010;16(5):242–8. 20401925 10.1002/psc.1225

[16] Bioinformatics B FastQC A Quality Control Tool for High Throughput Sequence Data. Cambridge, UK: Babraham Institute, 2011.

[17] Bolger AM, Lohse M, Usadel B Trimmomatic: a flexible trimmer for Illumina sequence data. Bioinformatics 2014:btu170. 10.1093/bioinformatics/btu170 PMC4103590 24695404

[18] Marçais G, Kingsford C A fast, lock-free approach for efficient parallel counting of occurrences of k-mers. Bioinformatics 2011;27(6):764–70. 21217122 10.1093/bioinformatics/btr011 PMC3051319

[19] You M, Yue Z, He W A heterozygous moth genome provides insights into herbivory and detoxification. Nat Genet 2013;45(2):220–5. 23313953 10.1038/ng.2524

[20] Maruyama T, Nei M Genetic variability maintained by mutation and overdominant selection in finite populations. Genetics 1981;98(2):441–59. 17249094 10.1093/genetics/98.2.441 PMC1214452

[21] Pamilo P, Pálsson S Associative overdominance, heterozygosity and fitness. Heredity 1998;81(4):381–9. 9839436 10.1046/j.1365-2540.1998.00395.x

[22] Gnerre S, MacCallum I, Przybylski D High-quality draft assemblies of mammalian genomes from massively parallel sequence data. Proc Natl Acad Sci U S A 2011;108(4):1513–8. 21187386 10.1073/pnas.1017351108 PMC3029755

[23] Luo R, Liu B, Xie Y SOAPdenovo2: an empirically improved memory-efficient short-read de novo assembler. Gigascience 2012;1(1):18. 23587118 10.1186/2047-217X-1-18 PMC3626529

[24] O’Connell J, Schulz-Trieglaff O, Carlson E NxTrim: optimized trimming of Illumina mate pair reads: Table 1. Bioinformatics 2015;31(12):2035–7. 25661542 10.1093/bioinformatics/btv057

[25] Boetzer M, Henkel CV, Jansen HJ Scaffolding pre-assembled contigs using SSPACE. Bioinformatics 2011;27(4):578–9. 21149342 10.1093/bioinformatics/btq683

[26] Boetzer M, Pirovano W SSPACE-LongRead: scaffolding bacterial draft genomes using long read sequence information. BMC Bioinformatics 2014;15(1):211. 24950923 10.1186/1471-2105-15-211 PMC4076250

[27] Voskoboynik A, Neff NF, Sahoo D The genome sequence of the colonial chordate, *Botryllus schlosseri*. Elife 2013;2:e00569. 23840927 10.7554/eLife.00569 PMC3699833

[28] McCoy RC, Taylor RW, Blauwkamp TA Illumina TruSeq synthetic long-reads empower de novo assembly and resolve complex, highly-repetitive transposable elements. PLoS One 2014;9(9):e106689. 25188499 10.1371/journal.pone.0106689 PMC4154752

[29] Simão FA, Waterhouse RM, Ioannidis P BUSCO: assessing genome assembly and annotation completeness with single-copy orthologs. Bioinformatics 2015:btv351. 10.1093/bioinformatics/btv351 26059717

[30] Bao Z, Eddy SR Automated de novo identification of repeat sequence families in sequenced genomes. Genome Res 2002;12(8):1269–76. 12176934 10.1101/gr.88502 PMC186642

[31] Price AL, Jones NC, Pevzner PA De novo identification of repeat families in large genomes. Bioinformatics 2005;21(Suppl 1):i351–8. 15961478 10.1093/bioinformatics/bti1018

[32] Benson G Tandem repeats finder: a program to analyze DNA sequences. Nucleic Acids Res 1999;27(2):573–80. 9862982 10.1093/nar/27.2.573 PMC148217

[33] Kohany O, Gentles AJ, Hankus L Annotation, submission and screening of repetitive elements in Repbase: RepbaseSubmitter and Censor. BMC Bioinformatics 2006;7(1):474. 17064419 10.1186/1471-2105-7-474 PMC1634758

[34] Tarailo-Graovac M, Chen N Using RepeatMasker to identify repetitive elements in genomic sequences. Curr Protoc Bioinformatics 2009:4.10.1–4.10.14. 10.1002/0471250953.bi0410s25 19274634

[35] Bao W, Kojima KK, Kohany O Repbase Update, a database of repetitive elements in eukaryotic genomes. Mobile DNA 2015;6(1):11. 26045719 10.1186/s13100-015-0041-9 PMC4455052

[36] Nene V, Wortman JR, Lawson D Genome sequence of *Aedes aegypti*, a major arbovirus vector. Science 2007;316(5832):1718–23. 17510324 10.1126/science.1138878 PMC2868357

[37] Xia Q, Zhou Z, Lu C A draft sequence for the genome of the domesticated silkworm (*Bombyx mori*). Science 2004;306(5703):1937–40. 15591204 10.1126/science.1102210

[38] Zhan S, Merlin C, Boore JL The monarch butterfly genome yields insights into long-distance migration. Cell 2011;147(5):1171–85. 22118469 10.1016/j.cell.2011.09.052 PMC3225893

[39] Adams MD, Celniker SE, Holt RA The genome sequence of *Drosophila melanogaster*. Science 2000;287(5461):2185–95. 10731132 10.1126/science.287.5461.2185

[40] Consortium HG Butterfly genome reveals promiscuous exchange of mimicry adaptations among species. Nature 2012;487(7405):94–98. 22722851 10.1038/nature11041 PMC3398145

[41] Ahola V, Lehtonen R, Somervuo P The Glanville fritillary genome retains an ancient karyotype and reveals selective chromosomal fusions in Lepidoptera. Nat Commun 2014;5. 10.1038/ncomms5737 PMC4164777 25189940

[42] Hwang J-S, Lee J-S, Goo T-W Cloning of the fibroin gene from the oak silkworm, *Antheraea yamamai* and its complete sequence. Biotechnol Lett 2001;23(16):1321–6.

[43] Malay AD, Sato R, Yazawa K Relationships between physical properties and sequence in silkworm silks. Sci Rep 2016;6(1):27573. 27279149 10.1038/srep27573 PMC4899792

[44] Stanke M, Diekhans M, Baertsch R Using native and syntenically mapped cDNA alignments to improve de novo gene finding. Bioinformatics 2008;24(5):637–44. 18218656 10.1093/bioinformatics/btn013

[45] Blanco E, Parra G, Guigó R Using Geneid to identify genes. Curr Protoc Bioinformatics 2007:4.3.1–4.3.28. 10.1002/0471250953.bi0403s18 18428791

[46] Lomsadze A, Burns PD, Borodovsky M Integration of mapped RNA-Seq reads into automatic training of eukaryotic gene finding algorithm. Nucleic Acids Res 2014:gku557. 10.1093/nar/gku557 PMC4150757 24990371

[47] Trapnell C, Pachter L, Salzberg SL TopHat: discovering splice junctions with RNA-Seq. Bioinformatics 2009;25(9):1105–11. 19289445 10.1093/bioinformatics/btp120 PMC2672628

[48] Trapnell C, Roberts A, Goff L Differential gene and transcript expression analysis of RNA-seq experiments with TopHat and Cufflinks. Nat Protoc 2012;7(3):562–78. 22383036 10.1038/nprot.2012.016 PMC3334321

[49] Campbell MA, Haas BJ, Hamilton JP Comprehensive analysis of alternative splicing in rice and comparative analyses with Arabidopsis. BMC Genomics 2006;7(1):327. 17194304 10.1186/1471-2164-7-327 PMC1769492

[50] Haas BJ, Salzberg SL, Zhu W Automated eukaryotic gene structure annotation using EVidenceModeler and the program to assemble spliced alignments. Genome Biol 2008;9(1):R7. 18190707 10.1186/gb-2008-9-1-r7 PMC2395244

[51] Robinson JT, Thorvaldsdóttir H, Winckler W Integrative genomics viewer. Nat Biotechnol 2011;29(1):24–26. 21221095 10.1038/nbt.1754 PMC3346182

[52] Zurovec M, Yonemura N, Kludkiewicz B Sericin Composition in the Silk of *Antheraea yamamai*. Biomacromolecules 2016;17(5):1776–87. 27049111 10.1021/acs.biomac.6b00189

[53] Consortium U Reorganizing the protein space at the Universal Protein Resource (UniProt). Nucleic Acids Res 2011:gkr981. 10.1093/nar/gkr981 PMC3245120 22102590

[54] Pruitt KD, Tatusova T, Maglott DR NCBI reference sequences (RefSeq): a curated non-redundant sequence database of genomes, transcripts and proteins. Nucleic Acids Res 2007;35(Database):D61–5. 17130148 10.1093/nar/gkl842 PMC1716718

[55] Jones P, Binns D, Chang H-Y InterProScan 5: genome-scale protein function classification. Bioinformatics 2014;30(9):1236–40. 24451626 10.1093/bioinformatics/btu031 PMC3998142

[56] Li L, Stoeckert CJ, Roos DS OrthoMCL: identification of ortholog groups for eukaryotic genomes. Genome Res 2003;13(9):2178–89. 12952885 10.1101/gr.1224503 PMC403725

[57] Löytynoja A, Goldman N From The Cover: An algorithm for progressive multiple alignment of sequences with insertions. Proc Natl Acad Sci U S A 2005;102(30):10557–62. 16000407 10.1073/pnas.0409137102 PMC1180752

[58] Castresana J Selection of conserved blocks from multiple alignments for their use in phylogenetic analysis. Mol Biol Evol 2000;17(4):540–52. 10742046 10.1093/oxfordjournals.molbev.a026334

[59] Kumar S, Stecher G, Tamura K MEGA7: Molecular evolutionary genetics analysis version 7.0 for bigger datasets. Mol Biol Evol 2016;33(7):1870–4. 27004904 10.1093/molbev/msw054 PMC8210823

[60] Ronquist F, Huelsenbeck JP *MrBayes 3:* Bayesian phylogenetic inference under mixed models. Bioinformatics 2003;19(12):1572–4. 12912839 10.1093/bioinformatics/btg180

[61] Posada D Using MODELTEST and PAUP* to select a model of nucleotide substitution. Curr Protoc Bioinformatics 2003:6.5.1–6.5.14. 10.1002/0471250953.bi0605s00 18428705

[62] De Bie T, Cristianini N, Demuth JP CAFE: a computational tool for the study of gene family evolution. Bioinformatics 2006;22(10):1269–71. 16543274 10.1093/bioinformatics/btl097

[63] Chapman RF The Insects: Structure and Function. Cambridge: Cambridge University Press; 1998.

[64] Regier JC, Paukstadt U, Paukstadt LH Phylogenetics of eggshell morphogenesis in Antheraea (Lepidoptera: Saturniidae): unique origin and repeated reduction of the aeropyle crown. Syst Biol 2005;54(2):254–67. 16012096 10.1080/10635150590923281

[65] Regier JC, Mazur GD, Kafatos FC The silkmoth chorion: morphological and biochemical characterization of four surface regions. Devel Biol 1980;76(2):286–304. 7390006 10.1016/0012-1606(80)90380-2

[66] Hatzopoulos AK, Regier JC Evolutionary changes in the developmental expression of silkmoth chorion genes and their morphological consequences. Proc Natl Acad Sci U S A 1987;84(2):479–83. 3467368 10.1073/pnas.84.2.479 PMC304232

[67] Kim S, Kwak W, Kim K The Japanese silk moth, *Antheraea yamamai*, draft genome sequence. GigaScience Database 2017 http://dx.doi.org/10.5524/100382. 10.1093/gigascience/gix113 PMC5774507 29186418

