# Supplementary material for: Genome sequence of the Japanese oak silk moth, Antheraea yamamai: the first draft genome in the family Saturniidae
Source: Gigascience. 2017 Nov 27;7(1):gix113. doi: 10.1093/gigascience/gix113 (PMC5774507; doi:10.1093/gigascience/gix113)
Supplement: GIGA-D-17-00085_Original-Submission.pdf [file gix113_giga-d-17-00085_original-submission.pdf]

GigaScience

First wild silkworm genome of Japanese silk moth, Antheraea yamamai

--Manuscript Draft--

**Manuscript Number:**	GIGA-D-17-00085
**Full Title:**	First wild silkworm genome of Japanese silk moth, Antheraea yamamai
**Article Type:**	Data Note
**Funding Information:**	Rural Development Administration (PJ010442) — Dr Seong-Ryul Kim
**Abstract:**	Background<br>Antheraea yamamai is one of wild silkworm species known as Japanese silk moth. Silk of A. yamamai, called tensan silk, shows large differences characteristics compared to common silk produced from domesticated silkworm, Bombyx mori. Therefore, it is utilized in many research fields including biotechnology and medical science, and scientific as well as economic importance of wild silkworm is constantly increasing. However, no genomic information for wild silkworm including A. yamamai is currently available.<br><br>Findings<br>For constructing the A. yamamai genome, a total of 147G base pairs using Illumina and Pacbio sequencing platforms were generated and it was 210-fold coverage based on the 700 Mb estimated genome size of A. yamamai. Assembled genome of A. yamamai was 656 Mb(>2kb) with 3,675 scaffolds and N50 length of assembly was 739 Kb with 34.07% GC ratio. Identified repeat element covered 37.33% of total genome and the completeness of genome assembly was estimated to be 96.7% by BUSCO v2 analysis. A total of 21,124 genes were identified using Evidence Modeler based on the gene prediction results from 3 different methods (ab initio, RNA-seq based, known gene based).<br><br>Conclusions<br>Here we present the genome sequence of A. yamamai, the first genome sequence of wild silkworm. Our result will provide valuable genomic information for understanding the molecular mechanisms related to the specific phenotypes such as wild silk itself, and more insight into Saturniidae evolution process.
**Corresponding Author:**	Seung-Won Park<br><br>KOREA, REPUBLIC OF
**Corresponding Author Secondary Information:**	
**Corresponding Author's Institution:**	
**Corresponding Author's Secondary Institution:**	
**First Author:**	Seong-Ryul Kim
**First Author Secondary Information:**	
**Order of Authors:**	Seong-Ryul Kim
	Woori Kwak
	Kee-Young Kim
	Su-Bae Kim
	Kwang-Ho Choi
	Seong-Wan Kim
	Jae-Sam Hwang
	Iksoo Kim
	Tae-Won Goo

Powered by Editorial Manager® and ProduXion Manager® from Aries Systems Corporation

	Seung-Won Park
**Order of Authors Secondary Information:**	
**Opposed Reviewers:**	
**Additional Information:**	
**Question**	**Response**
Are you submitting this manuscript to a special series or article collection?	No
**Experimental design and statistics**<br><br>Full details of the experimental design and statistical methods used should be given in the Methods section, as detailed in our Minimum Standards Reporting Checklist. Information essential to interpreting the data presented should be made available in the figure legends.<br><br>Have you included all the information requested in your manuscript?	Yes
**Resources**<br><br>A description of all resources used, including antibodies, cell lines, animals and software tools, with enough information to allow them to be uniquely identified, should be included in the Methods section. Authors are strongly encouraged to cite Research Resource Identifiers (RRIDs) for antibodies, model organisms and tools, where possible.<br><br>Have you included the information requested as detailed in our Minimum Standards Reporting Checklist?	Yes
**Availability of data and materials**<br><br>All datasets and code on which the conclusions of the paper rely must be either included in your submission or deposited in publicly available repositories (where available and ethically appropriate), referencing such data using a unique identifier in the references and in the "Availability of Data and Materials" section of your manuscript.<br><br>Have you have met the above requirement as detailed in our Minimum Standards Reporting Checklist?	Yes

Powered by Editorial Manager® and ProduXion Manager® from Aries Systems Corporation

Powered by Editorial Manager® and ProduXion Manager® from Aries Systems Corporation

Manuscript Click here to download Manuscript Manuscript_FIN.docx

First wild silkworm genome of Japanese silk moth, *Antheraea yamamai*

Seong-Ryul Kim[1†], Woori Kwak[2†], Kee-Young Kim[1], Su-Bae Kim[1], Kwang-Ho Choi[1], Seong-Wan Kim[1], Jae-Sam Hwang[1], Iksoo Kim[3], Tae-Won Goo[4] and Seung-Won Park[5]*

[1]Department of Agricultural Biology, National Academy of Agricultural Science, Rural Development Administration, Wanju-gun 55365, Republic of Korea; [2]C&K Genomics, Main Bldg. #420, SNU Research Park, Seoul 151-919, Republic of Korea; [3]College of Agriculture & Life Sciences, Chonnam National University, Gwangju, Republic of Korea; [4]Department of Biochemistry, Dongguk University College of Medicine, Gyeongju-si, Gyeongsangbuk-do 38066, Republic of Korea; [5]Department of Biotechnology, Catholic University of Daegu, Gyeongsan-si, Gyeongsangbuk-do 38430, Republic of Korea

Seong-Ryul Kim : ksr319@korea.kr; Woori Kwak : asleo@cnkgenomics.com; Kee-Young Kim : kkyoung@korea.kr; Su-Bae Kim : subae@korea.kr; Kwang-Ho Choi : ckh@korea.kr; Seong-Wan; Seong-Wan Kim : tarupa@korea.kr; Jae-Sam Hwang : hwangjs@korea.kr; Iksoo Kim : ikkim81@chonnam.ac.kr; Tae-Won Goo : gootw@dongguk.ac.kr

[†] These authors equally contributed and should be regarded as co-first authors.

* Corresponding authors

Seung-Won Park

Department of Biotechnology,

Catholic University of Daegu,

Gyeongsan-si, Gyeongsangbuk-do 38430, Republic of Korea

Phone : +82-53-850-3176

Fax : +82-53-359-6846

E-mail: microsw@cu.ac.kr

Abstract

Background

Antheraea yamamai is one of wild silkworm species known as Japanese silk moth. Silk of *A. yamamai*, called *tensan* silk, shows large differences characteristics compared to common silk produced from domesticated silkworm, *Bombyx mori*. Therefore, it is utilized in many research fields including biotechnology and medical science, and scientific as well as economic importance of wild silkworm is constantly increasing. However, no genomic information for wild silkworm including *A. yamamai* is currently available.

Findings

For constructing the *A. yamamai* genome, a total of 147G base pairs using Illumina and Pacbio sequencing platforms were generated and it was 210-fold coverage based on the 700 Mb estimated genome size of *A. yamamai*. Assembled genome of *A. yamamai* was 656 Mb(>2kb) with 3,675 scaffolds and N50 length of assembly was 739 Kb with 34.07% GC ratio. Identified repeat element covered 37.33% of total genome and the completeness of genome assembly was estimated to be 96.7% by BUSCO v2 analysis. A total of 21,124 genes were identified using Evidence Modeler based on the gene prediction results from 3 different methods (*ab initio*, RNA-seq based, known gene based).

Conclusions

Here we present the genome sequence of *A. yamamai*, the first genome sequence of wild silkworm. Our result will provide valuable genomic information for understanding the molecular mechanisms related to the specific phenotypes such as wild silk itself, and more insight into Saturniidae evolution process.

Keywords

Antheraea yamamai, Japanese silk moth, Japanese oak silkmoth, wild silkworm

Data description

Antheraea yamamai (Figure 1) known as Japanese silk moth is one of wild silkworm species belongs to the Saturniidae family. The most specific phenotypic trait of this species is silk, called tensan silk[1]. It shows characteristics such as thickness, bulkiness, compressive elasticity, resistance to chemicals compared to common silk from domesticated silkworm[2-4], *Bombyx mori*, and it is utilized as a new biomaterial for various fields[5-7]. In addition, various studies have been in place using peptides from *A. yamamai* for human health[8-11]. In spite of these academic and economical importance of wild silkworm including *A. yamamai*, however, no genomic information is currently available for any wild silkworm species. In this study, we present the first wild silkworm genome, *A. yamamai*, with gene expression data of ten different body organ tissues. We expect that the first wild silkworm genome for *A. yamamai* can be the fundamental genomic resource for various wild silkworm researches, and our data will provide more insight into the underlying molecular mechanisms of silk production process in wild silkworms and its specific characteristics.

Sequencing

For whole genome sequencing, we selected one male sample(Ay-7-male1) from the breeding line(Ay-7) of *A. yamamai* in National Academy of Agricultural Science, Rural Development Administration, Korea. Before conducting sequencing analysis, we conducted karyotyping analysis to confirm the number of chromosomes and chromosome abnormality using a gamete in metaphase. Figure 2 shows the result of karyotyping analysis and the genome of Japanese Silkworm consist of 31 chromosomes. Before DNA isolation, we removed guts of *A. yamamai* to prevent contamination of genomes from other organisms such as gut microbes and oak, the

main food of *A. yamamai*. Genomic DNA was extracted using a DNeasy Animal Mini Kit (Qiagen, Hilden, Germany) and the quality of extracted DNA was checked using trenean, picogreen assay and gel electrophoresis (1% agarose gel/ 40ng loading). We got a total 61.5ug of *A. yamamai* DNA for genome sequencing after quality control process. With the standard Illumina whole genome shotgun(WGS) sequencing protocol (paired-end and mate-pair), we added two long read sequencing platforms, Moleculo (Illumina synthetic long read) and RS II(Pacific Bioscience). Table 1-3 shows summary of generated data information for each library used in this study. And we also constructed RNA-seq libraries for genome annotation and specific gene expression of 10 different tissues (Hemocyte, Malpighi, Midgut, Fat Body, AM/Silk gland, P/Sild gland, Head, Skin, Testis, Ovary) with 3 biological replications following the standard protocol of manufacturer (Illumina, San Diego, CA, USA). For this, more than 100 individual *A. yamamai* samples in same breeding line were used for tissue anatomy and 3 samples in each tissue were selected based on the quality of extracted RNA. Information of libraries and generated data is shown in Table 4. Total 147Gb for genome and 76Gb for transcriptome data were generated for this study.

Genome assembly and evaluation

Before conducting genome assembly, we conducted k-mer distribution analysis using 350bp paired-end library to identify the genomic characteristics and estimate the genome size. Quality of generated raw data was checked with FASTQC[12]. Sequencing artifacts such as adapter sequences and low quality bases were removed using Trimmomatic[13]. Jellyfish[14] was used to count the k-mer frequency and genome size of *A. yamamai* was estimated. Figure 3 shows the 19-mer distribution of *A. yamamai* genome using 350bp paired-end library. In 19-mer

distribution, there was a second peak in the half x-axis of the main peak which indicate the heterozygosity. Our inbreeding line was maintained more than 10 generations, but high heterozygosity still remained. This phenomenon was observed in the previous genome study of black diamond moth (*Plutella xylostella*) and sustained heterozygosity is one of the important genomic characteristics related to the environmental adaption[15]. Based on the result of 19-mer distribution analysis, the genome size of *A. yamamai* genome was the estimated as approximately 709Mb. We conducted error correction for Illumina paired-end libraries using error correction module of Allpaths-LG[16] before initial contig assembly process. After error correction, initial contig assembly with 350bp and 700bp libraries was conducted using SOAP denovo2[17] with K=19 parameter option which showed best assembly statistics compared to other assemblers with various parameter. Quality control process for mate-pair libraries and scaffolding process were conducted using Nxtrim[18] and SSPACE[19], respectively. In each scaffolding step, SOAP Gapcloser[17] with -l 155 and -p 31 parameters were repeatedly used to close the gaps within each scaffold. For more high quality genome assembly of *A. yamamai*, we employed long read scaffolding strategies using SSPACE-LongRead[20]. First, we used Illumina synthetic long read sequencing platform called Moleculo which showed its value for high heterozygous genome same as *A. yamamai* in previous study[21, 22]. After scaffolding process using SSPACE-LongRead with Illumina synthetic long read data, the total number of assembled scaffolds was effectively reduced to 24,558 from 398,446. And the average scaffold length was also extended from 1.7 Kb to 24.8 Kb. However, there was no impressive improvement in N50 length (approximately 91 Kb to 112 Kb) of assembled scaffolds. Therefore, we employed another type of long read data generated from 10 cells of Pacbio RS II system with P6-C4 chemistry. After final scaffolding process using Pacbio long reads, the number of scaffolds in assembled genome was reduced to 3,675 and N50 length was effectively extended to 739 Kb from 112 Kb. Summary statistics for

assembled genome of *A. yamamai* is shown in Table 5. Final assembly of *A. yamamai* genome was 656 Mb(>2kb) with 3,675 scaffolds and N50 length of assembly was 739 Kb with 34.07% GC ratio. To evaluate the quality of assembled genome, we conducted BUSCO (Benchmarking Universal Single-Copy Orthologs) analysis[23] using BUSCO v2.0 with insecta_odb9 including 42 species and 1,658 BUSCOs. In BUSCO analysis, 96.7% of BUSCOs were completely detected (1,576 – complete and single-copy, 27 – complete and duplicated) among 1,658 tested BUSCOs in the assembled genome. The number of fragmented and missing BUSCOs was 21 and 34, respectively. Based on the result of BUSCO analysis, assembled genome of *A.yamamai* is considered properly assembled for various downstream analysis of many researchers.

Repeat identification and comparative repeat analysis

To identify the repeat element of *A. yamamai* genome, custom repeat library was constructed using RepeatModeler with RECON[24], RepeatScout[25] and TRF[26]. Constructed custom repeat library for *A. yamamai* genome was more curated using CENSOR[27] search and curated library was used in RepeatMasker[28] with Repbase[29]. RepeatMasker was employed with RMBlast and ‘no_is’ option. Table 6 shows the summary statistics of identified mobile elements and its proportion identified in A. *yamamai* genome. Most identified repeat element in *A. yamamai* genome was LINE element (101 Mb, 15.31% of total genome) and total repeat elements accounted for 37.33% of *A. yamamai* genome. To compare the repeat elements of *A. yamamai* genome with other genomes, we conducted same process for seven genomes including *Aedes aegypti*[30], *Bombyx mori*[31], *Danaus plexippus*[32], *Drosophila melanogaster[33]*, *Heliconius melpomene*[34], *Melitaea cinxia*[35] and *Plutella xylostella*[15],

available genomes among close neighbors of *A. yamamai*. Figure 4 shows the amount and proportion of identified repeat element from 8 species. Comparing repeat elements of *A. yamamai* with *B. mori*, same silk production species, the most frequently represented repeat element was SINE element in *B. mori*. Even though *A. yamamai* and *B. mori* were evolutionary close neighbor species among 8 species, types of identified repeat element in expansion showed species different pattern in silkworm linage. In more details, top 5 expanded repeat elements in *A. yamamai* genome were DNA/RC, LINE/L2, LINE/RTE-BovB, DNA/TcMar-Mariner and LINE/CR1. Among these, DNA/TcMar-Mariner was specifically expanded in *A. yamamai* among 8 species and LINE/L2 element was commonly expended in *A. yamamai* and *H. melpomene.*

Gene prediction and annotation

Three different algorithms were used for gene prediction of *A. yamamai* genome: *ab initio*, RNA-seq transcript and protein homology based. For *ab initio* gene prediction, Augustus[36], Geneid[37] and GeneMarks-ET[38] were employed. Augustus was trained using known genes of *A. yamamai* in NCBI database and mapping information of RNA-seq data using Tophat[39] was also utilized for gene prediction. Geneid was used with the predefined parameter for *Drosophila melanogaster*. GeneMarks-ET was employed with junction information of genes from transcriptome data alignment. For RNA-seq transcript based prediction, generated transcriptome data from ten organ tissues of *A. yamamai* were aligned to the assembled genome using Tophat. Gene information were predicted using Cufflinks[40] and longest CDS sequences were identified using Transdecoder. For homology-based approach, all known genes of lepidoptera order in NCBI database were aligned using PASA[41]. Table 7 shows the gene

prediction result from each method. Gene prediction results from different prediction algorithms were combined using EVM (Evidence Modeler)[42] to build a consensus gene set for the *A. yamamai* genome. Final gene set of *A. yamamai* genome contains 21,124 genes and summary statistics for the consensus gene set is provided in Table 8. To identify the function of predicted genes, Swiss-Prot[43], Uniref100[43], NCBI NR[44] database, and gene information of *B. mori* and *D. melanogaster* genes were used for sequence similarity search using blastp. And we also conducted protein domain search using InterproScan5[45]. Figure S1 shows top 20 identified terms in 10 analysis of InterproScan5. Based on gene ontology analysis, large proportion of genes in *A.yamamai* genome were related with molecule binding, digestion and transport biological process.

Demographic history and comparative genome analysis

We estimated the demographic history of *A. yamamai* using the PSMC (pairwise sequentially Markovian coalescent) method[46]. This method can infer the history of population size from a diploid sequence. 350bp paired-end reads were realigned to assembled genome using Bowtie2 and consensus sequence data was generated from read alignment data using samtools[47] with parameters -d 10, -d 100. Bootstrap sampling was also executed 100 times. For the resulting plots, generation time was set to 1 years based on the life cycle of *A. yamamai*. Figure 5a shows the inferred demographic history of *A. yamamai* using PSMC model. Based on the PSMC analysis, the results suggest that population size of *A. yamamai* species consistently increased before the last glacial period (approximately 110,000 to 12,000 years ago) same with most of insect population. During the last glacial period, population size had been continuously decreased. In Late Glacial Maximum Period (13,000 to 10,000 years ago),

which is also known as the beginning of the Modern Warm Period, population size of *A.yamamai* didn't increase and stayed at its low level.

We used OrthoMCL[48] and RBH(Reciprocal Best Hit) using blastp for gene family group analysis and 1:1 orthologous gene set identification, respectively. A total of 18,013 gene family clusters was constructed and 3,586 1:1 orthologous genes were identified. Before conducting comparative genome analysis, we constructed phylogenetic tree of 8 species. To build the phylogenetic tree, multiple sequence alignment for 1:1 orthologous genes of 8 species was conducted using PRANK[49] and Gblocks[50] was used to obtain the conserved blocks for phylogenetic tree. Conserved block sequences were sequentially concatenated to one consensus sequence for each species. MEGA6[51] was used for constructing Neighbor-Joining Tree (bootstrap 1000, maximum composite likelihood, transitions + transversions, and gamma distributed option) and MrBayes[52] was employed for Bayesian inference tree. To select the best evolution model for our data, Modeltest[53] was conducted and GTR based invariant model was chosen based on the AIC value of Modeltest. Gene family expansion and contraction analysis was conducted using CAFE[54] based on the constructed phylogenetic tree. Figure 5b shows the result of constructed phylogenetic tree and gene family analysis of 8 species. The number of expended and contracted genes of *A. yamamai* and *B. mori* indicated that there was a difference genome evolution process between two silkworm species. Gene ontology pathway analysis was conducted using gene annotation based on the *D. melanogaster* (E-value < 1E-9) with ClueGO[55] and network of enriched pathways showed in Figure S2. Function of expanded gene family was related to development and homeostasis function like hormone metabolism, imaginal disc, digestion etc. Future study about related genes will help to provide more insight into *A.yamamai* genome evolution.

Availability of supporting data

Genome sequence and gene information of *A. yamamai* are available in GigaDB[56] and generated raw data is available in project accession PRJNA383008 and PRJNA383025 of NCBI database.

Competing interests

All authors report no competing interests.

Authors contributions

Sampling - Kee-Young Kim, Su-Bae Kim

Sequencing - Kwang-Ho Choi, Seong-Wan Kim

Genome assembly - Seong-Ryul Kim, Woori Kwak, Jae-Sam Hwang, Seung-Won Park

Repeat element analysis - Seong-Ryul Kim, Woori Kwak, Seung-Won Park

Gene prediction - Seong-Ryul Kim, Woori Kwak, Jae-Sam Hwang

Comparative genome analysis - Seong-Ryul Kim, Woori Kwak

Funding and experimental design - Seong-Ryul Kim, Seung-Won Park

Acknowledgements

This work was supported by a grant from the Rural Development Administration, Republic of Korea (grant no. PJ010442).

References

1. Peigler, R.S., *Wild silks of the world.* American Entomologist, 1993. **39**(3): p. 151-162.
2. Nakamura, S., et al., *Physical properties and structure of silk. XI. Glass transition temperature of wild silk fibroins.* Journal of applied polymer science, 1986. **31**(3): p. 955-956.
3. 松本陽一 and 斎藤英毅, *Load-extension characteristics of composite raw silk of Antheraea yamamai and Bombyx mori.* 日本蚕糸学雑誌, 1997. **66**(6): p. 497-501.
4. Kweon, H. and Y. Park, *Structural characteristics and physical properties of wild silk fibres; Antheraea pernyi and Antheraea yamamai.* Korean Journal of Sericultural Science (Korea Republic), 1994.
5. Zheng, Z., et al., *Preparation of regenerated Antheraea yamamai silk fibroin film and controlled-molecular conformation changes by aqueous ethanol treatment.* Journal of applied polymer science, 2010. **116**(1): p. 461-467.
6. Omenetto, F., et al., *Silk based biophotonic sensors*. 2011, Google Patents.
7. Takeda, S., *New field of insect science: Research on the use of insect properties.* Entomological Science, 2013. **16**(2): p. 125-135.
8. Omenetto, F. and D.L. Kaplan, *Silk-based multifunctional biomedical platform*. 2012, Google Patents.
9. Serban, M.A., *Silk medical devices*. 2016, Google Patents.
10. Jiang, G.-L., et al., *Drug delivery platforms comprising silk fibroin hydrogels and uses thereof.* 2010, Google Patents.
11. Kamiya, M., et al., *Structure–activity relationship of a novel pentapeptide with cancer cell growth-inhibitory activity.* Journal of Peptide Science, 2010. **16**(5): p. 242-248.
12. Bioinformatics, B., *FastQC A quality control tool for high throughput sequence data.* Cambridge, UK: Babraham Institute, 2011.
13. Bolger, A.M., M. Lohse, and B. Usadel, *Trimmomatic: a flexible trimmer for Illumina sequence data.* Bioinformatics, 2014: p. btu170.
14. Marçais, G. and C. Kingsford, *A fast, lock-free approach for efficient parallel counting of occurrences of k-mers.* Bioinformatics, 2011. **27**(6): p. 764-770.
15. You, M., et al., *A heterozygous moth genome provides insights into herbivory and detoxification.* Nature genetics, 2013. **45**(2): p. 220-225.
16. Gnerre, S., et al., *High-quality draft assemblies of mammalian genomes from massively parallel sequence data.* Proceedings of the National Academy of Sciences, 2011. **108**(4): p. 1513-1518.
17. Luo, R., et al., *SOAPdenovo2: an empirically improved memory-efficient short-read de novo assembler.* Gigascience, 2012. **1**(1): p. 18.
18. O'Connell, J., et al., *NxTrim: optimized trimming of Illumina mate pair reads.* Bioinformatics,

2015. **31**(12): p. 2035-2037.

19. Boetzer, M., et al., *Scaffolding pre-assembled contigs using SSPACE.* Bioinformatics, 2011. **27**(4): p. 578-579.
20. Boetzer, M. and W. Pirovano, *SSPACE-LongRead: scaffolding bacterial draft genomes using long read sequence information.* BMC bioinformatics, 2014. **15**(1): p. 211.
21. Voskoboynik, A., et al., *The genome sequence of the colonial chordate, Botryllus schlosseri.* Elife, 2013. **2**: p. e00569.
22. McCoy, R.C., et al., *Illumina TruSeq synthetic long-reads empower de novo assembly and resolve complex, highly-repetitive transposable elements.* PloS one, 2014. **9**(9): p. e106689.
23. Simão, F.A., et al., *BUSCO: assessing genome assembly and annotation completeness with single-copy orthologs.* Bioinformatics, 2015: p. btv351.
24. Bao, Z. and S.R. Eddy, *Automated de novo identification of repeat sequence families in sequenced genomes.* Genome research, 2002. **12**(8): p. 1269-1276.
25. Price, A.L., N.C. Jones, and P.A. Pevzner, *De novo identification of repeat families in large genomes.* Bioinformatics, 2005. **21**(suppl 1): p. i351-i358.
26. Benson, G., *Tandem repeats finder: a program to analyze DNA sequences.* Nucleic acids research, 1999. **27**(2): p. 573-580.
27. Kohany, O., et al., *Annotation, submission and screening of repetitive elements in Repbase: RepbaseSubmitter and Censor.* BMC bioinformatics, 2006. **7**(1): p. 474.
28. Tarailo-Graovac, M. and N. Chen, *Using RepeatMasker to identify repetitive elements in genomic sequences.* Current Protocols in Bioinformatics, 2009: p. 4.10. 1-4.10. 14.
29. Bao, W., K.K. Kojima, and O. Kohany, *Repbase Update, a database of repetitive elements in eukaryotic genomes.* Mobile DNA, 2015. **6**(1): p. 11.
30. Nene, V., et al., *Genome sequence of Aedes aegypti, a major arbovirus vector.* Science, 2007. **316**(5832): p. 1718-1723.
31. Xia, Q., et al., *A draft sequence for the genome of the domesticated silkworm (Bombyx mori).* Science, 2004. **306**(5703): p. 1937-1940.
32. Zhan, S., et al., *The monarch butterfly genome yields insights into long-distance migration.* Cell, 2011. **147**(5): p. 1171-1185.
33. Adams, M.D., et al., *The genome sequence of Drosophila melanogaster.* Science, 2000. **287**(5461): p. 2185-2195.
34. Consortium, H.G., *Butterfly genome reveals promiscuous exchange of mimicry adaptations among species.* Nature, 2012. **487**(7405): p. 94-98.
35. Ahola, V., et al., *The Glanville fritillary genome retains an ancient karyotype and reveals selective chromosomal fusions in Lepidoptera.* Nature communications, 2014. **5**.
36. Stanke, M., et al., *Using native and syntenically mapped cDNA alignments to improve de novo gene finding.* Bioinformatics, 2008. **24**(5): p. 637-644.
37. Blanco, E., G. Parra, and R. Guigó, *Using geneid to identify genes.* Current protocols in bioinformatics, 2007: p. 4.3. 1-4.3. 28.

38. Lomsadze, A., P.D. Burns, and M. Borodovsky, *Integration of mapped RNA-Seq reads into automatic training of eukaryotic gene finding algorithm.* Nucleic acids research, 2014: p. gku557.
39. Trapnell, C., L. Pachter, and S.L. Salzberg, *TopHat: discovering splice junctions with RNA-Seq.* Bioinformatics, 2009. **25**(9): p. 1105-1111.
40. Trapnell, C., et al., *Differential gene and transcript expression analysis of RNA-seq experiments with TopHat and Cufflinks.* Nature protocols, 2012. **7**(3): p. 562-578.
41. Campbell, M.A., et al., *Comprehensive analysis of alternative splicing in rice and comparative analyses with Arabidopsis.* BMC genomics, 2006. **7**(1): p. 327.
42. Haas, B.J., et al., *Automated eukaryotic gene structure annotation using EVidenceModeler and the Program to Assemble Spliced Alignments.* Genome biology, 2008. **9**(1): p. R7.
43. Consortium, U., *Reorganizing the protein space at the Universal Protein Resource (UniProt).* Nucleic acids research, 2011: p. gkr981.
44. Pruitt, K.D., T. Tatusova, and D.R. Maglott, *NCBI reference sequences (RefSeq): a curated non-redundant sequence database of genomes, transcripts and proteins.* Nucleic acids research, 2007. **35**(suppl 1): p. D61-D65.
45. Jones, P., et al., *InterProScan 5: genome-scale protein function classification.* Bioinformatics, 2014. **30**(9): p. 1236-1240.
46. Li, H. and R. Durbin, *Inference of human population history from individual whole-genome sequences.* Nature, 2011. **475**(7357): p. 493-496.
47. Li, H., et al., *The sequence alignment/map format and SAMtools.* Bioinformatics, 2009. **25**(16): p. 2078-2079.
48. Li, L., C.J. Stoeckert, and D.S. Roos, *OrthoMCL: identification of ortholog groups for eukaryotic genomes.* Genome research, 2003. **13**(9): p. 2178-2189.
49. Löytynoja, A. and N. Goldman, *An algorithm for progressive multiple alignment of sequences with insertions.* Proceedings of the National Academy of Sciences of the United States of America, 2005. **102**(30): p. 10557.
50. Castresana, J., *Selection of conserved blocks from multiple alignments for their use in phylogenetic analysis.* Molecular biology and evolution, 2000. **17**(4): p. 540-552.
51. Tamura, K., et al., *MEGA6: molecular evolutionary genetics analysis version 6.0.* Molecular biology and evolution, 2013: p. mst197.
52. Ronquist, F. and J.P. Huelsenbeck, *MrBayes 3: Bayesian phylogenetic inference under mixed models.* Bioinformatics, 2003. **19**(12): p. 1572-1574.
53. Posada, D., *Using MODELTEST and PAUP* to select a model of nucleotide substitution.* Current protocols in bioinformatics, 2003: p. 6.5. 1-6.5. 14.
54. De Bie, T., et al., *CAFE: a computational tool for the study of gene family evolution.* Bioinformatics, 2006. **22**(10): p. 1269-1271.
55. Bindea, G., et al., *ClueGO: a Cytoscape plug-in to decipher functionally grouped gene ontology and pathway annotation networks.* Bioinformatics, 2009. **25**(8): p. 1091-1093.

56. Sneddon, T.P., P. Li, and S.C. Edmunds, *GigaDB: announcing the GigaScience database.* GigaScience, 2012. **1**(1): p. 11.

Tables

Table 1. Summary statistics of generated whole genome shotgun sequencing data using Illumina Nextseq 500.

Library Name	Library Type	Insert Size	Platform	Read Length	No. Read	Total bp
350bp	Paired-end	350bp	Nextseq500	151	293,176,268	44,269,616,468
700bp	Paired-end	700bp	Nextseq500	151	246,945,900	37,288,830,900
3Kbp	Mate-pair	3Kbp	Nextseq500	76	284,204,762	21,599,561,912
6Kbp	Mate-pair	6Kbp	Nextseq500	76	246,238,370	18,714,116,120
9Kbp	Mate-pair	9Kbp	Nextseq500	76	239,919,538	18,233,884,888
**Total**						140,106,010,288

Table 2. Summary statistics for Illumina synthetic long read (Moleculo) library.

	500-1499bp	>= 1500bp
Number of assembled read	302,132	342,738
Number of bases in assembled read	268,853,717	1,205,349,082
N50 length of assembled read	960	4,031

Table 3. Summary statistics for generated long reads data using Pacbio RS II system.

Number of Reads	1,005,571
Total Bases	5,836,969,225
Length of longest (shortest) read	50,132(50)
Average read length	5,804.63

Table 4. Summary statistics of generated transcriptome data for six organ tissues using Illumina platform.

Tissue	Sample Name	Read Length	Read Count	Total Base (bp)
Hemocyte	Hemocyte_1	76	20,815,674	1,581,991,224
	Hemocyte_2	76	26,704,666	2,029,554,616
	Hemocyte_2	76	53,068,562	4,033,210,712
Malpighi	Malpighi_1	76	22,635,428	1,720,292,528
	Malpighi_2	76	24,893,788	1,891,927,888
	Malpighi_3	76	45,213,164	3,436,200,464
Midgut	Midgut_1	76	23,350,138	1,774,610,488
	Midgut_2	76	24,597,972	1,869,445,872
	Midgut_3	76	50,949,986	3,872,198,936
Head	Head_1	76	26,526,276	2,015,996,976
	Head_2	76	26,581,124	2,020,165,424
	Head_3	76	40,900,456	3,108,434,656
Skin	Skin_1	76	24,592,846	1,869,056,296
	Skin_2	76	42,775,430	3,250,932,680
	Skin_3	76	35,043,570	2,663,311,320
Fat Body	Fat Body_1	76	24,637,810	1,872,473,560
	Fat Body_2	76	24,037,494	1,826,849,544
	Fat Body_3	76	40,817,582	3,102,136,232
AM/Silk Gland	AM/Silk Gland_1	76	21,399,638	1,626,372,488
	AM/Silk Gland_2	76	24,292,386	1,846,221,336
	AM/Silk Gland_3	76	37,331,530	2,837,196,280
P/Silk Gland	P/Silk Gland_1	76	27,359,580	2,079,328,080
	P/Silk Gland_2	76	23,300,962	1,770,873,112
	P/Silk Gland_3	76	39,421,430	2,996,028,680
Testis	Testis_1	76	40,890,404	3,107,670,704
	Testis_2	76	45,733,846	3,475,772,296
	Testis_3	76	44,985,224	3,418,877,024
Ovary	Ovary_1	76	40,797,628	3,100,619,728
	Ovary_2	76	40,409,752	3,071,141,152
	Ovary_3	76	42,417,892	3,223,759,792

Table 5. Summary statistics for the *A. yamamai* genome (>2kb).

**Assembled Genome**	
Size(1n)	656 Mb
GC level	34.07
No. scaffolds	3,675
N50 of scaffolds (bp)	739,388
N bases in scaffolds (%)	19,257,439 (2.93)
Longest(shortest) scaffolds (bp)	3,156,949 (2,003)
Average scaffold Length (bp)	178,657.53

Table 6. Summary of identified repeat elements in the *A. yamamai* genome.

**Repeat Element**	No. Element	Length (%)
SINE	59,968	8,615,338(1.30)
LINE	426,522	101,251,176(15.31)
LTR element	53,977	4,552,386(0.69)
DNA element	512,760	69,071,227(10.44)
Small RNA	43,645	6,691,619(1.01)
Simple repeat	135,989	6,256,839(0.95)
Low complexity	19,937	932,829(0.14)
Unclassified	294,190	54,552,009(8.25)

Table 7. Summary statistics for ab initio, RNA-seq based and homology based gene prediction results.

Evidence Type	Programs	Element	Total count	Exon/Gene	Total length(bp)	Mean length(bp)
ab_initio	Augustus	Gene	14,576	4.85	142,415,318	9,770.53
		Exon	70,733		14,736,668	208.34
	Geneid	Gene	10,946	2.25	46,119,402	4,213.35
		Exon	24,686		3,925,563	159.01
	GeneMarks-ET	Gene	27,754	5.50	273,745,951	9,863.29
		Exon	152,660		30,847,503	202.06
RNA-seq	Cufflinks Transdecoder	Gene	36,213	7.03	840,429,061	23,207.94
		Exon	254,770		201,721,675	791.77
Known Gene (NCBI lepidop)	PASA (gmap)		44,561		22,484,151	504.57

Table 8. Summary statistics for the consensus gene set of *A. yamamai* genome.

Element	No. elements	Exon/Gene	Avg. length	Total length	Genome coverage
Gene	21,124	4.44	8,331.63	175,997,473	26.61
Exon	93,950		236.53	22,222,354	3.35

Figures

Figure 1. Photograph of *Antheraea Yamamai*. From left, larva, cocoon and adult *A. yamamai*, respectively. Specific green color is one representative characteristics of tensan silk.

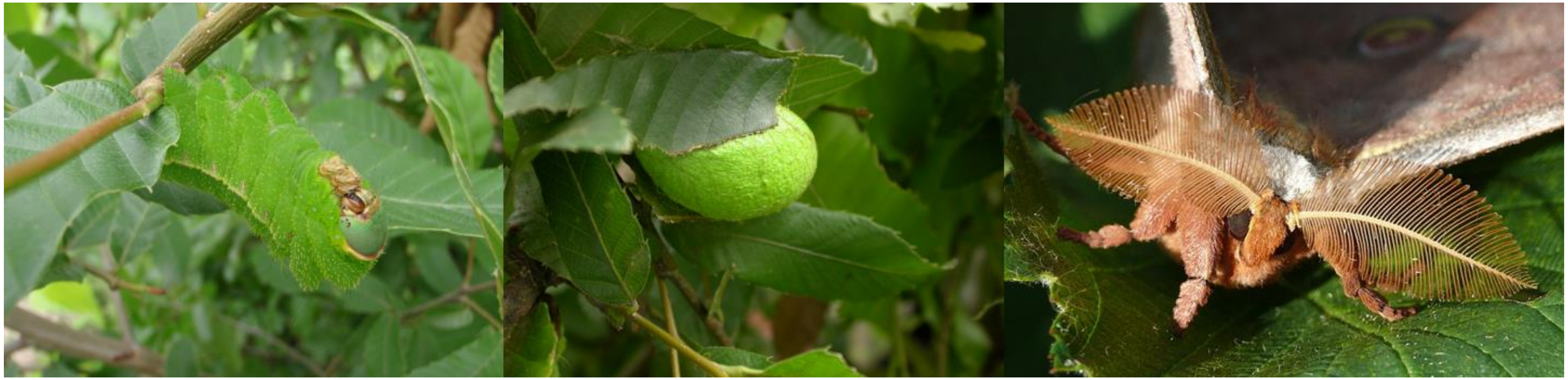

Figure 2. Karyotype of *A.yamamai* using a gamete of testis in metaphase.

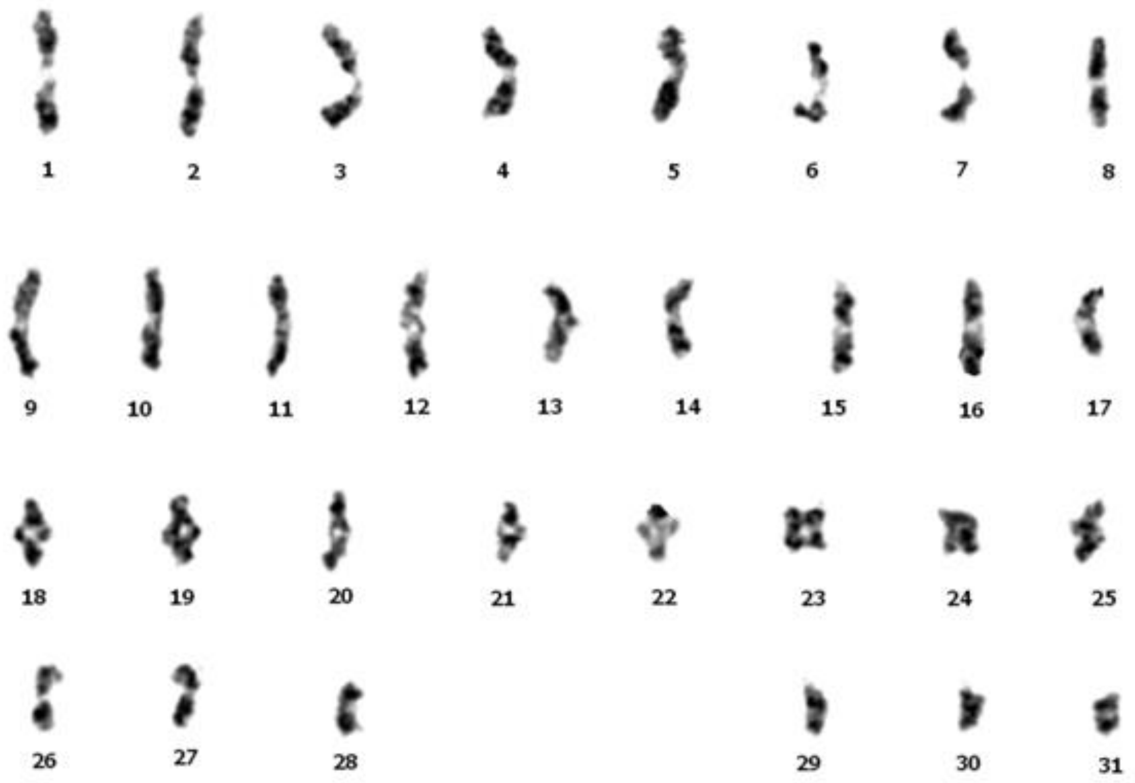

Figure 3. 19-mer distribution of *A. yamamai* genome using jellyfish with 350bp paired-end whole genome sequencing data.

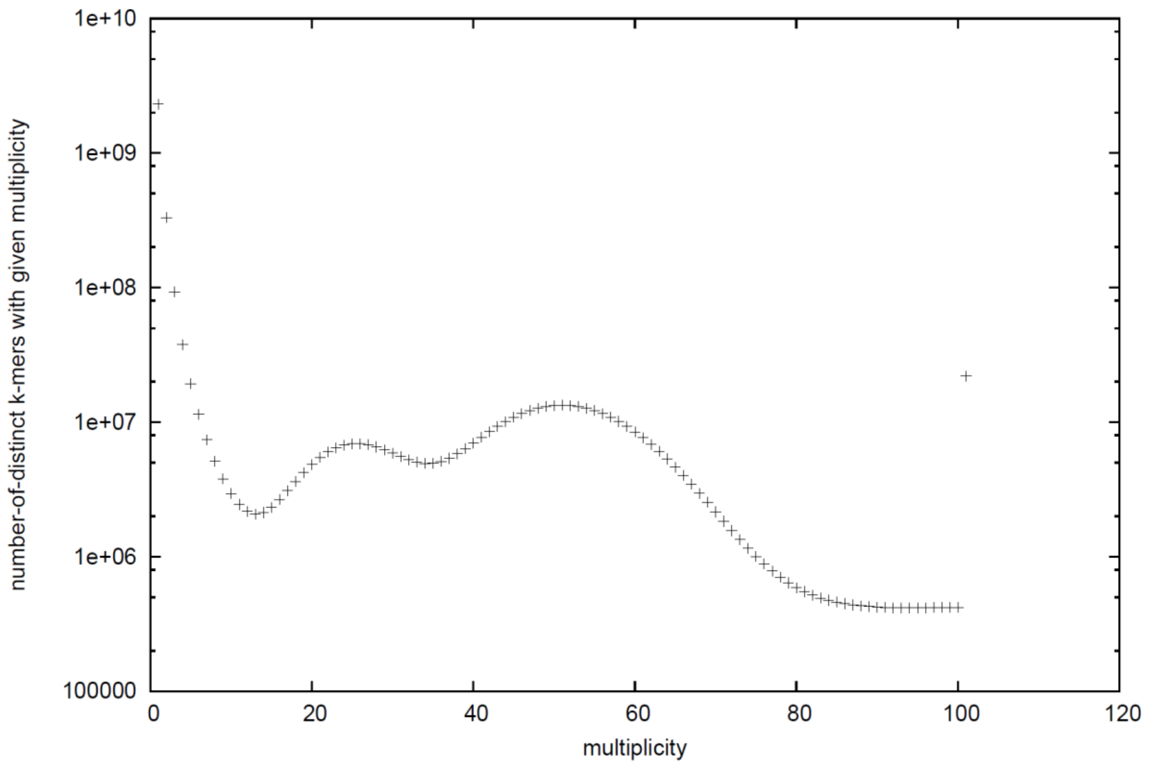

Figure 4. Amount and proportion of identified repeat element from 8 species including *A. yamamai*. a. Absolute amount of repeat element classified into 8 different categories. b. Proportion of each repeat element in identified total repeat element.

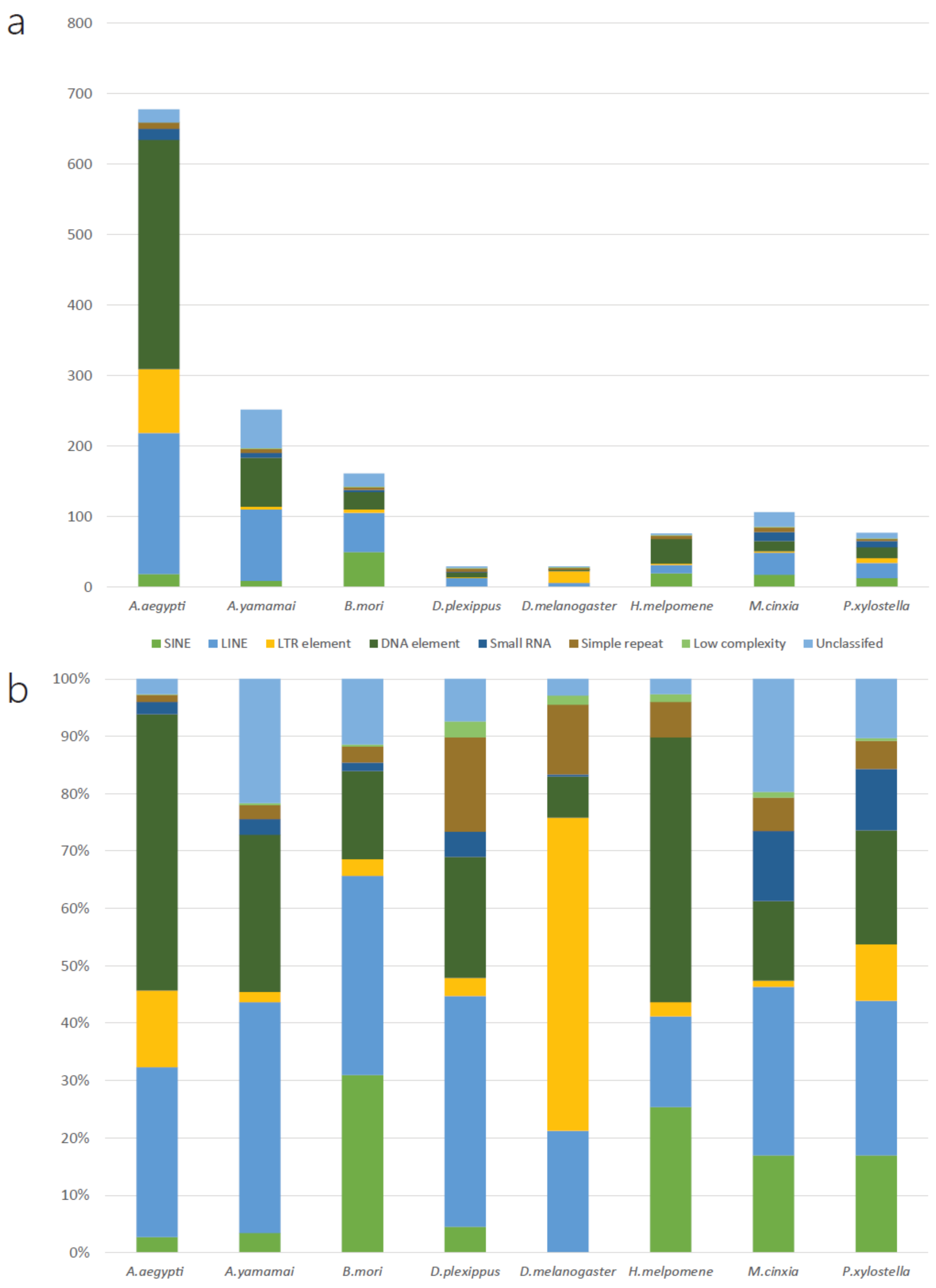

Figure 5. Demographic history of *A. yamamai* using PSMC and comparative gene family analysis. Node value indicate Bayesian posterior probability, bootstrap and gene expansion, contraction value. Orange and blue color indicate expansion and contraction, respectively.

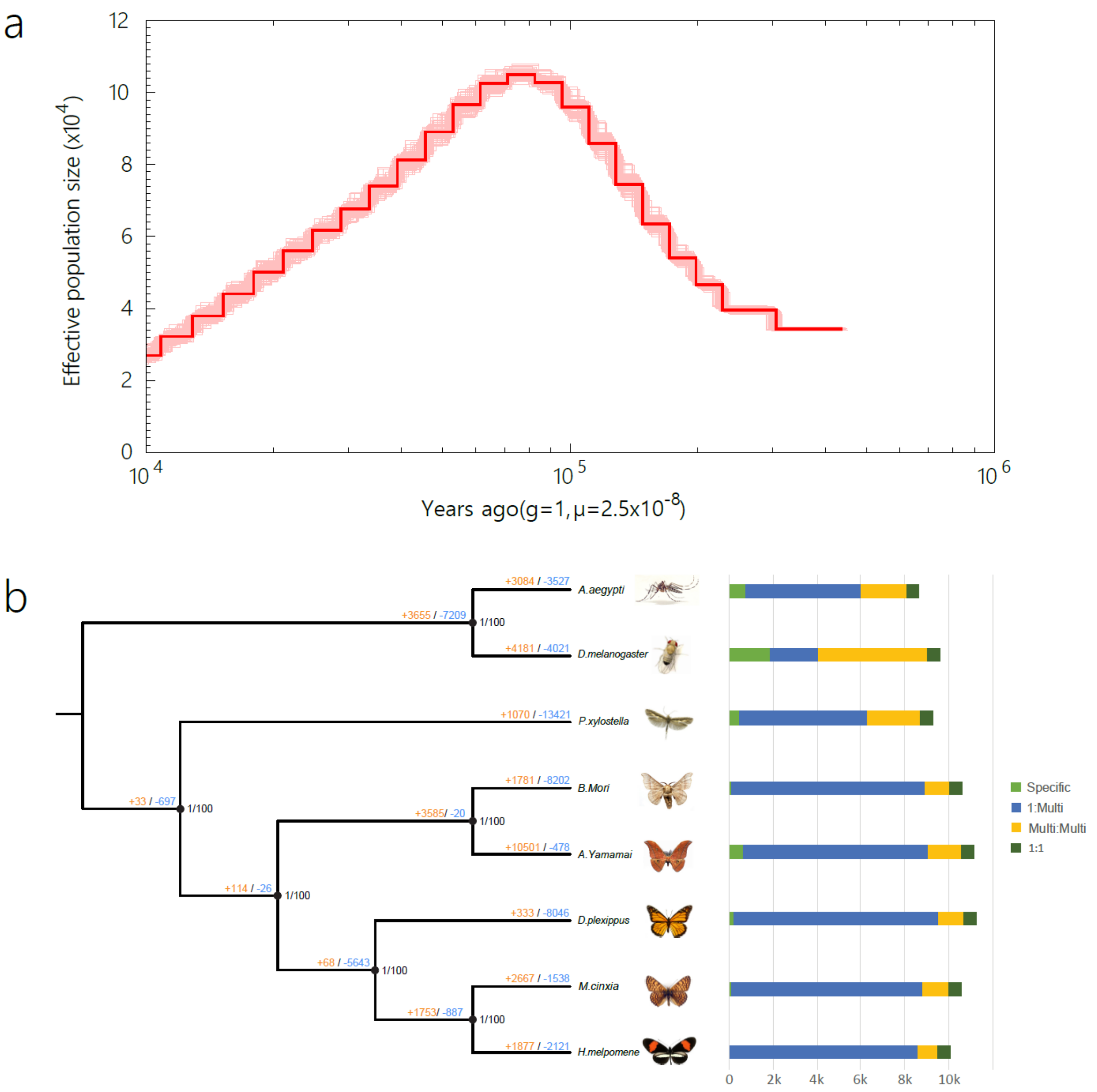

 Click here to download Figure Figure1.png

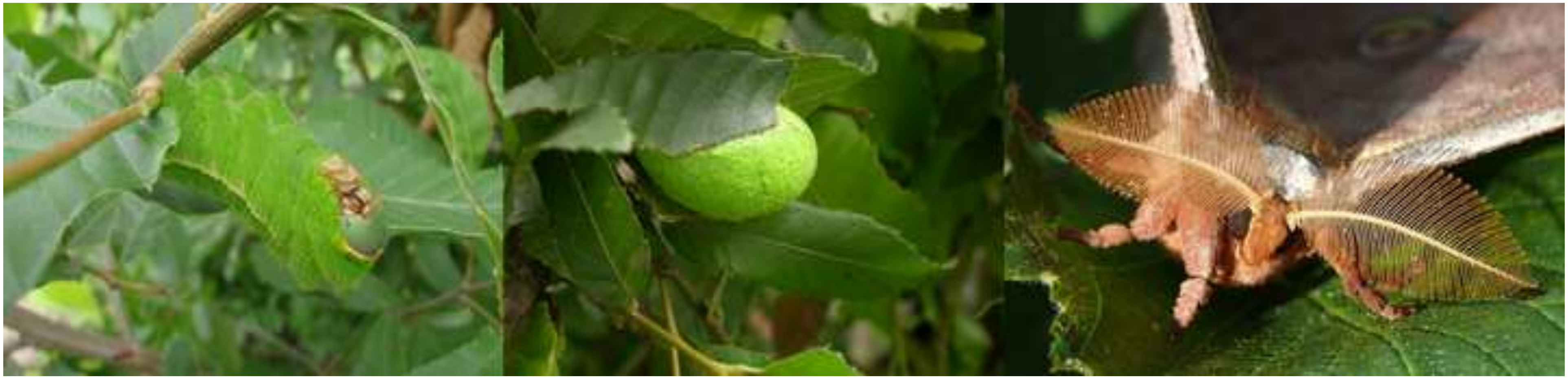

Click here to download Figure Figure2.jpg

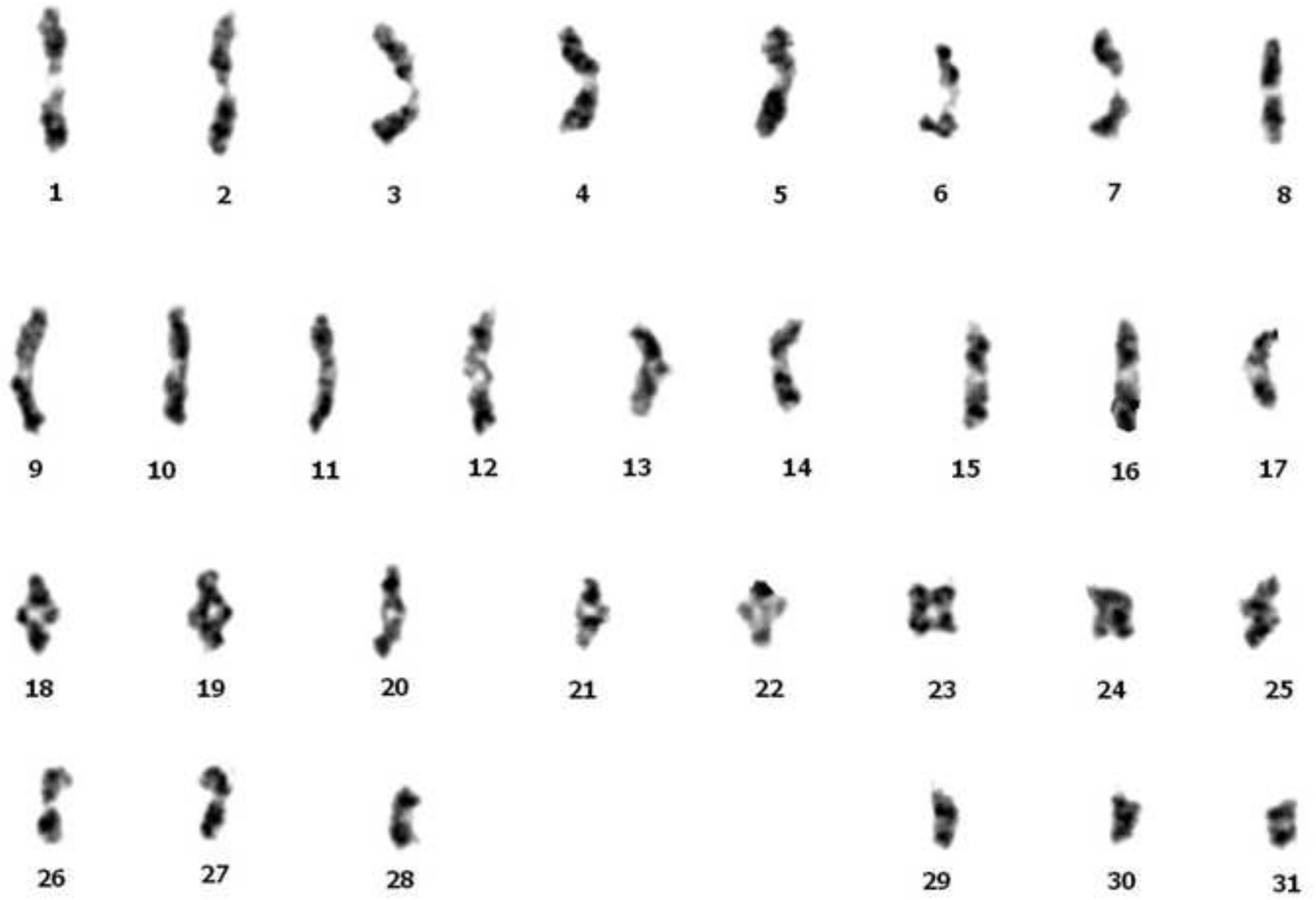

 Click here to download Figure Figure3.pdf

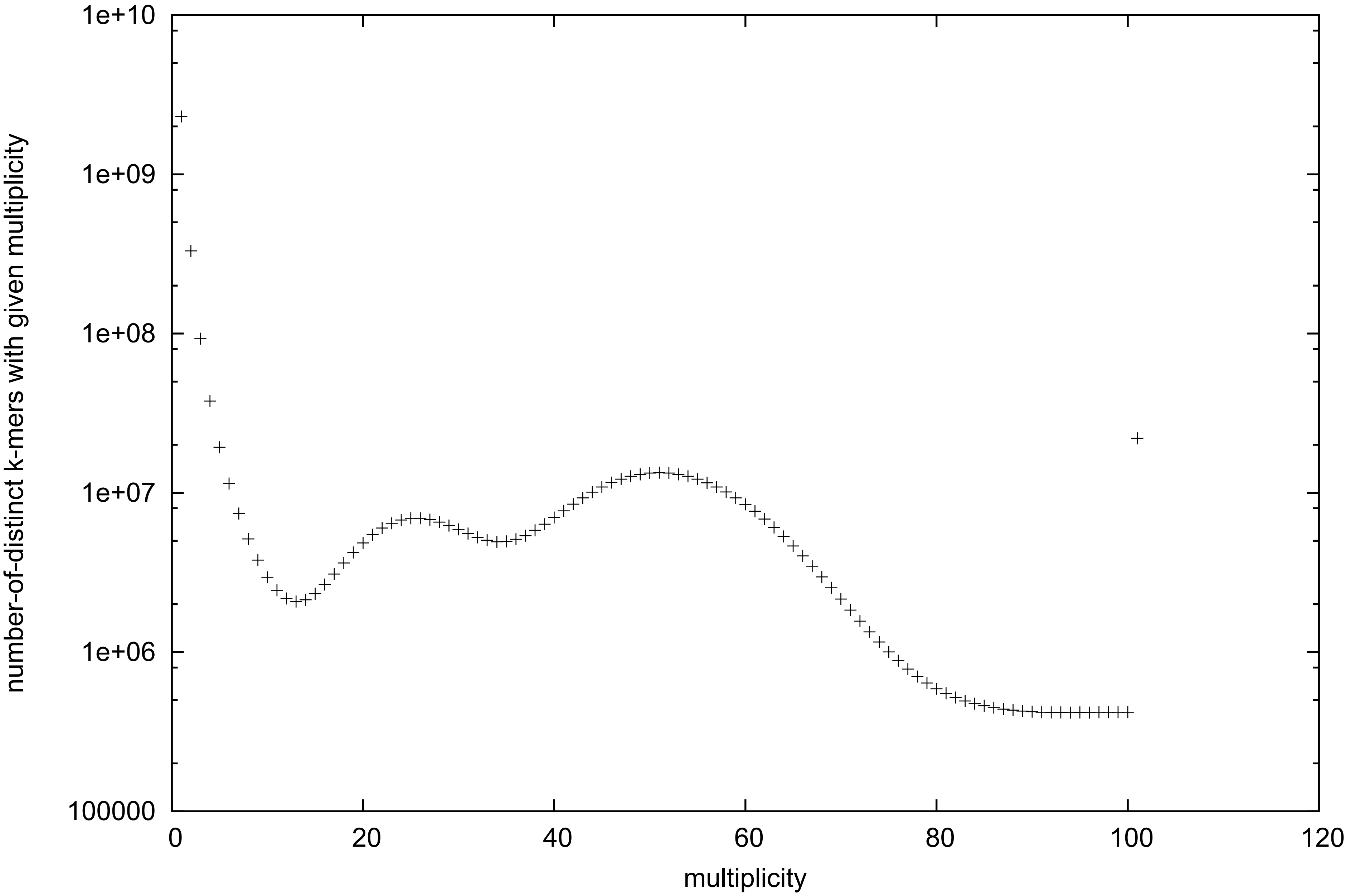

Figure

Click here to download Figure Figure4.pdf

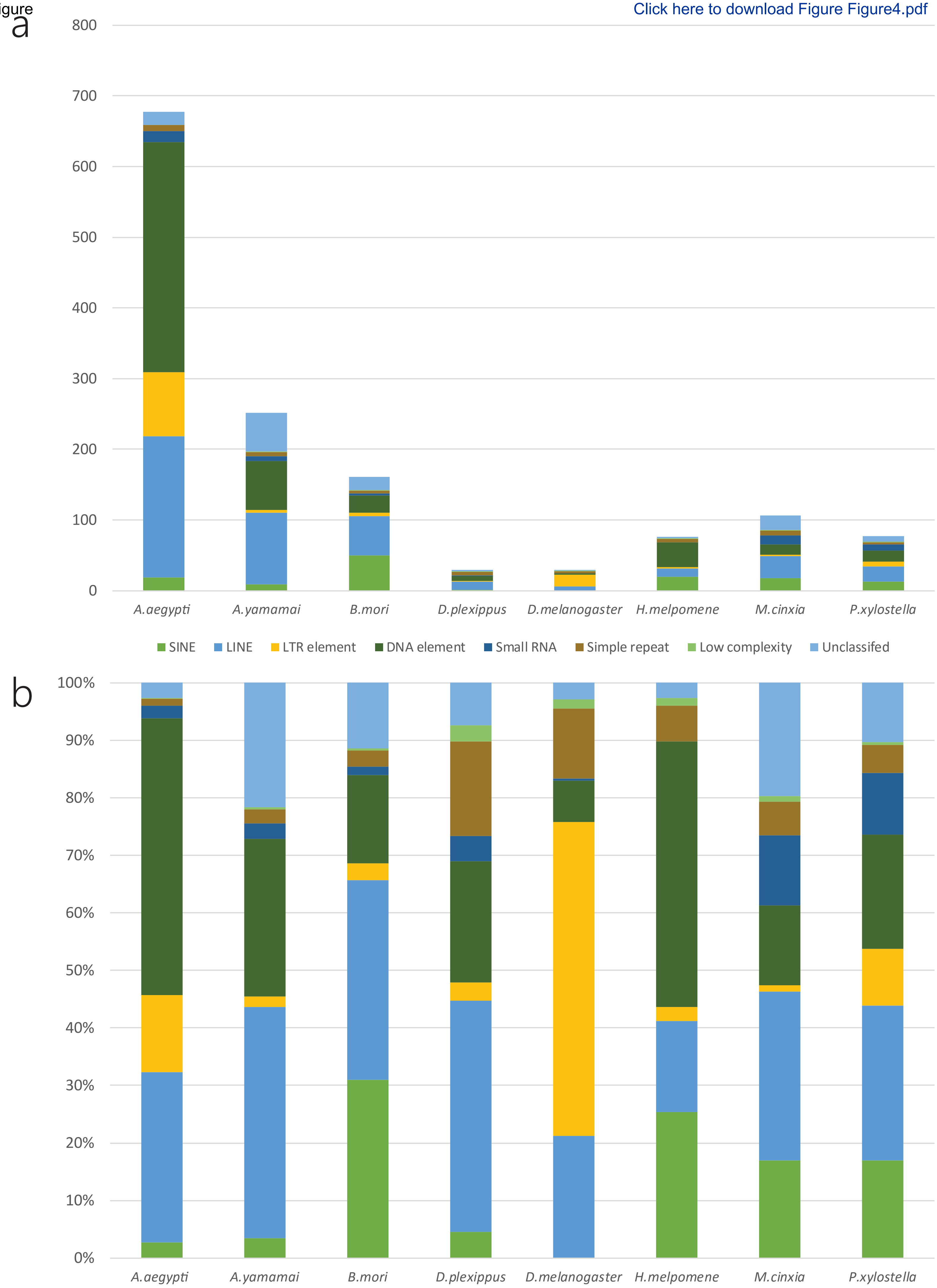

Click here to download Figure Figure5.pdf

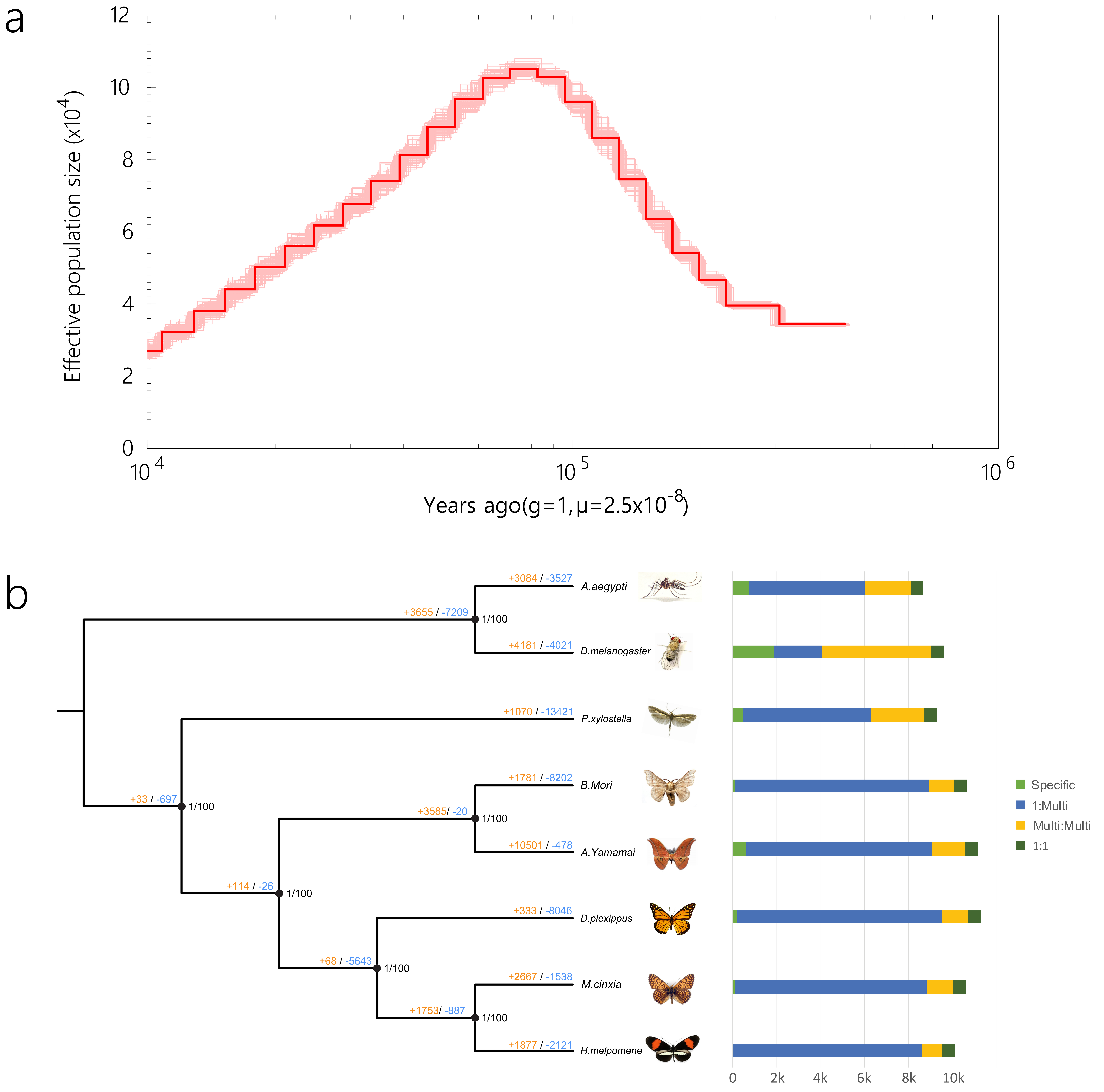

Click here to access/download
Supplementary Material
Supplementary_Information.docx

, April 18, 2017

Dear Editor of *Gigascience*,

I am pleased to submit our research article entitled “First wild silkworm genome of Japanese silk moth, *Antheraea yamamai*”, to your reputed journal, ***Gigascience***.

Unlike *Bombyx mori*, few studies have investigated the genomic information for the wild-type silkworm. Wild-type silkworms, *A. yamamai and A. perny*, are moth genus belonging to the family Saturniidae and which produce wild silk of commercial importance. In this article, we attempted to the whole-genome sequencing for the *A. yamamai*, thereby we constructed genome of *A. yamamai* were 656 Mb(>2kb) with 3,675 scaffolds and N50 length of assembly was 739 Kb with 34.07% GC ratio. To the best of our knowledge, these results will provide valuable genomic information for understanding the molecular mechanisms related to the specific phenotypes such as wild silk itself, and more insight into Saturniidae evolution process.

The material is original research, has not been previously published and has not been submitted for publication elsewhere while under consideration. The authors have declared that they have no conflict of interest.

I hope this paper can meet your approval and can be published at the earliest possible date.

Looking forward to hearing from you again.
Thank you.

With best regards,

Prof. Seung-Won Park
Department of Biotechnology,
Catholic University of Daegu, Gyeongsan-si, Gyeongsangbuk-do 38430, Republic of Korea,
Tel: +82-53-850-3176, E-mail: microsw@cu.ac.kr
