# Supplementary material for: Genome sequence of the Japanese oak silk moth, Antheraea yamamai: the first draft genome in the family Saturniidae
Source: Gigascience. 2017 Nov 27;7(1):gix113. doi: 10.1093/gigascience/gix113 (PMC5774507; doi:10.1093/gigascience/gix113)
Supplement: GIGA-D-17-00085_Revision-1.pdf [file gix113_giga-d-17-00085_revision-1.pdf]

GigaScience

Genome sequence of Japanese oak silk moth, Antheraea yamamai: the first draft genome in family Saturniidae

--Manuscript Draft--

Manuscript Number:	GIGA-D-17-00085R1
Full Title:	Genome sequence of Japanese oak silk moth, Antheraea yamamai: the first draft genome in family Saturniidae
Article Type:	Data Note
Funding Information:	Rural Development Administration (PJ010442) — Dr Seong-Ryul Kim
Abstract:	Background<br>Antheraea yamamai, also known as the Japanese oak silk moth, is a wild species of silk moth. Silk produced by A. yamamai, referred to as tensan silk, differs drastically from common silk produced from the domesticated silkworm, Bombyx mori. Its unique characteristics have led to its use in many research fields including biotechnology and medical science, and the scientific as well as economic importance of wild silk moth continues to gradually increase. However, no genomic information for wild silk moth, including A. yamamai, is currently available.<br><br>Findings<br>In order to construct the A. yamamai genome, a total of 147G base pairs using Illumina and Pacbio sequencing platforms were generated, providing 210-fold coverage based on the 700 Mb estimated genome size of A. yamamai. The assembled genome of A. yamamai was 656 Mb(>2kb) with 3,675 scaffolds and the N50 length of assembly was 739 Kb with 34.07% GC ratio. Identified repeat elements covered 37.33% of the total genome and the completeness of the constructed genome assembly was estimated to be 96.7% by BUSCO v2 analysis. A total of 21,124 genes were identified using Evidence Modeler based on the gene prediction results obtained from 3 different methods (ab initio, RNA-seq based, known-gene based).<br><br>Conclusions<br>Here we present the genome sequence of A. yamamai, the first genome sequence of wild silk moth. These results provide valuable genomic information which will help enrich our understanding of the molecular mechanisms related to not only specific phenotypes such as wild silk itself, but also the genomic evolution of Saturniidae.
Corresponding Author:	Seung-Won Park<br><br>KOREA, REPUBLIC OF
Corresponding Author Secondary Information:	
Corresponding Author's Institution:	
Corresponding Author's Secondary Institution:	
First Author:	Seong-Ryul Kim
First Author Secondary Information:	
Order of Authors:	Seong-Ryul Kim
	Woori Kwak
	Kelsey Caetano-Anolles
	Kee-Young Kim
	Su-Bae Kim
	Kwang-Ho Choi

Powered by Editorial Manager® and ProduXion Manager® from Aries Systems Corporation

	Seong-Wan Kim
	Jae-Sam Hwang
	Min-Jee Kim
	Iksoo Kim
	Tae-Won Goo
	Seung-Won Park
**Order of Authors Secondary Information:**	
**Response to Reviewers:**	Dear Editor,<br><br>We corrected our manuscript followed the editor's comment.<br><br>The response and details of revision can be found in memo section.<br><br>We hope our revised manuscript can meet the quality standard of the editor and future peer reviewers.<br><br>If you need anything, just let us know.<br><br>On behalf of all authors,<br>Seung-Won Park
**Additional Information:**	
**Question**	**Response**
Are you submitting this manuscript to a special series or article collection?	No
**Experimental design and statistics**<br><br>Full details of the experimental design and statistical methods used should be given in the Methods section, as detailed in our Minimum Standards Reporting Checklist. Information essential to interpreting the data presented should be made available in the figure legends.<br><br>Have you included all the information requested in your manuscript?	Yes
**Resources**<br><br>A description of all resources used, including antibodies, cell lines, animals and software tools, with enough information to allow them to be uniquely identified, should be included in the Methods section. Authors are strongly encouraged to cite Research Resource Identifiers (RRIDs) for antibodies, model organisms and tools, where possible.<br><br>Have you included the information requested as detailed in our Minimum	Yes

Standards Reporting Checklist?	
**Availability of data and materials**<br><br>All datasets and code on which the conclusions of the paper rely must be either included in your submission or deposited in publicly available repositories (where available and ethically appropriate), referencing such data using a unique identifier in the references and in the “Availability of Data and Materials” section of your manuscript.<br><br>Have you have met the above requirement as detailed in our Minimum Standards Reporting Checklist?	Yes

Powered by Editorial Manager® and ProduXion Manager® from Aries Systems Corporation

Manuscript

Click here to download Manuscript MothManuscript_RevMo_noTT.docx

Genome sequence of Japanese oak silk moth, *Antheraea yamamai*: the first draft genome in family Saturniidae

Seong-Ryul Kim[1†], Woori Kwak[2†], Kelsey Caetano-Anolles[3], Kee-Young Kim[1], Su-Bae Kim[1], Kwang-Ho Choi[1], Seong-Wan Kim[1], Jae-Sam Hwang[1], Min-Jee Kim[4], Iksoo Kim[4], Tae-Won Goo[5] and Seung-Won Park[6]*

[1]Department of Agricultural Biology, National Academy of Agricultural Science, Rural Development Administration, Wanju-gun 55365, Republic of Korea; [2]C&K Genomics, Main Bldg. #420, SNU Research Park, Seoul 151-919, Republic of Korea; [3]Department of Agricultural Biotechnology and Research Institute of Agriculture and Life Sciences, Seoul National University, Seoul 151-921, Republic of Korea; [4]College of Agriculture & Life Sciences, Chonnam National University, Gwangju, Republic of Korea; [5]Department of Biochemistry, Dongguk University College of Medicine, Gyeongju-si, Gyeongsangbuk-do 38066, Republic of Korea; [6]Department of Biotechnology, Catholic University of Daegu, Gyeongsan-si, Gyeongsangbuk-do 38430, Republic of Korea

Seong-Ryul Kim : ksr319@korea.kr; Woori Kwak : asleo@cnkgenomics.com; Kelsey Caetano-Anolles : kelseyca@gmail.com; Kee-Young Kim : applekky@korea.kr; Su-Bae Kim : subae@korea.kr; Kwang-Ho Choi : ckh@korea.kr; Seong-Wan; Seong-Wan Kim : tarupa@korea.kr; Jae-Sam Hwang : hwangjs@korea.kr; Min-Jae Kim : minjeekim3@gmail.com; Iksoo Kim : ikkim81@chonnam.ac.kr; Tae-Won Goo : gootw@dongguk.ac.kr

[†] These authors equally contributed and should be regarded as co-first authors.

* Corresponding authors

Seung-Won Park

Department of Biotechnology,

Catholic University of Daegu,

Gyeongsan-si, Gyeongsangbuk-do 38430, Republic of Korea

Phone : +82-53-850-3176

Fax : +82-53-359-6846

E-mail: microsw@cu.ac.kr

Abstract

Background

Antheraea yamamai, also known as the Japanese oak silk moth, is a wild species of silk moth. Silk produced by *A. yamamai*, referred to as *tensan* silk, differs drastically from common silk produced from the domesticated silkworm, *Bombyx mori*. Its unique characteristics have led to its use in many research fields including biotechnology and medical science, and the scientific as well as economic importance of wild silk moth continues to gradually increase. However, no genomic information for wild silk moth, including *A. yamamai*, is currently available.

Findings

In order to construct the *A. yamamai* genome, a total of 147G base pairs using Illumina and Pacbio sequencing platforms were generated, providing 210-fold coverage based on the 700 Mb estimated genome size of *A. yamamai*. The assembled genome of *A. yamamai* was 656 Mb(>2kb) with 3,675 scaffolds and the N50 length of assembly was 739 Kb with 34.07% GC ratio. Identified repeat elements covered 37.33% of the total genome and the completeness of the constructed genome assembly was estimated to be 96.7% by BUSCO v2 analysis. A total of 21,124 genes were identified using Evidence Modeler based on the gene prediction results obtained from 3 different methods (*ab initio*, RNA-seq based, known-gene based).

Conclusions

Here we present the genome sequence of *A. yamamai*, the first genome sequence of wild silk moth. These results provide valuable genomic information which will help enrich our understanding of the molecular mechanisms related to not only specific phenotypes such as wild silk itself, but also the genomic evolution of Saturniidae.

Keywords

Antheraea yamamai, Japanese silk moth, Japanese oak silk moth, wild silkworm

Data description

Antheraea yamamai (Figure 1), also known as the Japanese oak silk moth, is a wild silk moth species belonging to the Saturniidae family. Silk moths can be categorized into two families- Bombycidae and Saturniidae. Saterniidae has been estimated to contain approximately 1,861 species with 162 genera[1] and is known as the largest family in Lepidoptera. Among the many species in family Saturniidae, only a few species, including *A. yamamai,* can be utilized for silk production. Previous phylogenetic studies have showed that family Saturniidae shares common ancestors with family Sphingidae, including the hawk moth (*Macroglossum stellatarum*), the and Bombycidae family, including the most representative silkworm, *Bombyx mori* [2]. Divergence time of *A. yamamai* from *B. mori* was estimated to be 87 MYA(million years ago) and *A. yamamai* is evolutionary further away from *B.mori* compared to *B. mandariana* (0.0041 MYA), which is as wild type species of *B.mori*[3, 4].

The most unique species-specific phenotypic trait of *A. yamamai* is their silk itself, which is known as tensan silk[5]. This silk shows distinctive characteristics such as thickness, bulkiness, compressive elasticity, and resistance to chemicals compared to common silk from *Bombyx mori*[6-8]. Therefore, it has attracted the attention of researchers as a new biomaterial for use in various fields[9-11]. Additionally, peptides from *A. yamamai* have been studied for their applications for human health[12-15]. However, despite the importance of wild silk moth in research and economic fields, no genomic information is currently available for this wild silk moth or any other species from family Saturniidae.

In this study, we present the genome sequence of *A. yamamai*, the first published genome in family Saturniidae, with gene expression data collected from ten different body organ tissues. This data will be a fundamental resource for future studies and provide more insight into the genome evolution and molecular phylogeny of family Saturniidae .

Sequencing

For whole genome sequencing, we selected one male sample(Ay-7-male1) from a breeding line (Ay-7) of *A. yamamai* raised at the National Academy of Agricultural Science, Rural Development Administration, Korea. Before conducting sequencing analysis, we conducted karyotyping analysis in order to confirm the number of chromosomes and chromosome abnormalities using a gamete in metaphase. Figure 2 shows the result of karyotyping analysis on the genome of the Japanese oak silk moth, which consists of 31 chromosomes. For genomic and transcriptomic library construction, we removed the guts of *A. yamamai* to prevent contamination of genomes from other organisms such as gut microbes and oak, the main food source of *A. yamamai*. Details of the sample preparation process used in this study are presented in supplementary information. Genomic DNA was extracted using a DNeasy Animal Mini Kit (Qiagen, Hilden, Germany) and the quality of extracted DNA was checked using trenean, picogreen assay and gel electrophoresis (1% agarose gel/ 40ng loading). After quality control processing, we were left with a total of 61.5ug of *A. yamamai* DNA for genome sequencing. Using standard Illumina whole genome shotgun(WGS) sequencing protocol (paired-end and mate-pair), we added two long read sequencing platforms, Moleculo (Illumina synthetic long read) and RS II(Pacific Bioscience). Table 1-3 shows a summary of generated data for each library used in this study. RNA-seq libraries were also constructed for genome annotation and specific gene expression of 10 different tissues (Hemocyte, Malpighi, Midgut, Fat Body, AM/Silk gland, P/Silk gland, Head, Skin, Testis, Ovary) with 3 biological replicates following standard manufacturer protocol (Illumina, San Diego, CA, USA). For this, more than 100 individual *A. yamamai* samples from the same breeding line were used for tissue anatomy and 3 samples from each tissue were selected based on the quality of extracted RNA. Information of libraries and generated data is provided in Table 4. A total of

147Gb of genomic data and 76Gb of transcriptomic data was generated for this study.

Genome assembly and evaluation

Before conducting genome assembly, we conducted k-mer distribution analysis using a 350bp paired-end library in order to estimate the size and characteristics of the *A. yamamai* genome. The quality of our generated raw data was checked using FASTQC[16](FastQC , RRID:SCR_014583). Sequencing artifacts such as adapter sequences and low quality bases were removed using Trimmomatic[17]. Jellyfish[18] was used to count the k-mer frequency for estimation of the genome size of *A. yamamai*. Figure 3 shows the 19-mer distribution of *A. yamamai* genome using a 350bp paired-end library. In the 19-mer distribution, there was a second peak in the half x-axis of the main peak which indicates heterozygosity. Although the inbred line used in this study was maintained for more than 10 generations, high heterozygosity still remains. This phenomenon has been observed in a previous genomic study of the black diamond moth (*Plutella xylostella*), and sustained heterozygosity as an important genomic characteristic was hypothesized to be a result of environmental adaption[19]. Based on the result of 19-mer distribution analysis, the genome size of *A. yamamai* was estimated to be 709Mb. Next, we conducted error correction on Illumina paired-end libraries using the error correction module of Allpaths-LG[20] before the initial contig assembly process (ALLPATHS-LG , RRID:SCR_010742). After error correction, initial contig assembly with 350bp and 700bp libraries was conducted using SOAP denovo2[21] with the parameter option set at K=19; this approach showed the best assembly statistics compared to other assemblers and parameters (SOAPdenovo2 , RRID:SCR_014986). Quality control processing for mate-pair libraries and scaffolding was conducted using Nxtrim[22] and SSPACE (SSPACE , RRID:SCR_011848)[23], respectively.

At each scaffolding step, SOAP Gapcloser[21] with -l 155 and -p 31 parameters was repeatedly used to close the gaps within each scaffold. In order to obtain a higher quality genome assembly of *A. yamamai*, we employed several long read scaffolding strategies using SSPACE-LongRead[24]. First, we used a Illumina synthetic long read sequencing platform called Moleculo which has been proven valuable for study of highly heterozygous genomes in previous study[25, 26]. After scaffolding was performed using SSPACE-LongRead with Illumina synthetic long read data, the total number of assembled scaffolds was effectively reduced from 398,446 to 24,558. The average scaffold length was also extended from 1.7 Kb to 24.8 Kb. However, there was no impressive improvement in N50 length (approximately 91 Kb to 112 Kb) of assembled scaffolds. Therefore, we employed another type of long read data generated from 10 cells of Pacbio RS II system with P6-C4 chemistry. After final scaffolding processing using Pacbio long reads, the number of scaffolds was reduced to 3,675 and N50 length was effectively extended from 112 Kb to 739 Kb. Summary statistics of the assembled *A. yamamai* genome is provided in Table 5. Final assembly of the *A. yamamai* genome was 656 Mb(>2kb) long with 3,675 scaffolds and the N50 length of assembly was 739 Kb with a 34.07% GC ratio. To evaluate the quality of the assembled genome, we conducted BUSCO (Benchmarking Universal Single-Copy Orthologs) analysis[27] using BUSCO v2.0 with insecta_odb9 including 1,658 BUSCOs from 42 species (BUSCO , RRID:SCR_015008). From BUSCO analysis, 96.7% of BUSCOs were completely detected in the assembled genome (1,576 : complete and single-copy, 27 : complete and duplicated) among 1,658 tested BUSCOs. The number of fragmented and missing BUSCOs was 21 and 34, respectively. Based on the result of BUSCO analysis, the genome of *A.yamamai* presented here was considered properly constructed for downstream analysis.

Repeat identification and comparative repeat analysis

To identify repeat elements of the *A. yamamai* genome, a custom repeat library was constructed using RepeatModeler with RECON[28], RepeatScout[29] and TRF[30]. The resulting constructed custom repeat library for *A. yamamai* was further curated using CENSOR[31] search and curated library was employed in RepeatMasker[32] with Repbase[33]. RepeatMasker was conducted with RMBlast and 'no_is' option. Table 6 summarizes the proportion of identified mobile elements in the A. *yamamai* genome. The most prevalent repeat element in the *A. yamamai* genome was LINE element (101 Mb, 15.31% of total genome) and total repeat elements accounted for 37.33% of the total genome. In order to compare the repeat elements of *A. yamamai* with that of other genomes, we conducted same process for seven public genomes which are close neighbors of *A. yamamai* - *Aedes aegypti[34]*, *Bombyx mori*[35], *Danaus plexippus*[36], *Drosophila melanogaster[37]*, *Heliconius melpomene*[38], *Melitaea cinxia*[39] and *Plutella xylostella*[19]. Figure 4 displays the amount and proportion of identified repeat elements from the 8 species. Despite the small genome size of *B. mori,* the total amount of identified SINE element in the *B. mori* genome was 5.77 times larger than that of *A. yamamai*. The top 5 expanded repeat elements in *A. yamamai* genome were DNA/RC, LINE/L2, LINE/RTE-BovB, DNA/TcMar-Mariner and LINE/CR1. Among these, DNA/TcMar-Mariner was the specifically expanded repeat element in *A. yamamai* among 8 species. In *B. mori*, SINE/tRNA-CR1, LINE/Jockey, DNA/RC, LINE/CR1-Zenon and LINE/RTE-BovB were the top 5 expanded repeat elements. When comparing the repeat elements of *A. yamamai* with those of *B. mori*, which are both producers of the same type of silk, repeat elements showed family and species specific patterns in the two silk moth linages. This indicates that there are differences in the genome evolution process between Saturniidae and Bombycidae families.

Gene prediction and annotation

Three different algorithms were used for gene prediction of the *A. yamamai* genome: *ab initio*, RNA-seq transcript based, and protein homology-based approaches. For *ab initio* gene prediction, Augustus[40], Geneid[41] and GeneMarks-ET[42] were employed. Augustus was trained using known genes of *A. yamamai* in NCBI database and mapping information of RNA-seq data obtained from Tophat[43](TopHat , RRID:SCR_013035) was also utilized for gene prediction. Geneid was used with predefined parameters for *Drosophila melanogaster*. GeneMarks-ET was employed using junction information of genes from transcriptome data alignment. For RNA-seq transcript based prediction, generated transcriptome data from ten organ tissues of *A. yamamai* were aligned to the assembled genome and gene information was predicted using Cufflinks[44](Cufflinks , RRID:SCR_014597). The longest CDS sequences were identified from Cufflinks results using Transdecoder. For the homology-based approach, all known genes of order Lepidoptera in the NCBI database were aligned using PASA[45]. Table 7 shows the gene prediction results from each method. Gene prediction results from different prediction algorithms were combined using EVM (Evidence Modeler)[46] and a consensus gene set of the *A. yamamai* genome was created. The final gene set of *A. yamamai* genome contains 21,124 genes. Summary statistics for the consensus gene set is provided in Table 8. The average gene length was 8,331 bp with a 38.76% GC ratio and the number of exons per gene was 4.44. To identify the function of predicted genes, Swiss-Prot[47], Uniref100[47], NCBI NR[48] database, and gene information of *B. mori* and *D. melanogaster* was employed for sequence similarity search using blastp. Additionally, protein domain search was conducted on the consensus gene set using InterproScan5[49]. Figure S1 shows the top 20 identified terms from 10 different InterproScan5 analyses. Among

the various analysis conducted using InterproScan5, gene ontology analysis showed that a large proportion of genes in the *A.yamamai* genome were related with molecular binding, catalytic activity, internal component of membrane, metabolic process, oxidation-reduction process and transmembrane transport.

Demographic history and comparative genome analysis

We estimated the demographic history of *A. yamamai* using the PSMC (Pairwise Sequentially Markovian Coalescent) method[50]. This method can infer the history of population size from a diploid sequence. 350bp paired-end reads were realigned to the assembled genome using Bowtie2, and consensus sequence data was generated from read alignment data using samtools[51] with parameters -d 10, -d 100. Bootstrap sampling was also executed 100 times and the generation time was set to 1 years based on the life cycle of *A. yamamai*. Figure 5a shows the inferred demographic history of *A. yamamai* using the PSMC model. Based on PSMC analysis, results suggest that the population size of *A. yamamai* species consistently increased before the last glacial period (approximately 110,000 to 12,000 years ago), which is similar to most other insect populations. During the last glacial period, the population size then continuously decreased. During the Late Glacial Maximum Period (13,000 to 10,000 years ago), which is also known as the beginning of the Modern Warm Period, the population size of *A.yamamai* did not increase and stayed at a low level.

We used OrthoMCL[52] and RBH(Reciprocal Best Hit) within blastp for identification of gene family clusters and 1:1 orthologous gene sets. A total of 18,013 gene family clusters were constructed and 3,586 1:1 orthologous genes were identified. Before conducting comparative genome analysis, we constructed phylogenetic trees for the 8 species. In order to

build the phylogenetic tree, multiple sequence alignment for the 1:1 orthologous genes of all 8 species was conducted using PRANK[53], and Gblocks[54] was used to obtain conserved blocks for the phylogenetic tree. Conserved block sequences were sequentially concatenated to obtain one consensus sequence for each species. MEGA6[55] was used for constructing Neighbor-Joining Trees (bootstrap 1000, maximum composite likelihood, transitions + transversions, and gamma distributed option) and MrBayes[56] was employed for construction of Bayesian inference trees. To select the best evolution model for our data, Modeltest[57] was conducted and the GTR based invariant model was chosen based on the AIC value of Modeltest. Figure 5b shows the constructed phylogenetic tree of the 8 species using 3,586 orthologous genes. The bootstrap value and Bayesian poster probability value of all nodes was 100 and 1, respectively. The closest neighbor of *A. yamamai* was *B. mori*, which is included in Bombycidae family; this result is consistent with that of previous studies. Three butterfly species (*D.plexippus*, *M.cinxia and H. meplmene*) included in Nymphalidae family were also shown to share a common ancestor with families Saturniidae and Bombycidae.

Based on the constructed phylogenetic tree, gene family expansion and contraction analysis was conducted using a 2 parameter model in CAFE[58]. Figure 5b shows the result of gene family expansion and contraction analysis of 8 species. 10,501 and 478 gene families were estimated to be expanded and contracted from the common ancestors of *A. yamamai*, respectively. In *B.mori*, 1,781 and 8,202 gene families were estimated for expansion and contraction, respectively. The number of expended and contracted genes in the genomes of *A. yamamai* and *B. mori* indicates that there are large differences in the genome evolution processes between the two silk moth species. *A. yamamai*'s genome also shows more specific gene family clusters when compared to the 7 other species, including *B.mori*. To identify the related function of specific gene family clusters expanded in the *A. yamamai* genome, gene

ontology pathway analysis was conducted using gene annotation information from *D. melanogaster* (E-value < 1E-9) using DAVID[59]. Figure S2 shows enriched terms of biological processes for specifically expanded gene family clusters resulting from gene ontology analysis. Specific gene clusters including UGT (UDP-glycosyltransferase) genes were expanded in *A. yamamai* genome and were related with the function of glucose import, hexose transmembrane transport, transmembrane transport, flavonoid glucuronidation, flavonoid biosynthetic process, oxidation-reduction process and regulation of chromatic silencing and transcription from RNA II promoter. Among these, flavonoid glucuronidation, flavonoid biosynthetic process, and functions of transmembrane transport are closely related to the creation of silk as well as diet. It has been shown previously that prepupae of silk moth are highly sensitive to UV-B irradiation and exposure to UV-B can dramatically decrease the pupation rates[60]. Therefore, a silk moth's cocoon is serves as a shield for protecting the moth from UV-B containing solar radiation during the larval-pupal transformation [60] and the degree of green pigmentation was determined by the intensity of irradiation[61]. Carotenoid[62] and flavonoid[63] are pigments which are crucial for determination of cocoon color and both pigments are derived from diet. Especially, the green cocoon color of *A. yamamai* has been shown to be the product of flavonoids[64]. Flavonoid is known to have a complex metabolic process which depends more heavily on diet than carotenoids [63] and previous research has shown that the green cocoon of certain silk moth contains more than 30 kinds of flavonoids which are not present in diet[65]. Therefore, flavonoid must be properly absorbed and metabolized from the moth's diet for successful green pigmentation of the cocoon. However, the major food source of *A. yamamai* is oak leaf, unlike *B.mori* who feed primarily on mulberry leaf. Gene ontology pathway analysis showed that major functions of specific gene family clusters expanded in *A. yamamai* genome are related to the flavonoid metabolism and detoxification of diet, which suggests that specific gene family expansion

related to food intake may reflect the impact of the environmental adaptation process on the *A. yamamai* genome.

The constructed genome of *A.yamamai* presented here is the first announced genome in family Saturniidae and we expect that this genome will provide valuable information for future research. Although *A. yamamai* and *B. mori* appear similar, comparative genome analysis of the two species uncovered significant differences in the genome evolution processes between families Saturniidae and Bombycidae. Therefore, this constructed genome provides more insight into the genome evolution and phylogeny of family Saturniidae, which contains the largest number of species in Lepitoptera. Most previous phylogenetic studies were limited to few genes due to the lack of genomic information on family Saturniidae. We expect our study and resulting constructed genome will resolve some limitations of molecular phylogenetic and ecological research on Saturniidae species. And constructed genome information will help researchers better understand the molecular background of wild silk and its production. Silk produced by *A. yamamai*, referred to as *tensan* silk, shows unique characteristics which has made it valuable in various fields. However, *A. yamam*ai has not been completely domesticated compared to *B. mori*, making mass production of tensan silk infeasible. Understanding of the molecular mechanisms behind the tensan silk production process is essential for mass production using biotechnology, and we expect that our result will be a fundamental resource for related research and industrial improvement. Additionally, the transcriptome data of 10 different organ tissues with 3 biological replications presented here may be also useful resources for uncovering the molecular mechanisms related to specific phenotypes of *A.yamamai* and family Saturniidae.

Availability of supporting data

The generated genome sequence and gene information of *A. yamamai* are available in GigaDB[66] and generated raw data is available under project accession PRJNA383008 and PRJNA383025 of the NCBI database.

Competing interests

All authors report no competing interests.

Abbreviation

RBH – Reciprocal Best Hit

PSMC - Pairwise Sequentially Markovian Coalescent

Authors contributions

Sampling - Kee-Young Kim, Su-Bae Kim

Sequencing - Kwang-Ho Choi, Seong-Wan Kim

Genome assembly - Seong-Ryul Kim, Woori Kwak, Jae-Sam Hwang, Seung-Won Park

Repeat element analysis - Seong-Ryul Kim, Woori Kwak, Seung-Won Park

Gene prediction - Seong-Ryul Kim, Woori Kwak, Jae-Sam Hwang

Comparative genome analysis - Seong-Ryul Kim, Woori Kwak, Min-Jae Kim, Kelsey Caetano-Anolles

Funding and experimental design - Seong-Ryul Kim, Seung-Won Park

Acknowledgements

This work was supported by a grant from the Rural Development Administration, Republic of Korea (grant no. PJ010442).

References

1. Regier, J.C., et al., *Phylogenetic relationships of wild silkmoths (Lepidoptera: Saturniidae) inferred from four protein-coding nuclear genes.* Systematic Entomology, 2008. **33**(2): p. 219-228.
2. Regier, J.C., et al., *A large-scale, higher-level, molecular phylogenetic study of the insect order Lepidoptera (moths and butterflies).* PLoS One, 2013. **8**(3): p. e58568.
3. Hedges, S.B., J. Dudley, and S. Kumar, *TimeTree: a public knowledge-base of divergence times among organisms.* Bioinformatics, 2006. **22**(23): p. 2971-2972.
4. Kawahara, A.Y. and J.R. Barber, *Tempo and mode of antibat ultrasound production and sonar jamming in the diverse hawkmoth radiation.* Proceedings of the National Academy of Sciences, 2015. **112**(20): p. 6407-6412.
5. Peigler, R.S., *Wild silks of the world.* American Entomologist, 1993. **39**(3): p. 151-162.
6. Nakamura, S., et al., *Physical properties and structure of silk. XI. Glass transition temperature of wild silk fibroins.* Journal of applied polymer science, 1986. **31**(3): p. 955-956.
7. 松本陽一 and 斎藤英毅, *Load-extension characteristics of composite raw silk of Antheraea yamamai and Bombyx mori.* 日本蚕糸学雑誌, 1997. **66**(6): p. 497-501.
8. Kweon, H. and Y. Park, *Structural characteristics and physical properties of wild silk fibres; Antheraea pernyi and Antheraea yamamai.* Korean Journal of Sericultural Science (Korea Republic), 1994.
9. Zheng, Z., et al., *Preparation of regenerated Antheraea yamamai silk fibroin film and controlled-molecular conformation changes by aqueous ethanol treatment.* Journal of applied polymer science, 2010. **116**(1): p. 461-467.
10. Omenetto, F., et al., *Silk based biophotonic sensors*. 2011, Google Patents.
11. Takeda, S., *New field of insect science: Research on the use of insect properties.* Entomological Science, 2013. **16**(2): p. 125-135.
12. Omenetto, F. and D.L. Kaplan, *Silk-based multifunctional biomedical platform*. 2012, Google Patents.
13. Serban, M.A., *Silk medical devices*. 2016, Google Patents.
14. Jiang, G.-L., et al., *Drug delivery platforms comprising silk fibroin hydrogels and uses thereof*. 2010, Google Patents.
15. Kamiya, M., et al., *Structure–activity relationship of a novel pentapeptide with cancer cell growth-inhibitory activity.* Journal of Peptide Science, 2010. **16**(5): p. 242-248.
16. Bioinformatics, B., *FastQC A quality control tool for high throughput sequence data.* Cambridge, UK: Babraham Institute, 2011.
17. Bolger, A.M., M. Lohse, and B. Usadel, *Trimmomatic: a flexible trimmer for Illumina sequence data.* Bioinformatics, 2014: p. btu170.

18. Marçais, G. and C. Kingsford, *A fast, lock-free approach for efficient parallel counting of occurrences of k-mers.* Bioinformatics, 2011. **27**(6): p. 764-770.
19. You, M., et al., *A heterozygous moth genome provides insights into herbivory and detoxification.* Nature genetics, 2013. **45**(2): p. 220-225.
20. Gnerre, S., et al., *High-quality draft assemblies of mammalian genomes from massively parallel sequence data.* Proceedings of the National Academy of Sciences, 2011. **108**(4): p. 1513-1518.
21. Luo, R., et al., *SOAPdenovo2: an empirically improved memory-efficient short-read de novo assembler.* Gigascience, 2012. **1**(1): p. 18.
22. O'Connell, J., et al., *NxTrim: optimized trimming of Illumina mate pair reads.* Bioinformatics, 2015. **31**(12): p. 2035-2037.
23. Boetzer, M., et al., *Scaffolding pre-assembled contigs using SSPACE.* Bioinformatics, 2011. **27**(4): p. 578-579.
24. Boetzer, M. and W. Pirovano, *SSPACE-LongRead: scaffolding bacterial draft genomes using long read sequence information.* BMC bioinformatics, 2014. **15**(1): p. 211.
25. Voskoboynik, A., et al., *The genome sequence of the colonial chordate, Botryllus schlosseri.* Elife, 2013. **2**: p. e00569.
26. McCoy, R.C., et al., *Illumina TruSeq synthetic long-reads empower de novo assembly and resolve complex, highly-repetitive transposable elements.* PloS one, 2014. **9**(9): p. e106689.
27. Simão, F.A., et al., *BUSCO: assessing genome assembly and annotation completeness with single-copy orthologs.* Bioinformatics, 2015: p. btv351.
28. Bao, Z. and S.R. Eddy, *Automated de novo identification of repeat sequence families in sequenced genomes.* Genome Research, 2002. **12**(8): p. 1269-1276.
29. Price, A.L., N.C. Jones, and P.A. Pevzner, *De novo identification of repeat families in large genomes.* Bioinformatics, 2005. **21**(suppl 1): p. i351-i358.
30. Benson, G., *Tandem repeats finder: a program to analyze DNA sequences.* Nucleic acids research, 1999. **27**(2): p. 573.
31. Kohany, O., et al., *Annotation, submission and screening of repetitive elements in Repbase: RepbaseSubmitter and Censor.* BMC bioinformatics, 2006. **7**(1): p. 474.
32. Tarailo-Graovac, M. and N. Chen, *Using RepeatMasker to identify repetitive elements in genomic sequences.* Current Protocols in Bioinformatics, 2009: p. 4.10. 1-4.10. 14.
33. Bao, W., K.K. Kojima, and O. Kohany, *Repbase Update, a database of repetitive elements in eukaryotic genomes.* Mobile DNA, 2015. **6**(1): p. 11.
34. Nene, V., et al., *Genome sequence of Aedes aegypti, a major arbovirus vector.* Science, 2007. **316**(5832): p. 1718-1723.
35. Xia, Q., et al., *A draft sequence for the genome of the domesticated silkworm (Bombyx mori).* Science, 2004. **306**(5703): p. 1937-1940.
36. Zhan, S., et al., *The monarch butterfly genome yields insights into long-distance migration.* Cell, 2011. **147**(5): p. 1171-1185.

37. Adams, M.D., et al., *The genome sequence of Drosophila melanogaster.* Science, 2000. **287**(5461): p. 2185-2195.
38. Consortium, H.G., *Butterfly genome reveals promiscuous exchange of mimicry adaptations among species.* Nature, 2012. **487**(7405): p. 94-98.
39. Ahola, V., et al., *The Glanville fritillary genome retains an ancient karyotype and reveals selective chromosomal fusions in Lepidoptera.* Nature communications, 2014. **5**.
40. Stanke, M., et al., *Using native and syntenically mapped cDNA alignments to improve de novo gene finding.* Bioinformatics, 2008. **24**(5): p. 637-644.
41. Blanco, E., G. Parra, and R. Guigó, *Using geneid to identify genes.* Current protocols in bioinformatics, 2007: p. 4.3. 1-4.3. 28.
42. Lomsadze, A., P.D. Burns, and M. Borodovsky, *Integration of mapped RNA-Seq reads into automatic training of eukaryotic gene finding algorithm.* Nucleic acids research, 2014: p. gku557.
43. Trapnell, C., L. Pachter, and S.L. Salzberg, *TopHat: discovering splice junctions with RNA-Seq.* Bioinformatics, 2009. **25**(9): p. 1105-1111.
44. Trapnell, C., et al., *Differential gene and transcript expression analysis of RNA-seq experiments with TopHat and Cufflinks.* Nature protocols, 2012. **7**(3): p. 562-578.
45. Campbell, M.A., et al., *Comprehensive analysis of alternative splicing in rice and comparative analyses with Arabidopsis.* BMC genomics, 2006. **7**(1): p. 327.
46. Haas, B.J., et al., *Automated eukaryotic gene structure annotation using EVidenceModeler and the Program to Assemble Spliced Alignments.* Genome biology, 2008. **9**(1): p. R7.
47. Consortium, U., *Reorganizing the protein space at the Universal Protein Resource (UniProt).* Nucleic acids research, 2011: p. gkr981.
48. Pruitt, K.D., T. Tatusova, and D.R. Maglott, *NCBI reference sequences (RefSeq): a curated non-redundant sequence database of genomes, transcripts and proteins.* Nucleic acids research, 2007. **35**(suppl 1): p. D61-D65.
49. Jones, P., et al., *InterProScan 5: genome-scale protein function classification.* Bioinformatics, 2014. **30**(9): p. 1236-1240.
50. Li, H. and R. Durbin, *Inference of human population history from individual whole-genome sequences.* Nature, 2011. **475**(7357): p. 493-496.
51. Li, H., et al., *The sequence alignment/map format and SAMtools.* Bioinformatics, 2009. **25**(16): p. 2078-2079.
52. Li, L., C.J. Stoeckert, and D.S. Roos, *OrthoMCL: identification of ortholog groups for eukaryotic genomes.* Genome research, 2003. **13**(9): p. 2178-2189.
53. Löytynoja, A. and N. Goldman, *An algorithm for progressive multiple alignment of sequences with insertions.* Proceedings of the National Academy of Sciences of the United States of America, 2005. **102**(30): p. 10557.
54. Castresana, J., *Selection of conserved blocks from multiple alignments for their use in phylogenetic analysis.* Molecular biology and evolution, 2000. **17**(4): p. 540-552.

55. Tamura, K., et al., *MEGA6: molecular evolutionary genetics analysis version 6.0.* Molecular biology and evolution, 2013: p. mst197.
56. Ronquist, F. and J.P. Huelsenbeck, *MrBayes 3: Bayesian phylogenetic inference under mixed models.* Bioinformatics, 2003. **19**(12): p. 1572-1574.
57. Posada, D., *Using MODELTEST and PAUP* to select a model of nucleotide substitution.* Current protocols in bioinformatics, 2003: p. 6.5. 1-6.5. 14.
58. De Bie, T., et al., *CAFE: a computational tool for the study of gene family evolution.* Bioinformatics, 2006. **22**(10): p. 1269-1271.
59. Huang, D.W., B.T. Sherman, and R.A. Lempicki, *Systematic and integrative analysis of large gene lists using DAVID bioinformatics resources.* Nature protocols, 2009. **4**(1): p. 44-57.
60. Daimon, T., et al., *The silkworm Green b locus encodes a quercetin 5-O-glucosyltransferase that produces green cocoons with UV-shielding properties.* Proceedings of the National Academy of Sciences, 2010. **107**(25): p. 11471-11476.
61. Yoshiomi, K., et al., *Role of light in the green pigmentation of cocoons of Antheraea yamamai (Lepidoptera: Saturniidae).* Applied Entomology and Zoology, 1989. **24**(4): p. 398-406.
62. Harizuka, M., *Physiological genetics of the carotenoids in Bombyx mori, with special reference to the pink cocoon.* Bull Seric Exp Stn Japan, 1953. **14**: p. 141-156.
63. Tazima, Y., *The silkworm: an important laboratory tool.* 1978.
64. Hirayama, C., et al., *Regioselective formation of quercetin 5-O-glucoside from orally administered quercetin in the silkworm, Bombyx mori.* Phytochemistry, 2008. **69**(5): p. 1141-1149.
65. Tamura, Y., et al., *Flavonoid 5-glucosides from the cocoon shell of the silkworm, Bombyx mori.* Phytochemistry, 2002. **59**(3): p. 275-278.
66. Sneddon, T.P., P. Li, and S.C. Edmunds, *GigaDB: announcing the GigaScience database.* GigaScience, 2012. **1**(1): p. 11.

Tables

Table 1. Summary statistics of generated whole genome shotgun sequencing data using Illumina Nextseq 500.

Library Name	Library Type	Insert Size	Platform	Read Length	No. Read	Total bp
350bp	Paired-end	350bp	Nextseq500	151	293,176,268	44,269,616,468
700bp	Paired-end	700bp	Nextseq500	151	246,945,900	37,288,830,900
3Kbp	Mate-pair	3Kbp	Nextseq500	76	284,204,762	21,599,561,912
6Kbp	Mate-pair	6Kbp	Nextseq500	76	246,238,370	18,714,116,120
9Kbp	Mate-pair	9Kbp	Nextseq500	76	239,919,538	18,233,884,888
**Total**						140,106,010,288

Table 2. Summary statistics of generated Illumina synthetic long read (Moleculo) library.

	500-1499bp	>= 1500bp
Number of assembled read	302,132	342,738
Number of bases in assembled read	268,853,717	1,205,349,082
N50 length of assembled read	960	4,031

Table 3. Summary statistics of generated long reads data using Pacbio RS II system.

Number of Reads	1,005,571
Total Bases	5,836,969,225
Length of longest (shortest) read	50,132(50)
Average read length	5,804.63

Table 4. Summary statistics of generated transcriptome data obtained from six organ tissues using Illumina platform.

Tissue	Sample Name	Read Length	Read Count	Total Base (bp)
Hemocyte	Hemocyte_1	76	20,815,674	1,581,991,224
	Hemocyte_2	76	26,704,666	2,029,554,616
	Hemocyte_2	76	53,068,562	4,033,210,712
Malpighi	Malpighi_1	76	22,635,428	1,720,292,528
	Malpighi_2	76	24,893,788	1,891,927,888
	Malpighi_3	76	45,213,164	3,436,200,464
Midgut	Midgut_1	76	23,350,138	1,774,610,488
	Midgut_2	76	24,597,972	1,869,445,872
	Midgut_3	76	50,949,986	3,872,198,936
Head	Head_1	76	26,526,276	2,015,996,976
	Head_2	76	26,581,124	2,020,165,424
	Head_3	76	40,900,456	3,108,434,656
Skin	Skin_1	76	24,592,846	1,869,056,296
	Skin_2	76	42,775,430	3,250,932,680
	Skin_3	76	35,043,570	2,663,311,320
Fat Body	Fat Body_1	76	24,637,810	1,872,473,560
	Fat Body_2	76	24,037,494	1,826,849,544
	Fat Body_3	76	40,817,582	3,102,136,232
AM/Silk Gland	AM/Silk Gland_1	76	21,399,638	1,626,372,488
	AM/Silk Gland_2	76	24,292,386	1,846,221,336
	AM/Silk Gland_3	76	37,331,530	2,837,196,280
P/Silk Gland	P/Silk Gland_1	76	27,359,580	2,079,328,080
	P/Silk Gland_2	76	23,300,962	1,770,873,112
	P/Silk Gland_3	76	39,421,430	2,996,028,680
Testis	Testis_1	76	40,890,404	3,107,670,704
	Testis_2	76	45,733,846	3,475,772,296
	Testis_3	76	44,985,224	3,418,877,024
Ovary	Ovary_1	76	40,797,628	3,100,619,728
	Ovary_2	76	40,409,752	3,071,141,152
	Ovary_3	76	42,417,892	3,223,759,792

Table 5. Summary statistics of the *A. yamamai* genome (>2kb).

**Assembled Genome**	
Size(1n)	656 Mb
GC level	34.07
No. scaffolds	3,675
N50 of scaffolds (bp)	739,388
N bases in scaffolds (%)	19,257,439 (2.93)
Longest(shortest) scaffolds (bp)	3,156,949 (2,003)
Average scaffold Length (bp)	178,657.53

**Repeat Element**	No. Element	Length (%)
SINE	59,968	8,615,338(1.30)
LINE	426,522	101,251,176(15.31)
LTR element	53,977	4,552,386(0.69)
DNA element	512,760	69,071,227(10.44)
Small RNA	43,645	6,691,619(1.01)
Simple repeat	135,989	6,256,839(0.95)
Low complexity	19,937	932,829(0.14)
Unclassified	294,190	54,552,009(8.25)

Table 6. Summary of identified repeat elements in the *A. yamamai* genome.

Table 7. Summary statistics of ab initio, RNA-seq based and homology-based gene prediction results.

Evidence Type	Programs	Element	Total count	Exon/Gene	Total length(bp)	Mean length(bp)
ab_initio	Augustus	Gene	14,576	4.85	142,415,318	9,770.53
		Exon	70,733		14,736,668	208.34
	Geneid	Gene	10,946	2.25	46,119,402	4,213.35
		Exon	24,686		3,925,563	159.01
	GeneMarks-ET	Gene	27,754	5.50	273,745,951	9,863.29
		Exon	152,660		30,847,503	202.06
RNA-seq	Cufflinks Transdecoder	Gene	36,213	7.03	840,429,061	23,207.94
		Exon	254,770		201,721,675	791.77
Known Gene (NCBI lepidoptera)	PASA (gmap)		44,561		22,484,151	504.57

Table 8. Summary statistics for the consensus gene set of the *A. yamamai* genome.

Element	No. elements	Exon/Gene	Avg. length	Total length	Genome coverage
Gene	21,124	4.44	8,331.63	175,997,473	26.61
Exon	93,950		236.53	22,222,354	3.35

Figures

Figure 1. Photograph of *Antheraea Yamamai*. From left- larva, cocoon and adult *A. yamamai*, respectively. Green color is one of the representative characteristics of tensan silk.

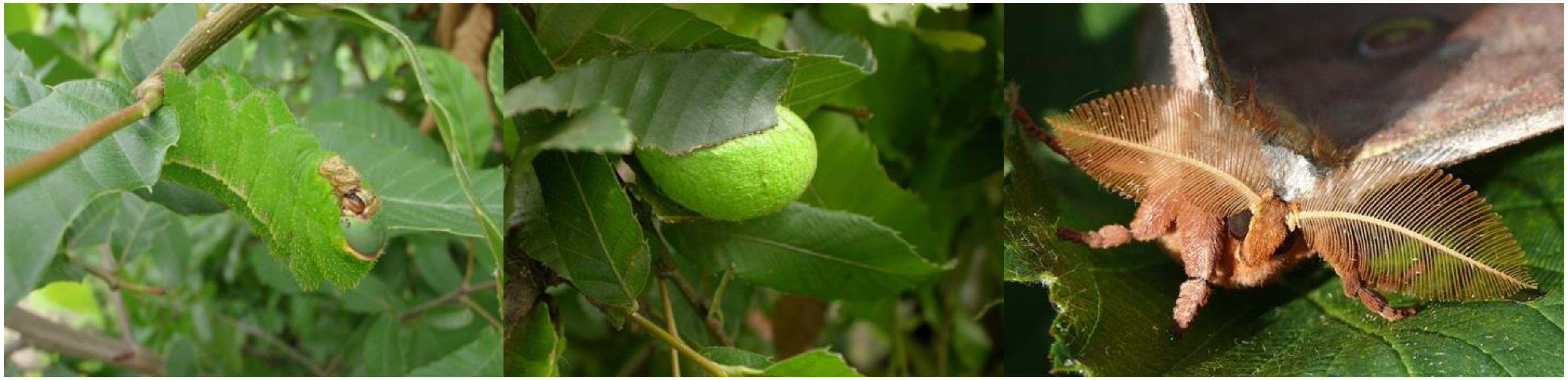

Figure 2. Karyotype of *A.yamamai* using a gamete of testis in metaphase.

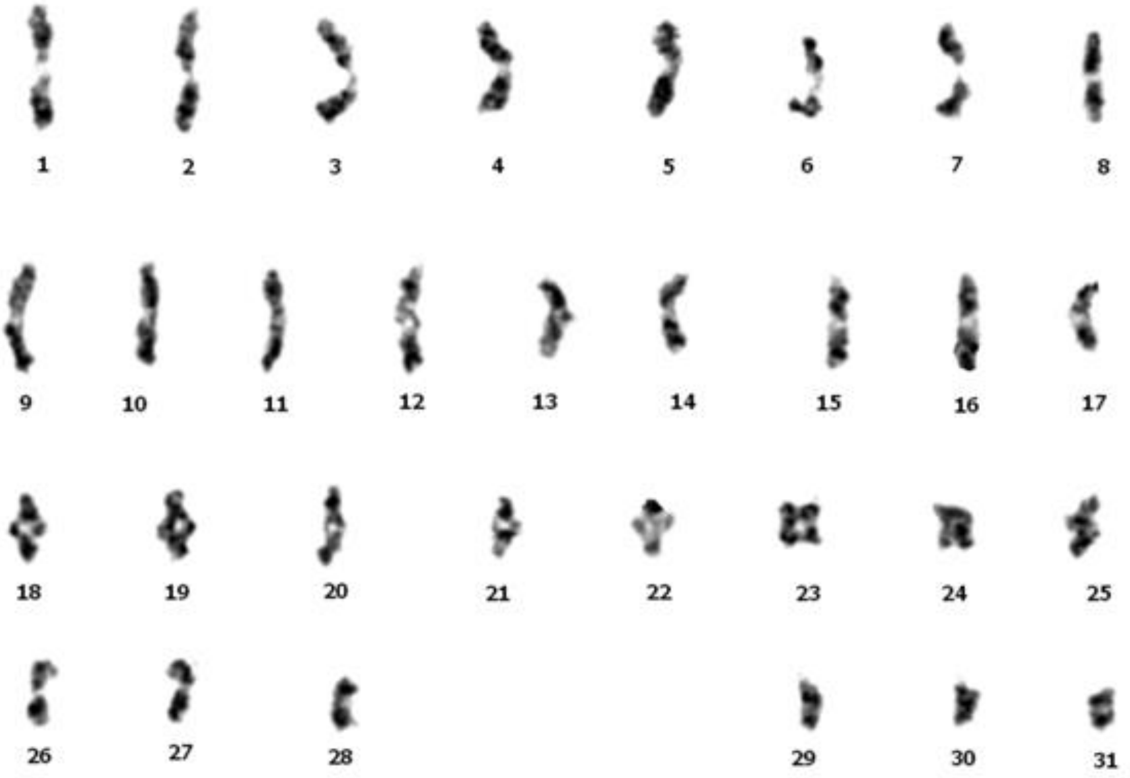

Figure 3. 19-mer distribution of *A. yamamai* genome using jellyfish with 350bp paired-end whole genome sequencing data.

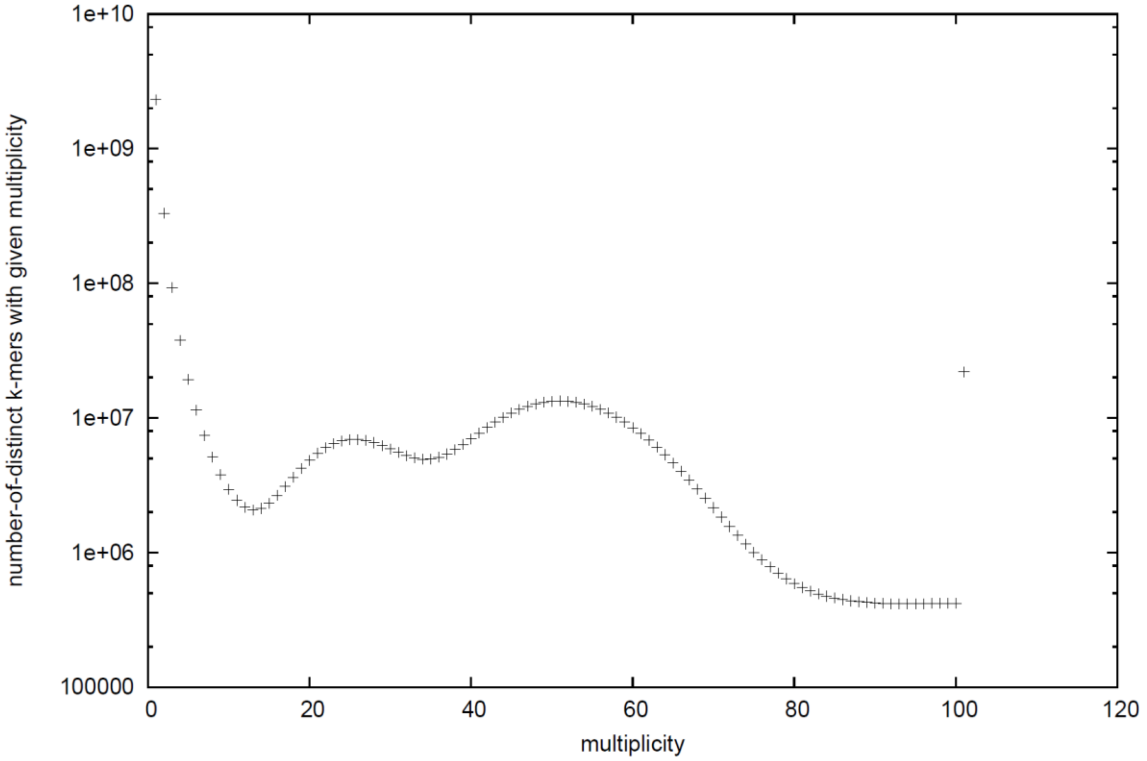

Figure 4. Amount and proportion of identified repeat element from 8 species including *A. yamamai*. a. Absolute amount of repeat element classified into 8 different categories. b. Proportion of each repeat element in identified total repeat element.

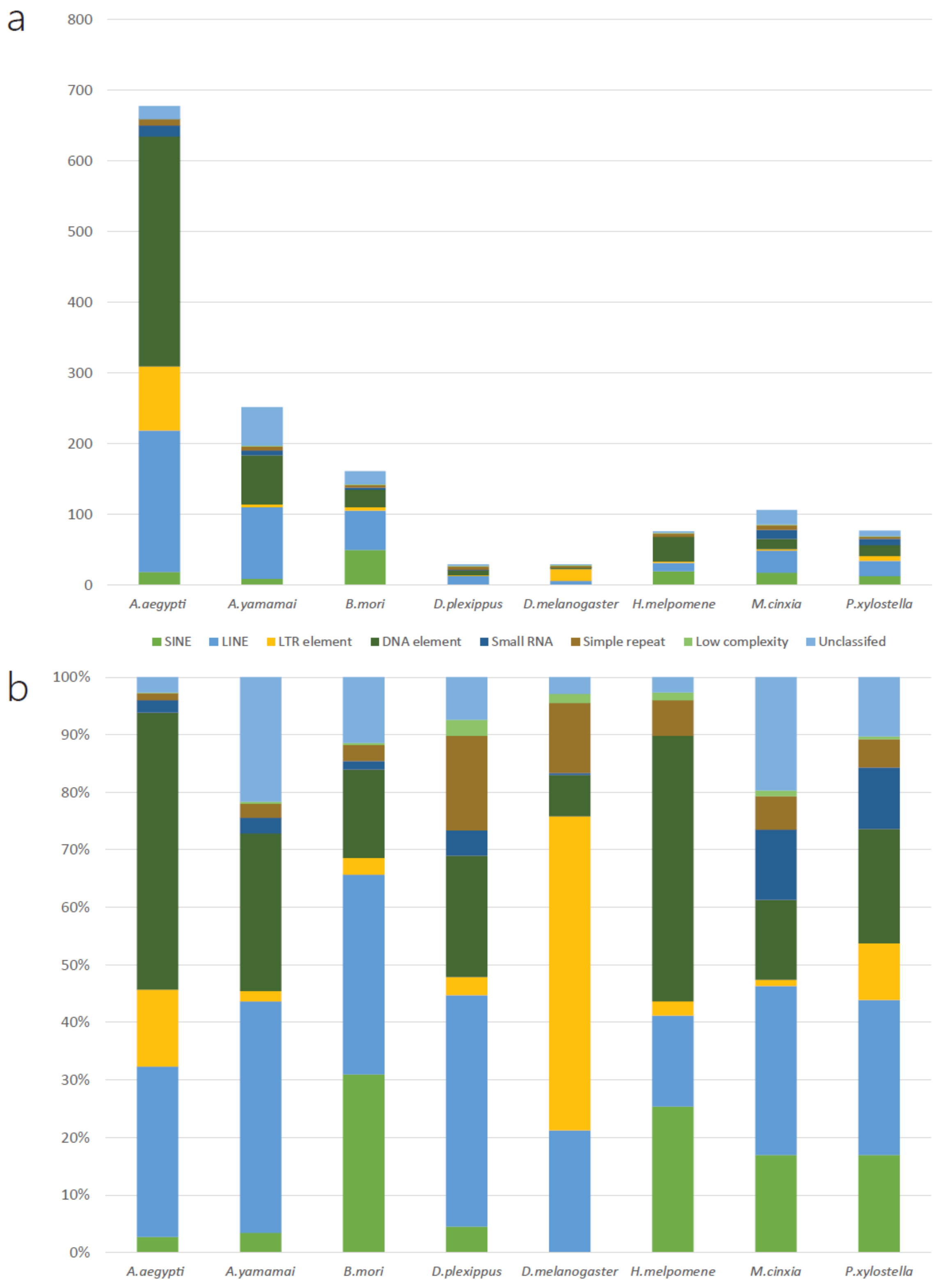

Figure 5. Demographic history of *A. yamamai* using PSMC and comparative gene family analysis. Node value indicate Bayesian posterior probability, bootstrap and gene expansion, contraction value. Orange and blue color indicate expansion and contraction, respectively.

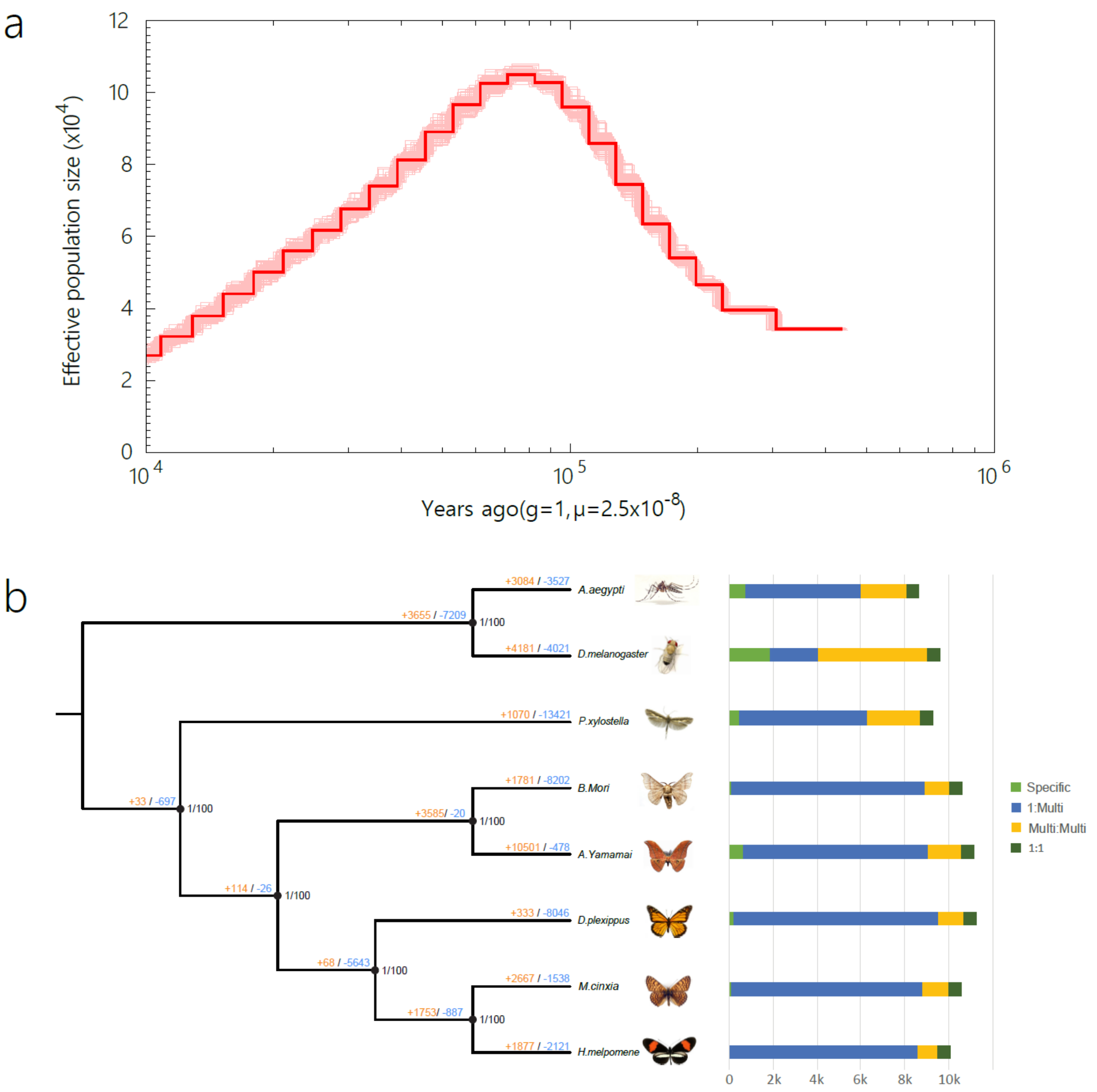

Figure

Click here to download Figure Figure1.png

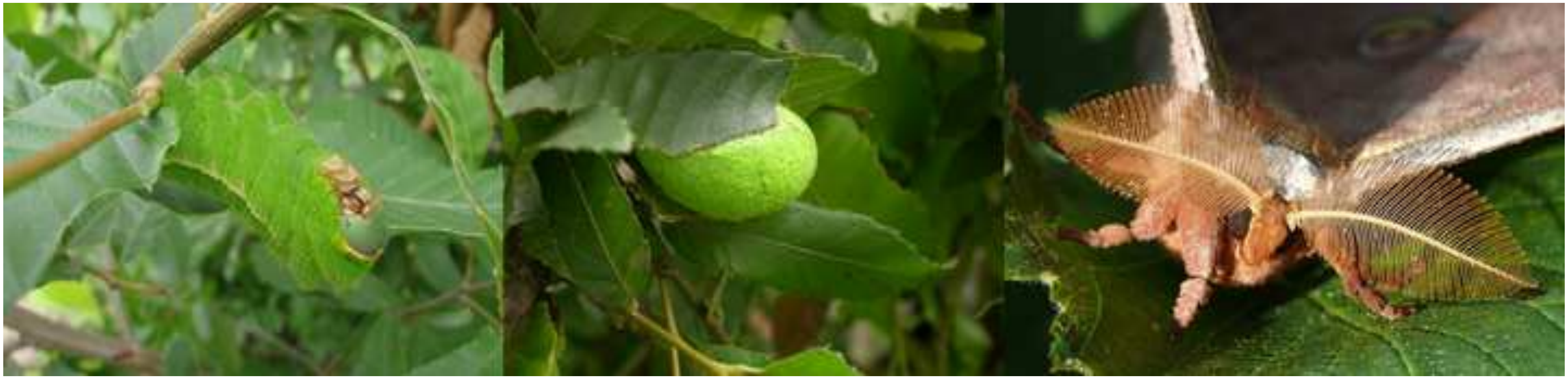

 Click here to download Figure Figure2.jpg

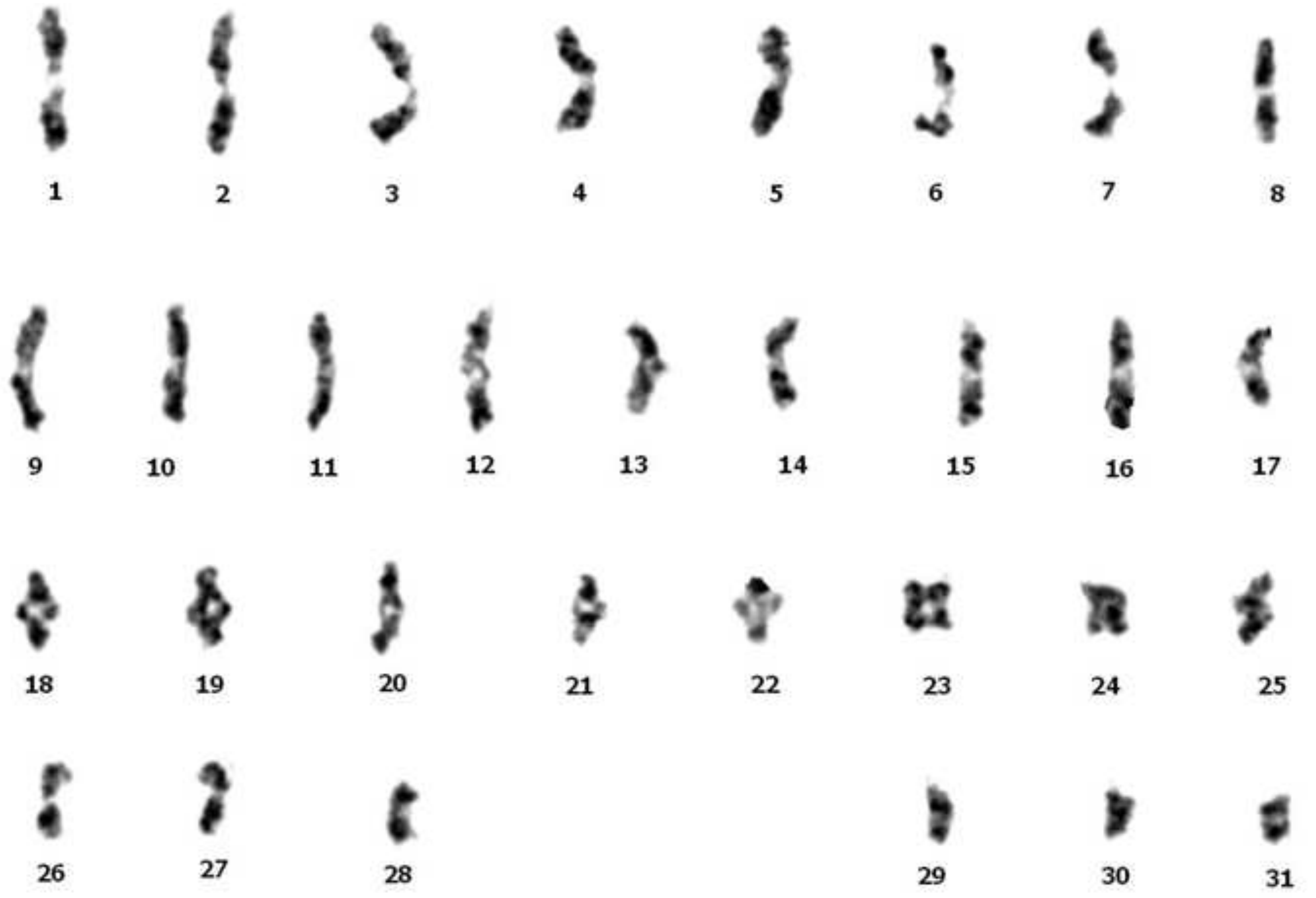

 Click here to download Figure Figure3.pdf

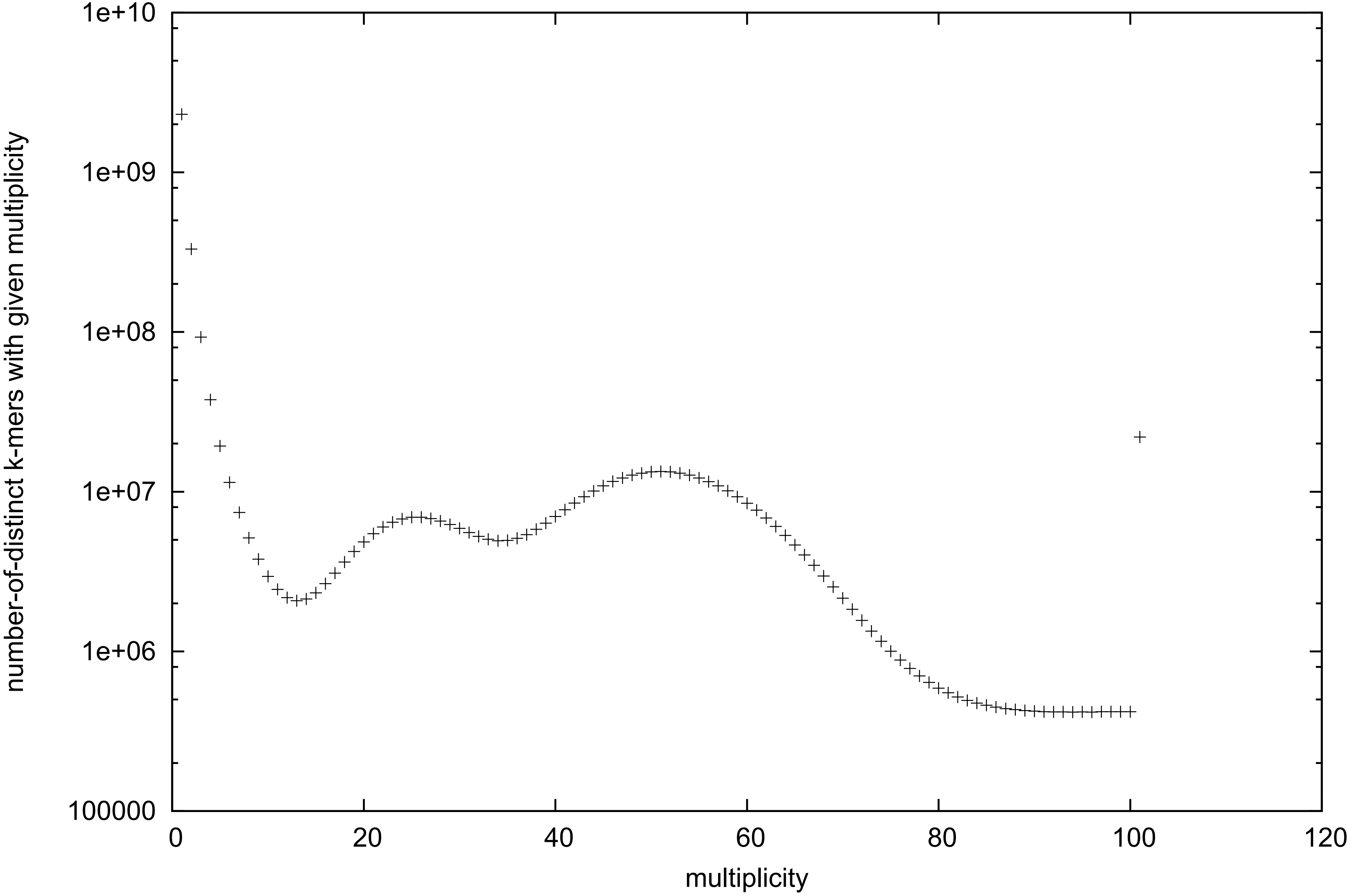

Figure

Click here to download Figure Figure4.pdf

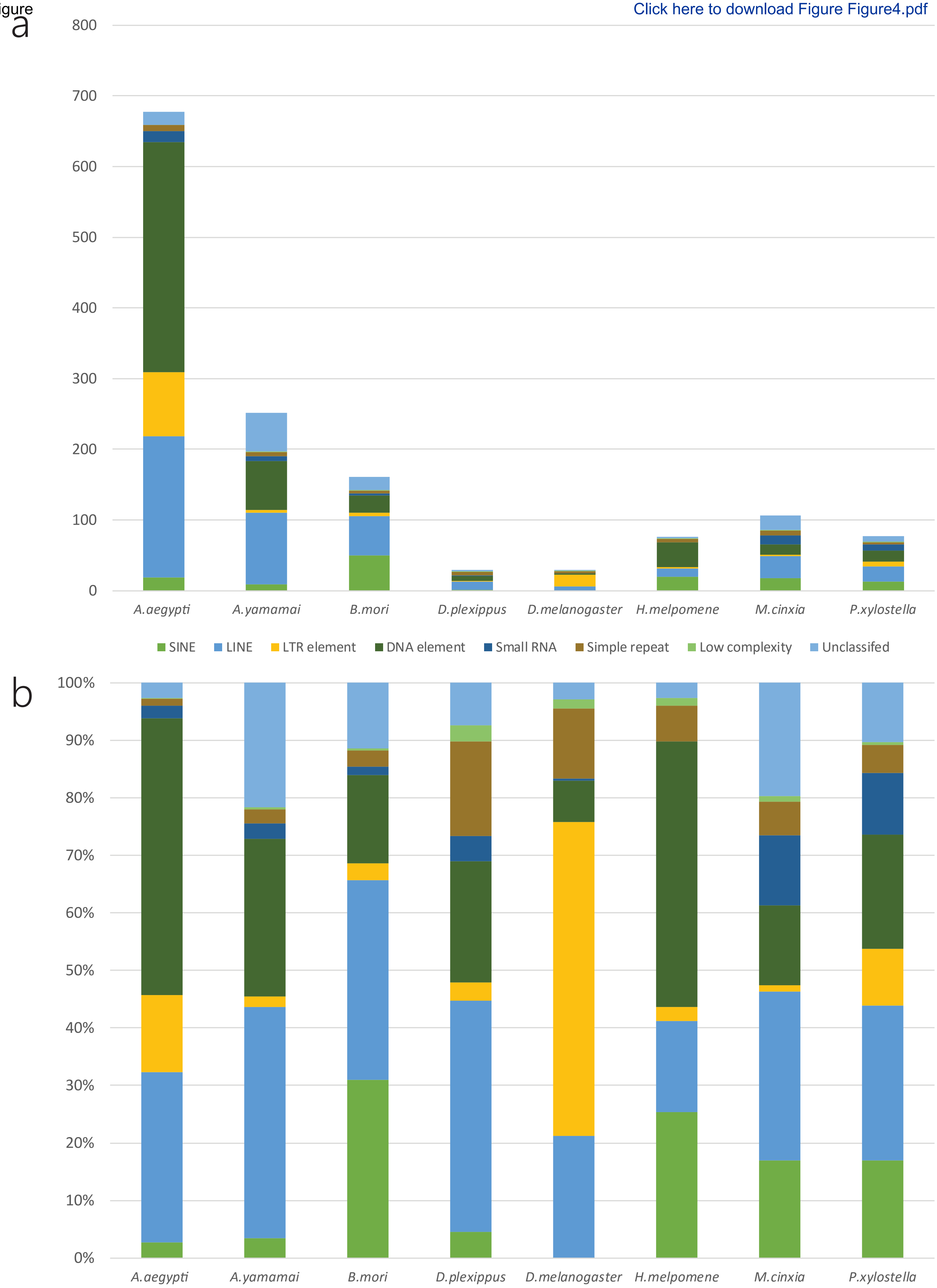

Figure
Click here to download Figure Figure5.pdf

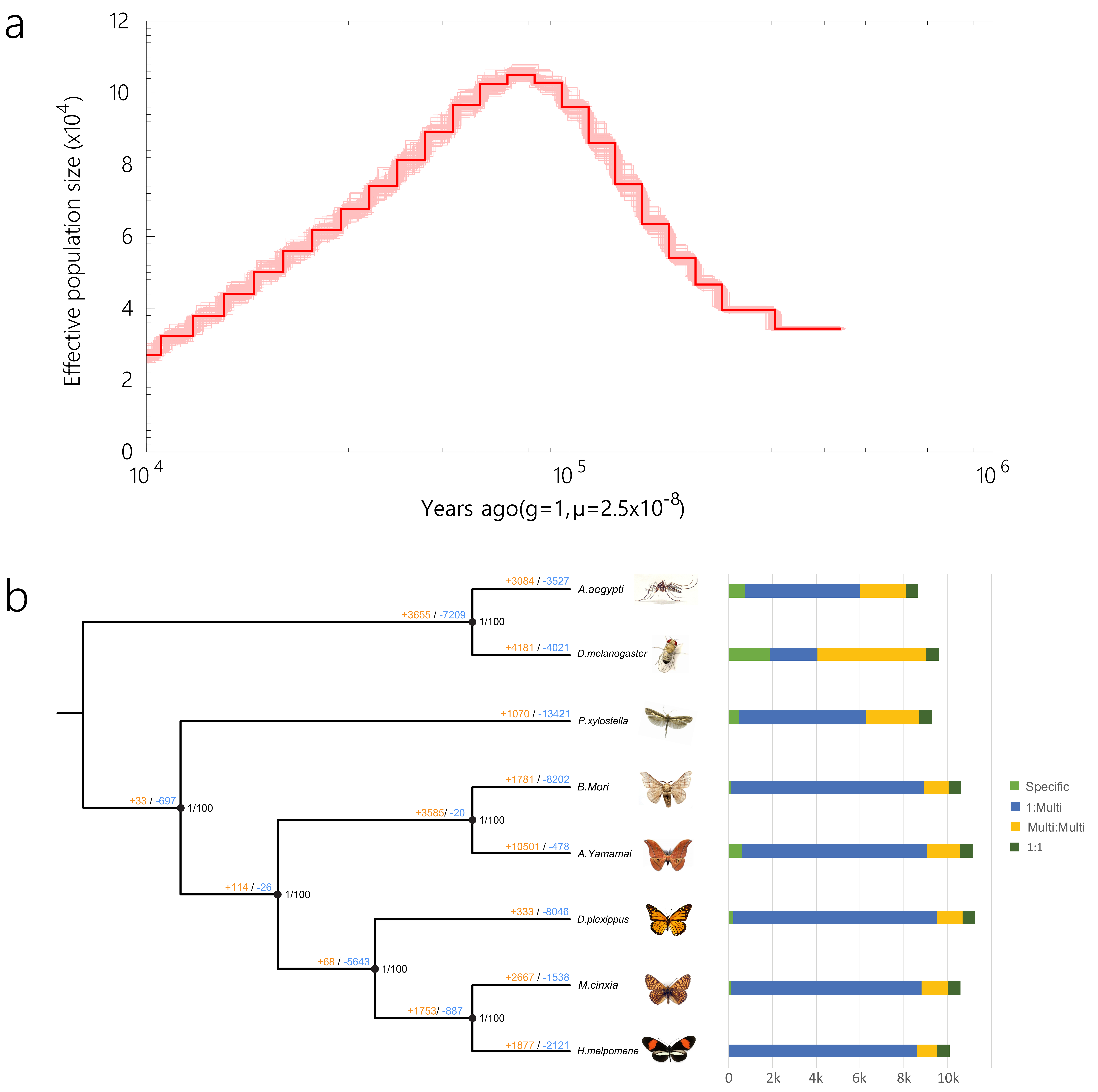

Click here to access/download
Supplementary Material
Supplementary_Information.docx

Click here to access/download
Supplementary Material
Supplementrary_information2.xlsx

Personal Cover Click here to download Personal Cover Cover_letter2.docx

, May 22, 2017

Dear Editor of *Gigascience*,

I am pleased to submit our research article entitled "Genome sequence of Japanese oak silk moth, *Antheraea yamamai*: the first draft genome in family Saturniidae", to your reputed journal, ***Gigascience***.

Unlike *Bombyx mori*, few studies have investigated the genomic information for the wild-type silk moth. Wild silk moth, *A. yamamai and A. perny*, are moth genus belonging to the family Saturniidae and which produce wild silk of commercial importance. In this article, we attempted to the whole-genome sequencing for the *A. yamamai*, thereby we constructed genome of *A. yamamai* were 656 Mb(>2kb) with 3,675 scaffolds and N50 length of assembly was 739 Kb with 34.07% GC ratio. To the best of our knowledge, these results will provide valuable genomic information for understanding the molecular mechanisms related to the specific phenotypes such as wild silk itself, and more insight into Saturniidae phylogeny and genome evolution process.

The material is original research, has not been previously published and has not been submitted for publication elsewhere while under consideration. The authors have declared that they have no conflict of interest.

I hope this paper can meet your approval and can be published at the earliest possible date.

Looking forward to hearing from you again.
Thank you.

With best regards,

Prof. Seung-Won Park
Department of Biotechnology,
Catholic University of Daegu, Gyeongsan-si, Gyeongsangbuk-do 38430, Republic of Korea,
Tel: +82-53-850-3176, E-mail: microsw@cu.ac.kr
