# Supplementary material for: Genome sequence of the Japanese oak silk moth, Antheraea yamamai: the first draft genome in the family Saturniidae
Source: Gigascience. 2017 Nov 27;7(1):gix113. doi: 10.1093/gigascience/gix113 (PMC5774507; doi:10.1093/gigascience/gix113)
Supplement: GIGA-D-17-00085_Revision-2.pdf [file gix113_giga-d-17-00085_revision-2.pdf]

GigaScience

Genome sequence of Japanese oak silk moth, Antheraea yamamai: the first draft genome in family Saturniidae

--Manuscript Draft--

Manuscript Number:	GIGA-D-17-00085R2
Full Title:	Genome sequence of Japanese oak silk moth, Antheraea yamamai: the first draft genome in family Saturniidae
Article Type:	Data Note
Funding Information:	Rural Development Administration (PJ010442) / Dr Seong-Ryul Kim
Abstract:	Background<br>Antheraea yamamai, also known as the Japanese oak silk moth, is a wild species of the silk moth. Silk produced by A. yamamai, referred to as tensan silk, shows different characteristics such as thickness, compressive elasticity and chemical resistance compared to the common silk produced from the domesticated silkworm, Bombyx mori. Its unique characteristics have led to its use in many research fields including biotechnology and medical science, and the scientific as well as economic importance of wild silk moth continues to gradually increase. However, no genomic information for wild silk moth, including A. yamamai, is currently available.<br><br>Findings<br>In order to construct the A. yamamai genome, a total of 147G base pairs using Illumina and Pacbio sequencing platforms were generated, providing 210-fold coverage based on the 700 Mb estimated genome size of A. yamamai. The assembled genome of A. yamamai was 656 Mb(>2kb) with 3,675 scaffolds and the N50 length of assembly was 739 Kb with 34.07% GC ratio. Identified repeat elements covered 37.33% of the total genome and the completeness of the constructed genome assembly was estimated to be 96.7% by BUSCO v2 analysis. A total of 21,124 genes were identified using Evidence Modeler based on the gene prediction results obtained from 3 different methods (ab initio, RNA-seq based, known-gene based).<br><br>Conclusions<br>Here we present the genome sequence of A. yamamai, the first genome sequence of wild silk moth. These results provide valuable genomic information which will help enrich our understanding of the molecular mechanisms related to not only specific phenotypes such as wild silk itself but also the genomic evolution of Saturniidae.
Corresponding Author:	Seung-Won Park<br><br>KOREA, REPUBLIC OF
Corresponding Author Secondary Information:	
Corresponding Author's Institution:	
Corresponding Author's Secondary Institution:	
First Author:	Seong-Ryul Kim
First Author Secondary Information:	
Order of Authors:	Seong-Ryul Kim
	Woori Kwak
	Hyaekang Kim
	Kelsey Caetano-Anolles
	Kee-Young Kim
	Su-Bae Kim
	Kwang-Ho Choi

Powered by Editorial Manager® and ProduXion Manager® from Aries Systems Corporation

	Seong-Wan Kim
	Jae-Sam Hwang
	Min-Jee Kim
	Iksoo Kim
	Tae-Won Goo
	Seung-Won Park
**Order of Authors Secondary Information:**	
**Response to Reviewers:**	We appreciate suggestions of all the reviewers on our manuscript; all the comments made by the reviewers were quite valid. We have responded to all comments point-by-point in the seperate rebuttal. We hope our revised manuscript can meet the quality standard of reviewers.
**Additional Information:**	
**Question**	**Response**
Are you submitting this manuscript to a special series or article collection?	No
**Experimental design and statistics**<br><br>Full details of the experimental design and statistical methods used should be given in the Methods section, as detailed in our Minimum Standards Reporting Checklist. Information essential to interpreting the data presented should be made available in the figure legends.<br><br>Have you included all the information requested in your manuscript?	Yes
**Resources**<br><br>A description of all resources used, including antibodies, cell lines, animals and software tools, with enough information to allow them to be uniquely identified, should be included in the Methods section. Authors are strongly encouraged to cite Research Resource Identifiers (RRIDs) for antibodies, model organisms and tools, where possible.<br><br>Have you included the information requested as detailed in our Minimum Standards Reporting Checklist?	Yes
**Availability of data and materials**<br><br>All datasets and code on which the conclusions of the paper rely must be	Yes

either included in your submission or deposited in publicly available repositories (where available and ethically appropriate), referencing such data using a unique identifier in the references and in the "Availability of Data and Materials" section of your manuscript.<br><br>Have you have met the above requirement as detailed in our Minimum Standards Reporting Checklist?	

Manuscript Click here to download Manuscript Manuscript_Rev_ENG.docx

Genome sequence of Japanese oak silk moth, *Antheraea yamamai*: the first draft genome in family Saturniidae

Seong-Ryul Kim[1†], Woori Kwak[2†], Hyaekang Kim[3], Kelsey Caetano-Anolles[3], Kee-Young Kim[1], Su-Bae Kim[1], Kwang-Ho Choi[1], Seong-Wan Kim[1], Jae-Sam Hwang[1], Min-Jee Kim[4], Iksoo Kim[4], Tae-Won Goo[5] and Seung-Won Park[6]*

[1]Department of Agricultural Biology, National Academy of Agricultural Science, Rural Development Administration, Wanju-gun 55365, Republic of Korea; [2]C&K Genomics, Main Bldg. #420, SNU Research Park, Seoul 151-919, Republic of Korea; [3]Department of Agricultural Biotechnology and Research Institute of Agriculture and Life Sciences, Seoul National University, Seoul 151-921, Republic of Korea; [4]College of Agriculture & Life Sciences, Chonnam National University, Gwangju, Republic of Korea; [5]Department of Biochemistry, Dongguk University College of Medicine, Gyeongju-si, Gyeongsangbuk-do 38066, Republic of Korea; [6]Department of Biotechnology, Catholic University of Daegu, Gyeongsan-si, Gyeongsangbuk-do 38430, Republic of Korea

Seong-Ryul Kim : ksr319@korea.kr; Woori Kwak : asleo@cnkgenomics.com; Hyaekang Kim : hkim458@snu.ac.kr; Kelsey Caetano-Anolles : kelseyca@gmail.com; Kee-Young Kim : applekky@korea.kr; Su-Bae Kim : subae@korea.kr; Kwang-Ho Choi : ckh@korea.kr; Seong-Wan; Seong-Wan Kim : tarupa@korea.kr; Jae-Sam Hwang : hwangjs@korea.kr; Min-Jae Kim : minjeekim3@gmail.com; Iksoo Kim : ikkim81@chonnam.ac.kr; Tae-Won Goo : gootw@dongguk.ac.kr

[†] These authors equally contributed and should be regarded as co-first authors.

* Corresponding authors

Seung-Won Park

Department of Biotechnology,

Catholic University of Daegu,

Gyeongsan-si, Gyeongsangbuk-do 38430, Republic of Korea

Phone : +82-53-850-3176

Fax : +82-53-359-6846

E-mail: microsw@cu.ac.kr

Abstract

Background

Antheraea yamamai, also known as the Japanese oak silk moth, is a wild species of silk moth. Silk produced by *A. yamamai*, referred to as *tensan* silk, shows different characteristics such as thickness, compressive elasticity and chemical resistance compared to the common silk produced from the domesticated silkworm, *Bombyx mori*. Its unique characteristics have led to its use in many research fields including biotechnology and medical science, and the scientific as well as economic importance of wild silk moth continues to gradually increase. However, no genomic information for wild silk moth, including *A. yamamai*, is currently available.

Findings

In order to construct the *A. yamamai* genome, a total of 147G base pairs using Illumina and Pacbio sequencing platforms were generated, providing 210-fold coverage based on the 700 Mb estimated genome size of *A. yamamai*. The assembled genome of *A. yamamai* was 656 Mb(>2kb) with 3,675 scaffolds and the N50 length of assembly was 739 Kb with 34.07% GC ratio. Identified repeat elements covered 37.33% of the total genome and the completeness of the constructed genome assembly was estimated to be 96.7% by BUSCO v2 analysis. A total of 21,124 genes were identified using Evidence Modeler based on the gene prediction results obtained from 3 different methods (*ab initio*, RNA-seq based, known-gene based).

Conclusions

Here we present the genome sequence of *A. yamamai*, the first genome sequence of wild silk moth. These results provide valuable genomic information which will help enrich our understanding of the molecular mechanisms related to not only specific phenotypes such as wild silk itself but also the genomic evolution of Saturniidae.

Keywords

Antheraea yamamai, Japanese silk moth, Japanese oak silk moth, wild silkworm

Data description

Antheraea yamamai (Figure 1), also known as the Japanese oak silk moth, is a wild silk moth species belonging to the Saturniidae family. Silk moths can be categorized into two families-Bombycidae and Saturniidae. Saterniidae has been estimated to contain approximately 1,861 species with 162 genera[1] and is known as the largest family in the Lepidoptera. Among the many species in family Saturniidae, only a few species, including *A. yamamai,* can be utilized for silk production. Previous phylogenetic studies have shown that family Saturniidae shares common ancestors with family Sphingidae, including the hawk moth (*Macroglossum stellatarum*) and Bombycidae family, including the most representative silkworm, *Bombyx mori* [2]. The estimated divergence time between *A. yamamai* and *B. mori* was 84 MYA(million years ago) and it was similar to 88 MYA, estimated divergence time between human and mouse[3, 4].

A. yamamai produces specific silk, called tensan silk[5], which shows distinctive characteristics compared to common silk from *B. mori*, such as characteristics such as thickness, bulkiness, compressive elasticity, and resistance to dyeing chemicals[6-8]. These characteristics receive the attention of researchers as a new biomaterial for use in various fields[9-11]. Additionally, it also has been studied for their applications to human health[12-15]. However, despite the importance of wild silk moth in research and economic fields, no whole genomic information is currently available for this wild silk moth or any other species from family Saturniidae.

In this study, we present the annotated genome sequence of *A. yamamai*, the first published genome in family Saturniidae, with transcriptome datasets collected from 10 different body organ tissues. This data will be a fundamental resource for future studies and provide more insight into the genome evolution and molecular phylogeny of family Saturniidae.

Sequencing

For whole genome sequencing, we selected one male sample(Ay-7-male1) from a breeding line (Ay-7) of *A. yamamai* raised at the National Academy of Agricultural Science, Rural Development Administration, Korea. In lepidopterans, males are homogametic(ZZ) and selecting male sample can reduce the complexity of assembly from excessive repeats on the W chromosome in females. For genomic library construction, we removed the guts of *A. yamamai* to prevent contamination of genomes from other organisms such as gut microbes and oak, the main food source of *A. yamamai*. Details of the sample preparation process used in this study are presented in the supplementary information. Genomic DNA was extracted using a DNeasy Animal Mini Kit (Qiagen, Hilden, Germany) and the quality of extracted DNA was checked using trenean, picogreen assay and gel electrophoresis (1% agarose gel/ 40ng loading). After quality control processing, we were left with a total of 61.5ug of *A. yamamai* DNA for genome sequencing. Using standard Illumina whole genome shotgun(WGS) sequencing protocol (paired-end and mate-pair), we added two long read sequencing platforms, Moleculo (Illumina synthetic long read) and RS II(Pacific Bioscience). Table 1-3 shows a summary of generated data for each library used in this study. RNA-seq libraries were also constructed for 10 different tissues (Hemocyte, Malpighian tube, Midgut, Fat Body, Anterior-Middle/Silk gland, Posterior/Silk gland, Head, Integument, Testis, Ovary) with 3 biological replicates following standard manufacturer protocol (Illumina, San Diego, CA, USA). For this, more than 100 individual *A. yamamai* samples in 5 instar stage from the same breeding line were used for tissue anatomy and 3 samples from each tissue were selected based on the quality of extracted RNA. Details of transcriptome library construction are shown in the supplementary information. Information of libraries and generated data is provided in Table 4, and a total of 147Gb of genomic data and 76Gb of transcriptomic data was generated for this study.

Genome assembly and evaluation

Before conducting genome assembly, we conducted k-mer distribution analysis using a 350bp paired-end library in order to estimate the size and characteristics of the *A. yamamai* genome. The quality of our generated raw data was checked using FASTQC[16](FastQC , RRID:SCR_014583). Sequencing artifacts such as adapter sequences and low-quality bases were removed using Trimmomatic[17]. Jellyfish[18] was used to count the k-mer frequency for estimation of the genome size of *A. yamamai*. Figure 2 shows the 19-mer distribution of *A. yamamai* genome using a 350bp paired-end library. In the 19-mer distribution, the second peak at approximately half the coverage value (x-axis) of the main peak indicates heterozygosity. Although the inbred line used in this study was the single pair sib-mating maintained for more than 10 generations, high heterozygosity still remains. This phenomenon has been observed in a previous genomic study of the Diamondback moth (*Plutella xylostella*), and sustained heterozygosity as an important genomic characteristic was hypothesized to be a result of environmental adaption[19]. Based on the result of 19-mer distribution analysis, the genome size of *A. yamamai* was estimated to be 709Mb. However, this size might be larger than the real genome size of *A.yamamai* because high heterozygosity could affect the estimation of genome size based on the K-mer distribution. Next, we conducted error correction on Illumina paired-end libraries using the error correction module of Allpaths-LG[20] before the initial contig assembly process (ALLPATHS-LG , RRID:SCR_010742). After error correction, initial contig assembly with 350bp and 700bp libraries was conducted using SOAP denovo2[21] with the parameter option set at K=19; this approach showed the best assembly statistics compared to other assemblers and parameters (SOAPdenovo2 , RRID:SCR_014986). Quality control processing for mate-pair libraries and scaffolding was conducted using Nxtrim[22] and

SSPACE (SSPACE , RRID:SCR_011848)[23], respectively. At each scaffolding step, SOAP Gapcloser[21] with -l 155 and -p 31 parameters was repeatedly used to close the gaps within each scaffold. In order to obtain a higher quality genome assembly of *A. yamamai*, we employed several long read scaffolding strategies using SSPACE-LongRead[24]. First, we used an Illumina synthetic long read sequencing platform called Moleculo which has been proven valuable for the study of highly heterozygous genomes in previous studies[25, 26]. After scaffolding was performed using SSPACE-LongRead with Illumina synthetic long read data, the total number of assembled scaffolds was effectively reduced from 398,446 to 24,558. The average scaffold length was also extended from 1.7 Kb to 24.8 Kb. However, there was no impressive improvement in N50 length (approximately 91 Kb to 112 Kb) of assembled scaffolds. Therefore, we employed another type of long read data generated from 10 cells of Pacbio RS II system with P6-C4 chemistry. After final scaffolding processing using Pacbio long reads, the number of scaffolds was reduced to 3,675 and N50 length was effectively extended from 112 Kb to 739 Kb. Summary statistics of the assembled *A. yamamai* genome is provided in Table 5. Final assembly of the *A. yamamai* genome was 656 Mb(>2kb) long with 3,675 scaffolds and the N50 length of assembly was 739 Kb with a 34.07% GC ratio. To evaluate the quality of the assembled genome, we conducted BUSCO (Benchmarking Universal Single-Copy Orthologs) analysis[27] using BUSCO v2.0 with insecta_odb9 including 1,658 BUSCOs from 42 species (BUSCO , RRID:SCR_015008). From BUSCO analysis, 96.7% of BUSCOs were completely detected in the assembled genome (1,576 : complete and single-copy, 27 : complete and duplicated) among 1,658 tested BUSCOs. The number of fragmented and missing BUSCOs was 21 and 34, respectively. Based on the result of BUSCO analysis, the genome of *A.yamamai* presented here was considered properly constructed for downstream analysis.

Repeat identification and comparative repeat analysis

To identify repeat elements of the *A. yamamai* genome, a custom repeat library was constructed using RepeatModeler with RECON[28], RepeatScout[29] and TRF[30]. The resulting constructed custom repeat library for *A. yamamai* was further curated using CENSOR[31] search and the curated library was employed in RepeatMasker[32] with Repbase[33]. RepeatMasker was conducted with RMBlast and 'no_is' option for skipping bacterial insertion element check. Table 6 summarizes the proportion of identified mobile elements in the A. *yamamai* genome. The most prevalent repeat elements in the *A. yamamai* genome were LINE element (101 Mb, 15.31% of total genome) and total repeat elements accounted for 37.33% of the total genome. In order to compare the repeat elements of *A. yamamai* with that of other genomes, we conducted the same process for seven public genomes which are close neighbors of *A. yamamai* - *Aedes aegypti[34]*, *Bombyx mori*[35], *Danaus plexippus*[36], *Drosophila melanogaster[37]*, *Heliconius melpomene*[38], *Melitaea cinxia*[39] and *Plutella xylostella*[19]. Figure 3 displays the amount and proportion of identified repeat elements from the 8 species. Despite the small genome size of *B. mori*, the total amount of identified SINE element in the *B. mori* genome was 5.77 times larger than that of *A. yamamai*. The top 5 expanded repeat elements in *A. yamamai* genome were DNA/RC, LINE/L2, LINE/RTE-BovB, DNA/TcMar-Mariner and LINE/CR1. Among these, DNA/TcMar-Mariner was the specifically expanded repeat element in *A. yamamai* among 8 species. In *B. mori*, SINE/tRNA-CR1, LINE/Jockey, DNA/RC, LINE/CR1-Zenon and LINE/RTE-BovB were the top 5 expanded repeat elements. When comparing the repeat elements of *A. yamamai* with those of *B. mori*, which are both producers of the same type of silk, repeat elements showed family and species-specific patterns in the two silk moth linages. Particularly, we found that the mariner repeat element, which was found specifically expanded in the *A. yamamai* genome, was also included in the fibroin gene.

A previous sequencing study also showed that the mariner repeat element was inserted in the 5'-end of fibroin gene of *A. yamamai*[40]. Fibroin is the core component of the silk protein found in silk moth, and the physical characteristics of silk mainly depend on the types and unique repeat motif of the fibroin[41]. This gene is known to have hundreds of tandem repeat motifs and these kinds of tandem repeats can be derived through transposable elements. This indicates that the mariner repeat element, specifically expanded in the *A. yamamai* genome, may play an important role in development of the unique silk of *A. yamamai*, and the lineage-specific repeat elements may be one of the candidate evolution forces related to host-specific phenotype during genome evolution.

Gene prediction and annotation

Three different algorithms were used for gene prediction of the *A. yamamai* genome: *ab initio*, RNA-seq transcript based, and protein homology-based approaches. For *ab initio* gene prediction, Augustus[42], Geneid[43] and GeneMarks-ET[44] were employed. Augustus was trained using known genes of *A. yamamai* in NCBI database and mapping information of RNA-seq data obtained from Tophat[45](TopHat , RRID:SCR_013035) was also utilized for gene prediction. Geneid was used with predefined parameters for *Drosophila melanogaster*. GeneMarks-ET was employed using junction information of genes from transcriptome data alignment. For RNA-seq transcript based prediction, generated transcriptome data from ten organ tissues of *A. yamamai* were aligned to the assembled genome and gene information was predicted using Cufflinks[46](Cufflinks , RRID:SCR_014597). The longest CDS sequences were identified from Cufflinks results using Transdecoder. For the homology-based approach, all known genes of order Lepidoptera in the NCBI database were aligned using PASA[47].

Table 7 shows the gene prediction results from each method. Gene prediction results from different prediction algorithms were combined using EVM (Evidence Modeler)[48] and a consensus gene set of the *A. yamamai* genome was created. Manual curation was performed based on the 5 evidences (3 in-silico, known protein and RNA-seq) using IGV[49] and Blastp. Using IGV with each gene evidence and comparing results with known genes via blastp, we mainly focused on the removing false positively predicted genes which don't have enough evidences. And merged and spliced gene structured were corrected by comparing the gene structure with known exon structure in NCBI NR database. In addition, fibroin and sericin genes which couldn't be properly predicted because of its high repeat motif were also manually identified with previously known sequences[40, 50] with RNA-seq data. The final gene set of *A. yamamai* genome contains 15,481 genes. Summary statistics for the consensus gene set is provided in Table 8. The average gene length was 11,016.34 bp with a 34.38% GC ratio and the number of exons per gene was 5.64. In order to identify the function of predicted genes in A yamamai, three non-redundant sequence databases (Swiss-Prot[51], Uniref100[51], and NCBI NR[52]) as well as the gene information of two species (*B. mori* and *D. melanogaster*) were used for target databases using Blastp. Additionally, protein domain searches were conducted on the consensus gene set using InterproScan5[53]. Figure S1 shows the top 20 identified terms from 7 different InterproScan5 analyses. Among the various analysis conducted using InterproScan5, gene ontology analysis with Pfam database showed that a large proportion of genes in the *A.yamamai* genome were related with the function of molecular binding, catalytic activity, internal component of membrane, metabolic process, oxidation-reduction process and transmembrane transport.

Comparative genome analysis

We used OrthoMCL[54] and RBH(Reciprocal Best Hit) within blastp for identification of gene family clusters and 1:1 orthologous gene sets. Gene information of 7 taxa (*A. aegypti, B. mori, D. plexippus, D. melanogaster, H. melpomene, M. cinxia* and *P. xylostella*), same taxa used in repeat analysis, was employed for OrthoMCL with *A. yamamai*. A total of 17,406 gene family clusters were constructed and 3,586 1:1 orthologous genes were identified. Before conducting comparative genome analysis, we constructed phylogenetic trees for the 8 species. In order to build the phylogenetic tree, multiple sequence alignment for the 1:1 orthologous genes of all 8 species was conducted using PRANK[55], and Gblocks[56] was used to obtain conserved blocks for the phylogenetic tree. Conserved block sequences were sequentially concatenated to obtain one consensus sequence for each species. MEGA[57] was used for constructing Neighbor-Joining Trees (bootstrap 1000, maximum composite likelihood, transitions + transversions, and gamma distributed option) and MrBayes[58] was employed for the construction of Bayesian inference trees. To select the best evolution model for our data, Modeltest[59] was conducted and the GTR based invariant model was chosen based on the AIC value of Modeltest. Figure 4 shows the constructed phylogenetic tree of the 8 species using 3,586 orthologous genes. The bootstrap value and Bayesian poster probability value of all nodes were 100 and 1, respectively. The closest neighbor of *A. yamamai* was *B. mori*, which is included in Bombycidae family; this result is consistent with that of previous studies. Three butterfly species (*D. plexippus, M. cinxia and H. meplmene*) included in Nymphalidae family were also shown to share a common ancestor with families Saturniidae and Bombycidae.

Based on the constructed phylogenetic tree, gene family expansion and contraction analysis was conducted using a 2 parameter model in CAFE[60] and the gene tree was constructed using protein sequence via MEGA[57]. Figure 4 shows the result of gene family expansion and contraction analysis of 8 species. 938 and 1,987 gene families of *A. yamamai* and 567 and 715 gene families of *B. mori* were estimated to be expanded and contracted from the common

ancestors, respectively. Among these, 15 gene families in *A. yamamai* were estimated to be under rapid expansion during the evolution process. Functions of genes in rapidly expanded gene families of *A. yamamai* were transposase, fatty acid synthase, zinc finger protein, chorion (eggshell protein), reverse transcriptase, prostaglandin dehydrogenase, RNA-directed DNA polymerase, gag like protein, juvenile hormone acid methyltransferase, facilitated trehalose transporter and glucose dehydrogenase. Figure 5 shows the gene tree of two chorion gene (chorion class A and B) family clusters rapidly expanded in the *A. yamamai* genome. Chorion, called eggshell protein, composes the surface of egg and protects the embryo from environmental threats such as desiccation, flooding, freezing, infection of microorganisms, and physical destruction. It also provides channels, such as aeropyle, which enables gas exchange and maintains proper condition for diapause egg[61]. These diverse functions of eggshell are implemented by the specific eggshell structure and the surface structure of eggshell varies between species for the adaptation in a different environment. The ancestor of Antherea has the unique aeropyle structure called "aerophyle crown" on the eggshell surface[62]. This unique structure is formed by the circular vertical projection of lamellar chorion from follicle cell and it surrounds the aeropyles near the end of oogenesis[63]. Acquiring this kind of *de novo* complex structure requires numerous genetic changes and a previous study about *Antheraea Polyphemus* has shown that over a hundred chorion specific polypeptides were involved for this unique ultra-structure[63]. Therefore, the specific rapid expansion of chorion class A and B gene family in *A. yamamai* genome might be one of the convincing molecular explanation for acquiring this unique ultrastructure in the eggshell surface of Antheraea genus. However, this unique ultra-structure tends to be reduced during current evolution process of the Antheraea genus. Types of eggshell structure in Antheraea genus can be categorized into multiple classes based on the morphology and regional distribution of aeropyle[62]. The shape of aeropyle in *A. yamamai* egg is known to be converted to mound shape from the crown shape

and these aeropyle mounds only exist in the narrow band surrounding the micropyle region[62]. Only a very few, small aeropyle crowns remained and it is entirely different with the ancestral form of eggshell surface mostly covered by aeropyle crowns. These regional differences were known to be adjusted by regional difference of filler genes during choriogenesis[64] and the additional regulations of related genes for choriogenesis have to be considered. This indicates that specifically expanded chorion gene families of *A. yamamai* may be one of the remaining evolutionary tracks in the genome of Antheraea genus. However, further functional studies must be conducted to resolve the limited understanding about the relationship between these expanded chorion gene families and the current eggshell surface formation of *A. yamamai.*

The constructed genome of *A.yamamai* presented here is the first announced genome in family Saturniidae and the karyotyping analysis using gamete in metaphase showed that the genome of *A. yamamai* consists of 31 chromosomes (Figure 6). This constructed genome information provides more insight into the genome evolution and phylogeny of family Saturniidae, which contains the largest number of species in Lepidoptera. For example, although two silk moths, *A. yamamai* and *B. mori,* appear similar, comparative genome analysis showed the significant differences in the genome size, specific expansion of repeat elements and gene families between families Saturniidae and Bombycidae. In case of molecular phylogeny, most previous phylogenetic studies were limited to few genes due to the lack of genomic information on family Saturniidae. We expect our study and resulting constructed genome will resolve some limitations of molecular phylogenetic and ecological research on Saturniidae species. Additionally, constructed genome information will help researchers better understand the molecular background of wild silk and its production. Silk produced by *A. yamamai,* referred to as *tensan* silk, shows unique characteristics which have made it valuable in various fields. However, *A. yamam*ai has not been completely domesticated compared to *B. mori*, making mass production of tensan silk infeasible. Understanding of the molecular mechanisms behind

the tensan silk production process is essential for mass production using biotechnology, and this genome sequence with manually curated gene information is a fundamental resource for related research and industrial improvement. Additionally, the transcriptome data of 10 different organ tissues with 3 biological replications presented here may be also useful resources for uncovering the molecular mechanisms related to specific phenotypes of *A.yamamai* and family Saturniidae.

Availability of supporting data

The generated genome sequence and gene information of *A. yamamai* are available in GigaDB[65] and generated raw data is available under project accession PRJNA383008 and PRJNA383025 of the NCBI database.

Competing interests

All authors report no competing interests.

Abbreviation

RBH – Reciprocal Best Hit

Authors contributions

Sampling - Kee-Young Kim, Su-Bae Kim

Sequencing - Kwang-Ho Choi, Seong-Wan Kim

Genome assembly - Seong-Ryul Kim, Woori Kwak, Jae-Sam Hwang, Seung-Won Park

Repeat element analysis - Seong-Ryul Kim, Woori Kwak, Seung-Won Park

Gene prediction - Seong-Ryul Kim, Woori Kwak, Hyaekang Kim, Jae-Sam Hwang

Comparative genome analysis - Seong-Ryul Kim, Woori Kwak, Min-Jae Kim, Kelsey Caetano-Anolles

Funding and experimental design - Seong-Ryul Kim, Seung-Won Park

Acknowledgements

This work was supported by a grant from the Rural Development Administration, Republic of Korea (grant no. PJ010442).

References

1. Regier, J.C., M.C. Grant, C. Mitter, et al., *Phylogenetic relationships of wild silkmoths (Lepidoptera: Saturniidae) inferred from four protein-coding nuclear genes.* Systematic Entomology, 2008. **33**(2): p. 219-228.
2. Regier, J.C., C. Mitter, A. Zwick, et al., *A large-scale, higher-level, molecular phylogenetic study of the insect order Lepidoptera (moths and butterflies).* PLoS One, 2013. **8**(3): p. e58568.
3. Hedges, S.B., J. Dudley, and S. Kumar, *TimeTree: a public knowledge-base of divergence times among organisms.* Bioinformatics, 2006. **22**(23): p. 2971-2972.
4. Kawahara, A.Y. and J.R. Barber, *Tempo and mode of antibat ultrasound production and sonar jamming in the diverse hawkmoth radiation.* Proceedings of the National Academy of Sciences, 2015. **112**(20): p. 6407-6412.
5. Peigler, R.S., *Wild silks of the world.* American Entomologist, 1993. **39**(3): p. 151-162.
6. MATSUMOTO, Y.-I. and H. SAITO, *Load-extension characteristics of composite raw silk of Antheraea yamamai and Bombyx mori.* The Journal of Sericultural Science of Japan, 1997. **66**(6): p. 497-501.
7. Nakamura, S., Y. Saegusa, Y. Yamaguchi, et al., *Physical properties and structure of silk. XI. Glass transition temperature of wild silk fibroins.* Journal of applied polymer science, 1986. **31**(3): p. 955-956.
8. Kweon, H. and Y. Park, *Structural characteristics and physical properties of wild silk fibres; Antheraea pernyi and Antheraea yamamai.* Korean Journal of Sericultural Science (Korea Republic), 1994.
9. Zheng, Z., Y. Wei, S. Yan, et al., *Preparation of regenerated Antheraea yamamai silk fibroin film and controlled-molecular conformation changes by aqueous ethanol treatment.* Journal of applied polymer science, 2010. **116**(1): p. 461-467.
10. Omenetto, F., D. Kaplan, J. Amsden, et al., *Silk based biophotonic sensors*. 2011, Google Patents.
11. Takeda, S., *New field of insect science: Research on the use of insect properties.* Entomological Science, 2013. **16**(2): p. 125-135.
12. Omenetto, F. and D.L. Kaplan, *Silk-based multifunctional biomedical platform*. 2012, Google Patents.
13. Serban, M.A., *Silk medical devices*. 2016, Google Patents.
14. Jiang, G.-L., A.L. Collette, R.L. Horan, et al., *Drug delivery platforms comprising silk fibroin hydrogels and uses thereof.* 2010, Google Patents.
15. Kamiya, M., K. Oyauchi, Y. Sato, et al., *Structure–activity relationship of a novel pentapeptide with cancer cell growth-inhibitory activity.* Journal of Peptide Science, 2010. **16**(5): p. 242-248.
16. Bioinformatics, B., *FastQC A quality control tool for high throughput sequence data.*

Cambridge, UK: Babraham Institute, 2011.

17. Bolger, A.M., M. Lohse, and B. Usadel, *Trimmomatic: a flexible trimmer for Illumina sequence data.* Bioinformatics, 2014: p. btu170.

18. Marçais, G. and C. Kingsford, *A fast, lock-free approach for efficient parallel counting of occurrences of k-mers.* Bioinformatics, 2011. **27**(6): p. 764-770.

19. You, M., Z. Yue, W. He, et al., *A heterozygous moth genome provides insights into herbivory and detoxification.* Nature genetics, 2013. **45**(2): p. 220-225.

20. Gnerre, S., I. MacCallum, D. Przybylski, et al., *High-quality draft assemblies of mammalian genomes from massively parallel sequence data.* Proceedings of the National Academy of Sciences, 2011. **108**(4): p. 1513-1518.

21. Luo, R., B. Liu, Y. Xie, et al., *SOAPdenovo2: an empirically improved memory-efficient short-read de novo assembler.* Gigascience, 2012. **1**(1): p. 18.

22. O'Connell, J., O. Schulz-Trieglaff, E. Carlson, et al., *NxTrim: optimized trimming of Illumina mate pair reads.* Bioinformatics, 2015. **31**(12): p. 2035-2037.

23. Boetzer, M., C.V. Henkel, H.J. Jansen, et al., *Scaffolding pre-assembled contigs using SSPACE.* Bioinformatics, 2011. **27**(4): p. 578-579.

24. Boetzer, M. and W. Pirovano, *SSPACE-LongRead: scaffolding bacterial draft genomes using long read sequence information.* BMC bioinformatics, 2014. **15**(1): p. 211.

25. Voskoboynik, A., N.F. Neff, D. Sahoo, et al., *The genome sequence of the colonial chordate, Botryllus schlosseri.* Elife, 2013. **2**: p. e00569.

26. McCoy, R.C., R.W. Taylor, T.A. Blauwkamp, et al., *Illumina TruSeq synthetic long-reads empower de novo assembly and resolve complex, highly-repetitive transposable elements.* PloS one, 2014. **9**(9): p. e106689.

27. Simão, F.A., R.M. Waterhouse, P. Ioannidis, et al., *BUSCO: assessing genome assembly and annotation completeness with single-copy orthologs.* Bioinformatics, 2015: p. btv351.

28. Bao, Z. and S.R. Eddy, *Automated de novo identification of repeat sequence families in sequenced genomes.* Genome Research, 2002. **12**(8): p. 1269-1276.

29. Price, A.L., N.C. Jones, and P.A. Pevzner, *De novo identification of repeat families in large genomes.* Bioinformatics, 2005. **21**(suppl 1): p. i351-i358.

30. Benson, G., *Tandem repeats finder: a program to analyze DNA sequences.* Nucleic acids research, 1999. **27**(2): p. 573.

31. Kohany, O., A.J. Gentles, L. Hankus, et al., *Annotation, submission and screening of repetitive elements in Repbase: RepbaseSubmitter and Censor.* BMC bioinformatics, 2006. **7**(1): p. 474.

32. Tarailo-Graovac, M. and N. Chen, *Using RepeatMasker to identify repetitive elements in genomic sequences.* Current Protocols in Bioinformatics, 2009: p. 4.10. 1-4.10. 14.

33. Bao, W., K.K. Kojima, and O. Kohany, *Repbase Update, a database of repetitive elements in eukaryotic genomes.* Mobile DNA, 2015. **6**(1): p. 11.

34. Nene, V., J.R. Wortman, D. Lawson, et al., *Genome sequence of Aedes aegypti, a major*

arbovirus vector. Science, 2007. **316**(5832): p. 1718-1723.
35. Xia, Q., Z. Zhou, C. Lu, et al., *A draft sequence for the genome of the domesticated silkworm (Bombyx mori).* Science, 2004. **306**(5703): p. 1937-1940.
36. Zhan, S., C. Merlin, J.L. Boore, et al., *The monarch butterfly genome yields insights into long-distance migration.* Cell, 2011. **147**(5): p. 1171-1185.
37. Adams, M.D., S.E. Celniker, R.A. Holt, et al., *The genome sequence of Drosophila melanogaster.* Science, 2000. **287**(5461): p. 2185-2195.
38. Consortium, H.G., *Butterfly genome reveals promiscuous exchange of mimicry adaptations among species.* Nature, 2012. **487**(7405): p. 94-98.
39. Ahola, V., R. Lehtonen, P. Somervuo, et al., *The Glanville fritillary genome retains an ancient karyotype and reveals selective chromosomal fusions in Lepidoptera.* Nature communications, 2014. **5**.
40. Hwang, J.-S., J.-S. Lee, T.-W. Goo, et al., *Cloning of the fibroin gene from the oak silkworm, Antheraea yamamai and its complete sequence.* Biotechnology letters, 2001. **23**(16): p. 1321-1326.
41. Malay, A.D., R. Sato, K. Yazawa, et al., *Relationships between physical properties and sequence in silkworm silks.* Scientific reports, 2016. **6**: p. 27573.
42. Stanke, M., M. Diekhans, R. Baertsch, et al., *Using native and syntenically mapped cDNA alignments to improve de novo gene finding.* Bioinformatics, 2008. **24**(5): p. 637-644.
43. Blanco, E., G. Parra, and R. Guigó, *Using geneid to identify genes.* Current protocols in bioinformatics, 2007: p. 4.3. 1-4.3. 28.
44. Lomsadze, A., P.D. Burns, and M. Borodovsky, *Integration of mapped RNA-Seq reads into automatic training of eukaryotic gene finding algorithm.* Nucleic acids research, 2014: p. gku557.
45. Trapnell, C., L. Pachter, and S.L. Salzberg, *TopHat: discovering splice junctions with RNA-Seq.* Bioinformatics, 2009. **25**(9): p. 1105-1111.
46. Trapnell, C., A. Roberts, L. Goff, et al., *Differential gene and transcript expression analysis of RNA-seq experiments with TopHat and Cufflinks.* Nature protocols, 2012. **7**(3): p. 562-578.
47. Campbell, M.A., B.J. Haas, J.P. Hamilton, et al., *Comprehensive analysis of alternative splicing in rice and comparative analyses with Arabidopsis.* BMC genomics, 2006. **7**(1): p. 327.
48. Haas, B.J., S.L. Salzberg, W. Zhu, et al., *Automated eukaryotic gene structure annotation using EVidenceModeler and the Program to Assemble Spliced Alignments.* Genome biology, 2008. **9**(1): p. R7.
49. Robinson, J.T., H. Thorvaldsdóttir, W. Winckler, et al., *Integrative genomics viewer.* Nature biotechnology, 2011. **29**(1): p. 24-26.
50. Zurovec, M., N. Yonemura, B. Kludkiewicz, et al., *Sericin Composition in the Silk of Antheraea yamamai.* Biomacromolecules, 2016. **17**(5): p. 1776-1787.
51. Consortium, U., *Reorganizing the protein space at the Universal Protein Resource*

(UniProt). Nucleic acids research, 2011: p. gkr981.

52. Pruitt, K.D., T. Tatusova, and D.R. Maglott, *NCBI reference sequences (RefSeq): a curated non-redundant sequence database of genomes, transcripts and proteins.* Nucleic acids research, 2007. **35**(suppl 1): p. D61-D65.
53. Jones, P., D. Binns, H.-Y. Chang, et al., *InterProScan 5: genome-scale protein function classification.* Bioinformatics, 2014. **30**(9): p. 1236-1240.
54. Li, L., C.J. Stoeckert, and D.S. Roos, *OrthoMCL: identification of ortholog groups for eukaryotic genomes.* Genome research, 2003. **13**(9): p. 2178-2189.
55. Löytynoja, A. and N. Goldman, *An algorithm for progressive multiple alignment of sequences with insertions.* Proceedings of the National Academy of Sciences of the United States of America, 2005. **102**(30): p. 10557.
56. Castresana, J., *Selection of conserved blocks from multiple alignments for their use in phylogenetic analysis.* Molecular biology and evolution, 2000. **17**(4): p. 540-552.
57. Kumar, S., G. Stecher, and K. Tamura, *MEGA7: Molecular Evolutionary Genetics Analysis version 7.0 for bigger datasets.* Molecular biology and evolution, 2016. **33**(7): p. 1870-1874.
58. Ronquist, F. and J.P. Huelsenbeck, *MrBayes 3: Bayesian phylogenetic inference under mixed models.* Bioinformatics, 2003. **19**(12): p. 1572-1574.
59. Posada, D., *Using MODELTEST and PAUP* to select a model of nucleotide substitution.* Current protocols in bioinformatics, 2003: p. 6.5. 1-6.5. 14.
60. De Bie, T., N. Cristianini, J.P. Demuth, et al., *CAFE: a computational tool for the study of gene family evolution.* Bioinformatics, 2006. **22**(10): p. 1269-1271.
61. Chapman, R.F., *The insects: structure and function*. 1998: Cambridge university press.
62. Regier, J.C., U. Paukstadt, L.H. Paukstadt, et al., *Phylogenetics of eggshell morphogenesis in Antheraea (Lepidoptera: Saturniidae): unique origin and repeated reduction of the aeropyle crown.* Systematic biology, 2005. **54**(2): p. 254-267.
63. Regier, J.C., G.D. Mazur, and F.C. Kafatos, *The silkmoth chorion: morphological and biochemical characterization of four surface regions.* Developmental biology, 1980. **76**(2): p. 286-304.
64. Hatzopoulos, A.K. and J.C. Regier, *Evolutionary changes in the developmental expression of silkmoth chorion genes and their morphological consequences.* Proceedings of the National Academy of Sciences, 1987. **84**(2): p. 479-483.
65. Sneddon, T.P., P. Li, and S.C. Edmunds, *GigaDB: announcing the GigaScience database.* GigaScience, 2012. **1**(1): p. 11.

Tables

Table 1. Summary statistics of generated whole genome shotgun sequencing data using Illumina Nextseq 500.

Library Name	Library Type	Insert Size	Platform	Read Length	No. Reads	Total Base(bp)	Reads retained after trimming
350bp	Paired-end	350bp	Nextseq500	151	293,176,268	44,269,616,468	291,070,362
700bp	Paired-end	700bp	Nextseq500	151	246,945,900	37,288,830,900	244,698,580
3Kbp	Mate-pair	3Kbp	Nextseq500	76	284,204,762	21,599,561,912	195,095,164
6Kbp	Mate-pair	6Kbp	Nextseq500	76	246,238,370	18,714,116,120	152,496,372
9Kbp	Mate-pair	9Kbp	Nextseq500	76	239,919,538	18,233,884,888	148,612,724
**Total**					1,310,484,838	140,106,010,288	1,031,973,202

Table 2. Summary statistics of generated Illumina synthetic long read (Moleculo) library.

	500-1499bp	>= 1500bp
Number of assembled read	302,132	342,738
Number of bases in assembled read	268,853,717	1,205,349,082
N50 length of assembled read	960	4,031

Table 3. Summary statistics of generated long reads data using Pacbio RS II system.

Number of Reads	1,005,571
Total Bases	5,836,969,225
Length of longest (shortest) read	50,132(50)
Average read length	5,804.63

Table 4. Summary statistics of generated transcriptome data obtained from six organ tissues using Illumina platform.

Tissue	Sample Name	Read Length	Read Count	Total Base (bp)
Hemocyte	Hemocyte_1	76	20,815,674	1,581,991,224
	Hemocyte_2	76	26,704,666	2,029,554,616
	Hemocyte_2	76	53,068,562	4,033,210,712
Malpighian Tube	Malpighi_1	76	22,635,428	1,720,292,528
	Malpighi_2	76	24,893,788	1,891,927,888
	Malpighi_3	76	45,213,164	3,436,200,464
Midgut	Midgut_1	76	23,350,138	1,774,610,488
	Midgut_2	76	24,597,972	1,869,445,872
	Midgut_3	76	50,949,986	3,872,198,936
Head	Head_1	76	26,526,276	2,015,996,976
	Head_2	76	26,581,124	2,020,165,424
	Head_3	76	40,900,456	3,108,434,656
Integument	Skin_1	76	24,592,846	1,869,056,296
	Skin_2	76	42,775,430	3,250,932,680
	Skin_3	76	35,043,570	2,663,311,320
Fat Body	Fat Body_1	76	24,637,810	1,872,473,560
	Fat Body_2	76	24,037,494	1,826,849,544
	Fat Body_3	76	40,817,582	3,102,136,232
Anterior-Middle/Silk Gland	AM/Silk Gland_1	76	21,399,638	1,626,372,488
	AM/Silk Gland_2	76	24,292,386	1,846,221,336
	AM/Silk Gland_3	76	37,331,530	2,837,196,280
Posterior/Silk Gland	P/Silk Gland_1	76	27,359,580	2,079,328,080
	P/Silk Gland_2	76	23,300,962	1,770,873,112
	P/Silk Gland_3	76	39,421,430	2,996,028,680
Testis	Testis_1	76	40,890,404	3,107,670,704
	Testis_2	76	45,733,846	3,475,772,296
	Testis_3	76	44,985,224	3,418,877,024
Ovary	Ovary_1	76	40,797,628	3,100,619,728
	Ovary_2	76	40,409,752	3,071,141,152
	Ovary_3	76	42,417,892	3,223,759,792

Table 5. Summary statistics of the *A. yamamai* genome (>2kb).

**Assembled Genome**	
Size(1n)	656 Mb
GC level	34.07
No. scaffolds	3,675
N50 of scaffolds (bp)	739,388
N bases in scaffolds (%)	19,257,439 (2.93)
Longest(shortest) scaffolds (bp)	3,156,949 (2,003)
Average scaffold Length (bp)	178,657.53

Table 6. Summary of identified repeat elements in the *A. yamamai* genome.

**Repeat Element**	No. Element	Length (%)
SINE	59,968	8,615,338(1.30)
LINE	426,522	101,251,176(15.31)
LTR element	53,977	4,552,386(0.69)
DNA element	512,760	69,071,227(10.44)
Small RNA	43,645	6,691,619(1.01)
Simple repeat	135,989	6,256,839(0.95)
Low complexity	19,937	932,829(0.14)
Unclassified	294,190	54,552,009(8.25)

Table 7. Summary statistics of ab initio, RNA-seq based and homology-based gene prediction results.

Evidence Type	Programs	Element	Total count	Exon/Gene	Total length(bp)	Mean length(bp)
ab_initio	Augustus	Gene	14,576	4.85	142,415,318	9,770.53
		Exon	70,733		14,736,668	208.34
	Geneid	Gene	10,946	2.25	46,119,402	4,213.35
		Exon	24,686		3,925,563	159.01
	GeneMarks-ET	Gene	27,754	5.50	273,745,951	9,863.29
		Exon	152,660		30,847,503	202.06
RNA-seq	Cufflinks Transdecoder	Gene	36,213	7.03	840,429,061	23,207.94
		Exon	254,770		201,721,675	791.77
Known Gene (NCBI lepidoptera)	PASA (gmap)		44,561		22,484,151	504.57

Table 8. Summary statistics for the consensus gene set of the *A. yamamai* genome.

Element	No. elements	Exon/Gene	Avg. length	Total length	Genome coverage(%)
Gene	15,481	5.64	11,016.34	170,543,958	25.78
Exon	87,346		1,346.23	20,840,925	3.31

Figures

Figure 1. Photograph of *Antheraea Yamamai*. From left- larva, cocoon and adult *A. yamamai*, respectively. Green color is one of the representative characteristics of tensan silk.

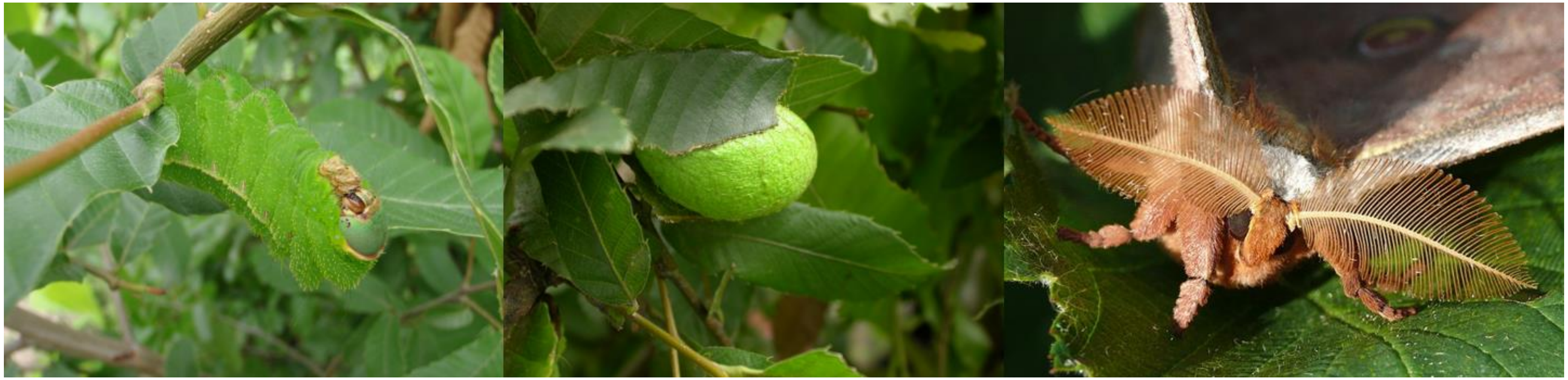

Figure 2. 19-mer distribution of *A. yamamai* genome using jellyfish with 350bp paired-end whole genome sequencing data.

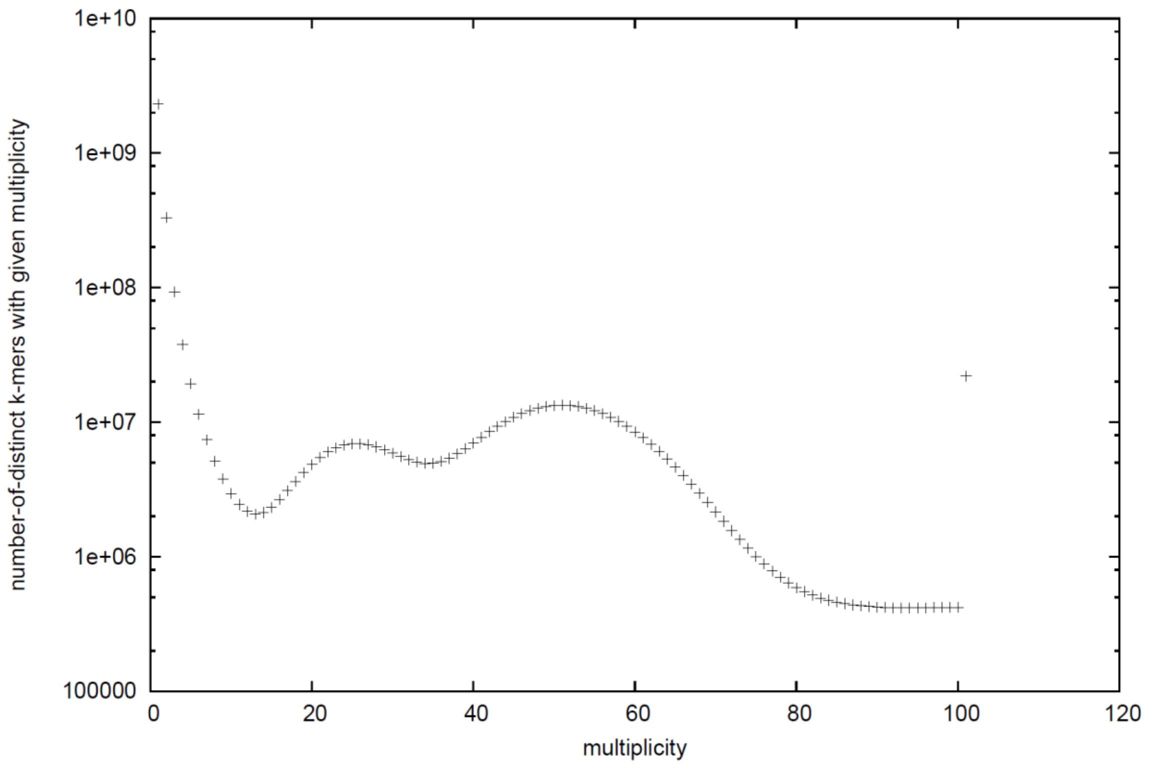

Figure 3. Amount and proportion of identified repeat element from 8 species including *A. yamamai*. a. Absolute amount of repeat element classified into 8 different categories. b. Proportion of each repeat element in identified total repeat element.

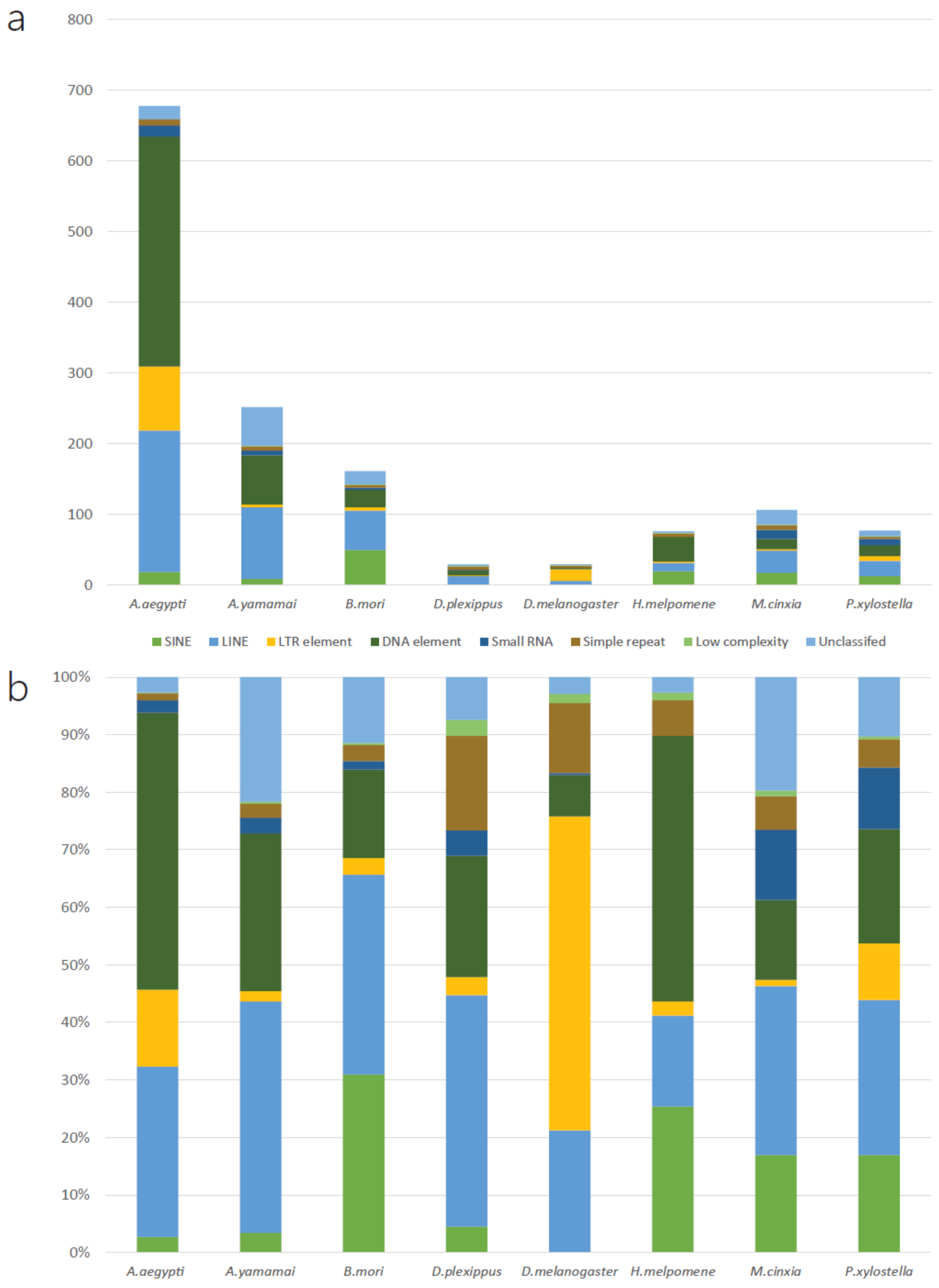

Figure 4. Constructed phylogenetic tree and comparative gene family analysis. Nodes value indicate Bayesian posterior probability, bootstrap and gene expansion, contraction value. Orange and blue color indicate expansion and contraction, respectively. Bar chart indicate the number of genes cauterized into 4 groups (Specific, 1:Multi, Multi:Multi and 1:1) using OrthoMCL.

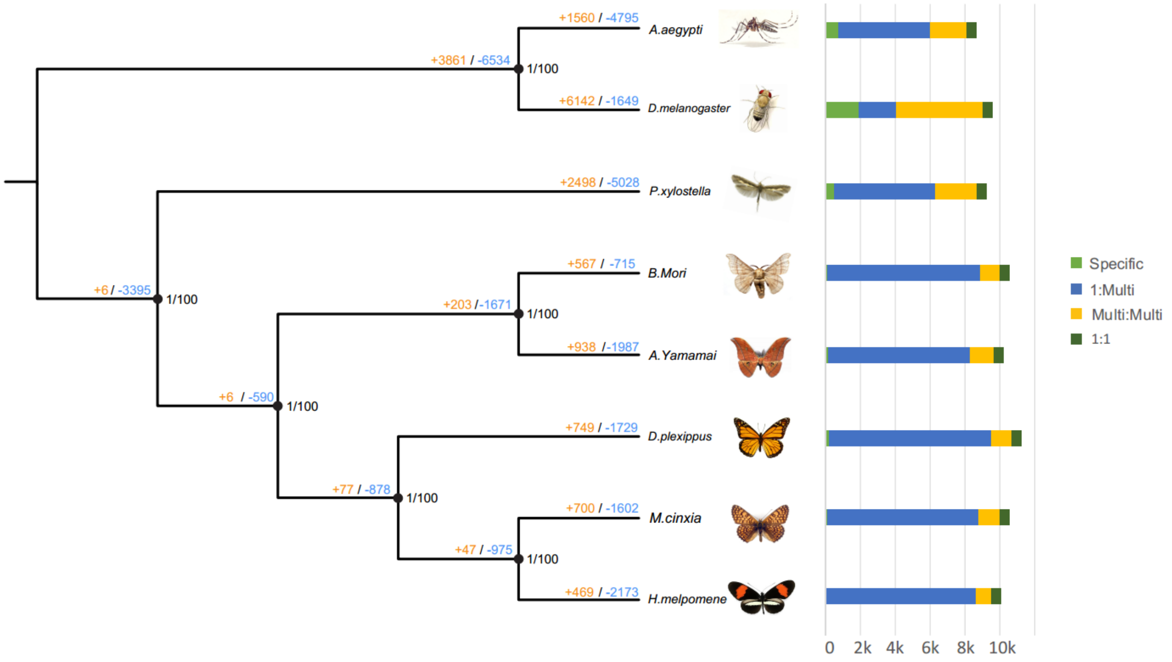

Figure 5. Expansion of chorion gene in *A.yamamai* genome. a and b shows the gene tree of chorion A and B in the rapid expanded gene family cluster, respectively. Color of terminal node indicates each taxon identified in the gene family cluster.

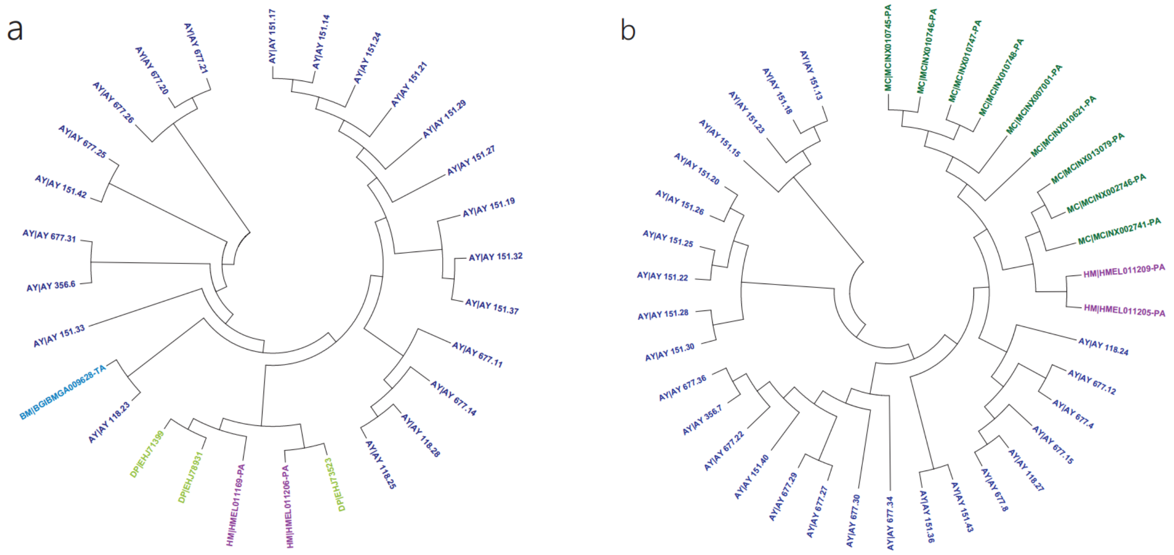

Figure 6. Karyotype of *A.yamamai* using a gamete of testis in metaphase.

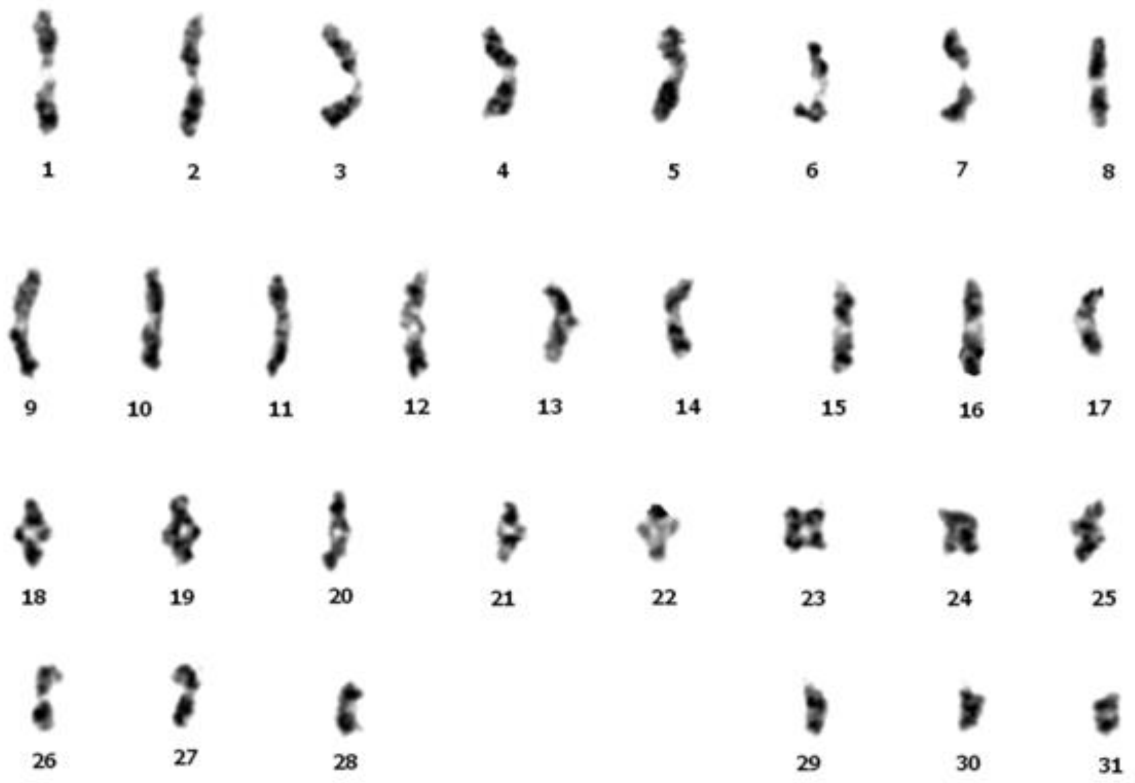

Figure 1

Click here to download Figure Figure1.png

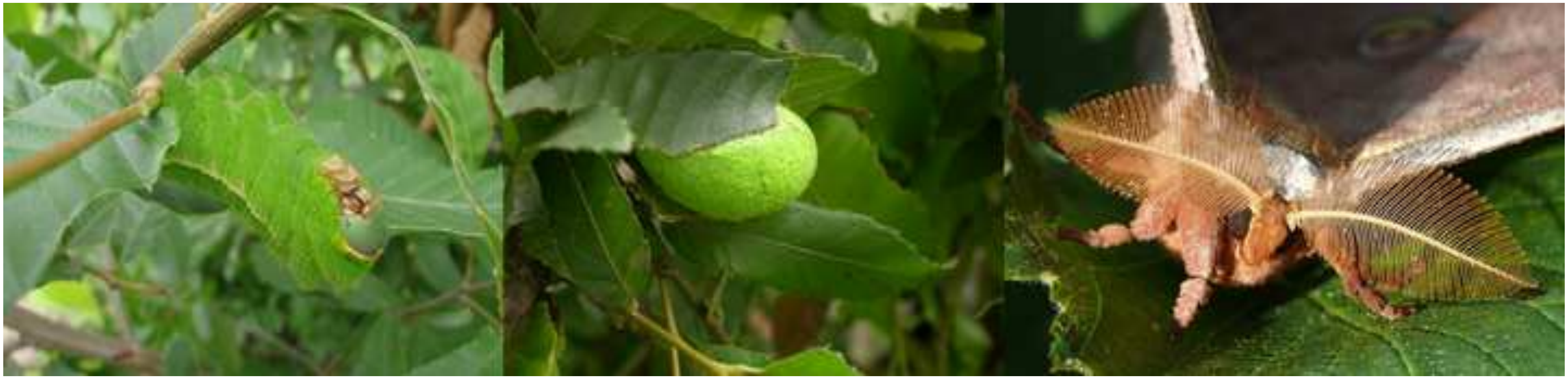

Figure 2

Click here to download Figure Figure2.pdf

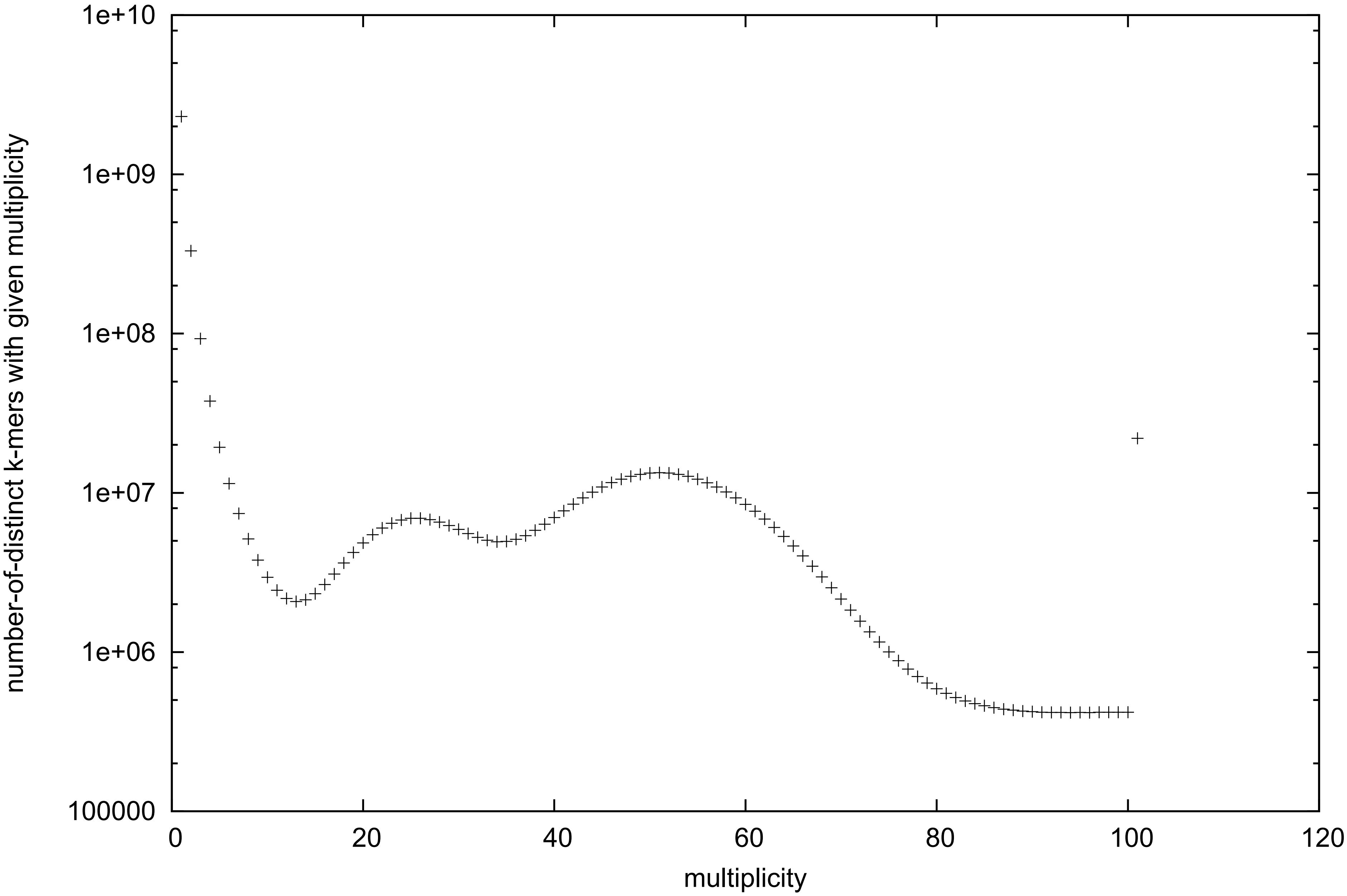

Figure 3

Click here to download Figure Figure3.pdf

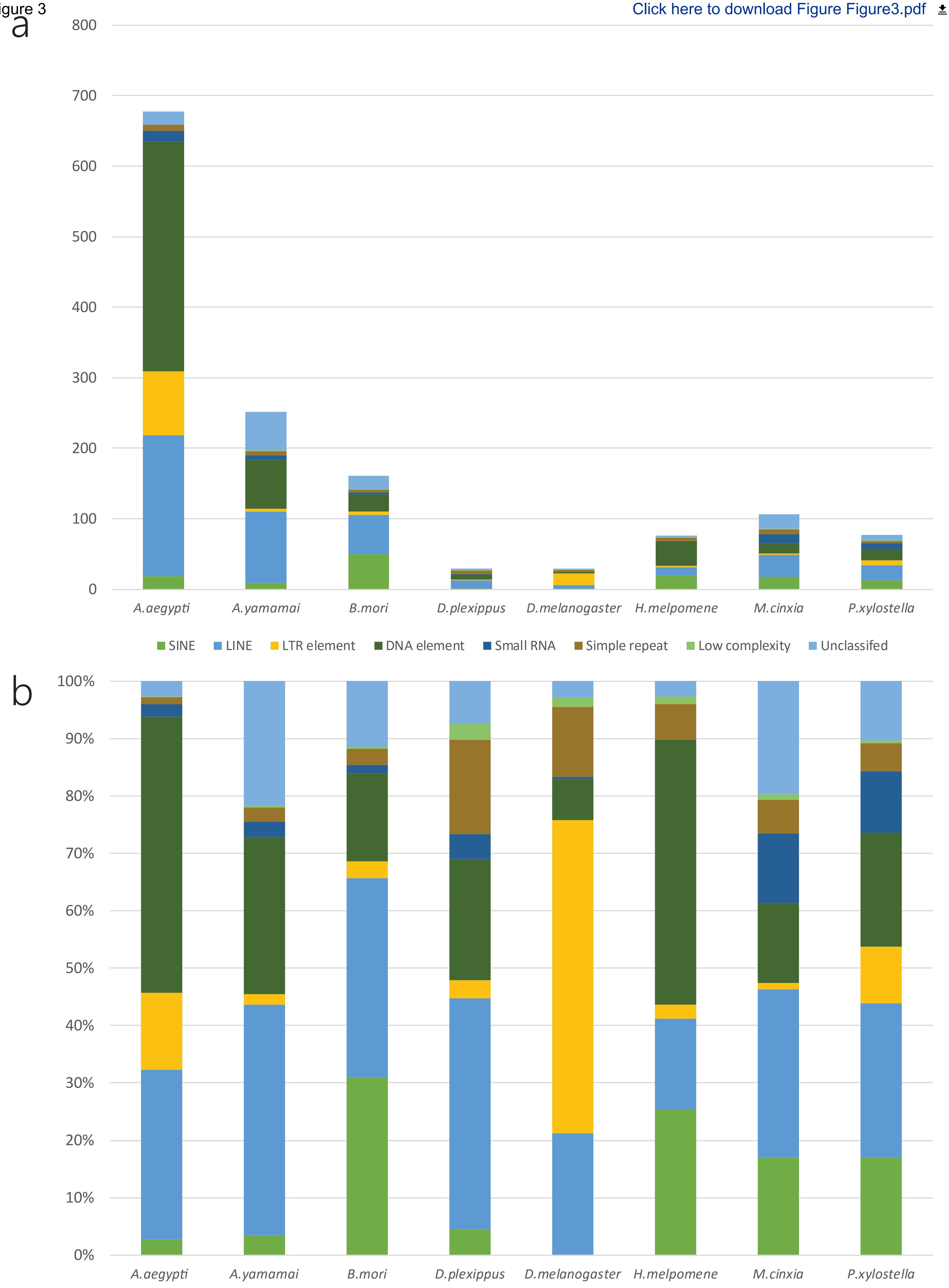

Click here to download Figure Figure4.pdf

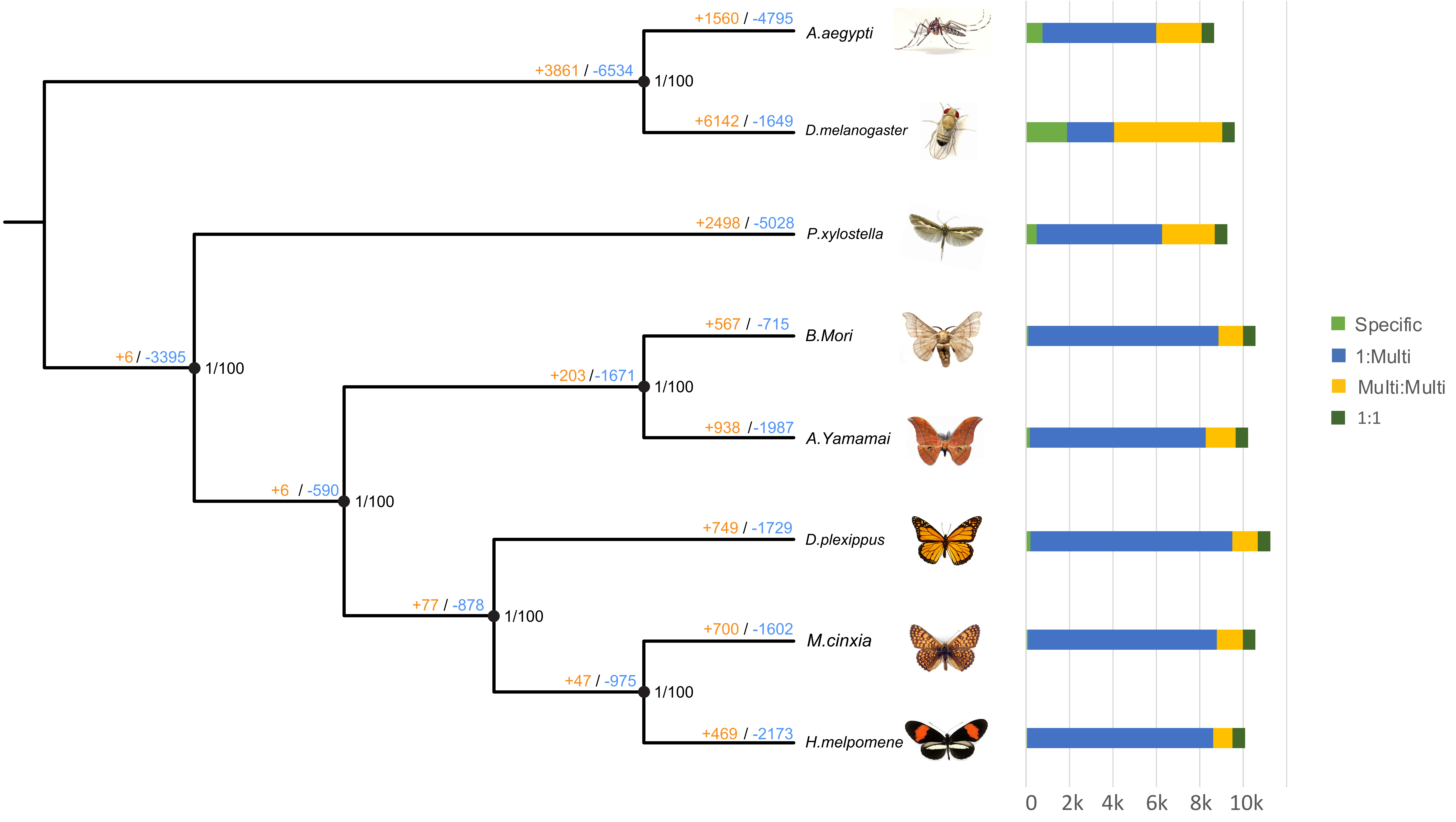

Figure 5

Click here to download Figure Figure5.pdf

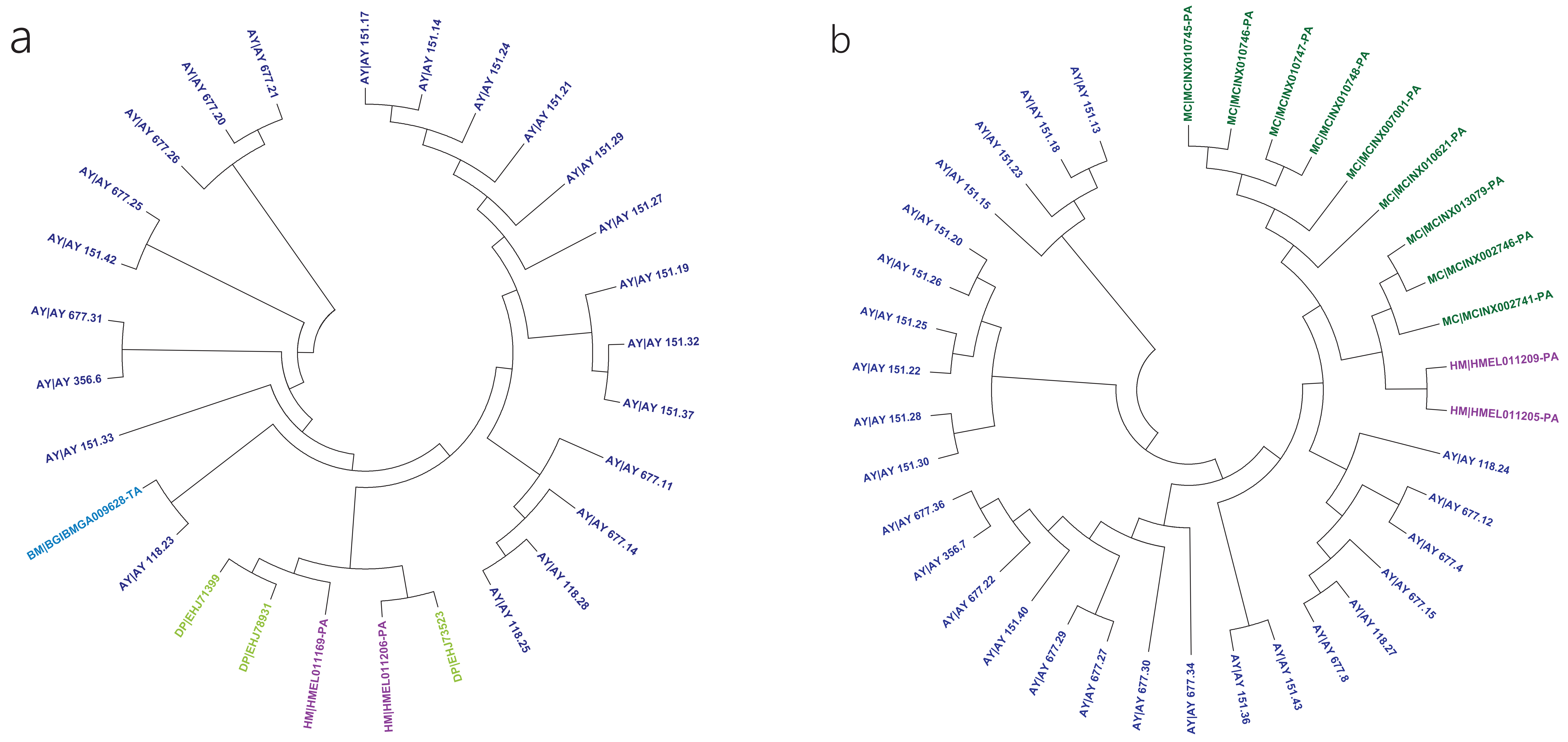

Figure 6

Click here to download Figure Figure6.jpg

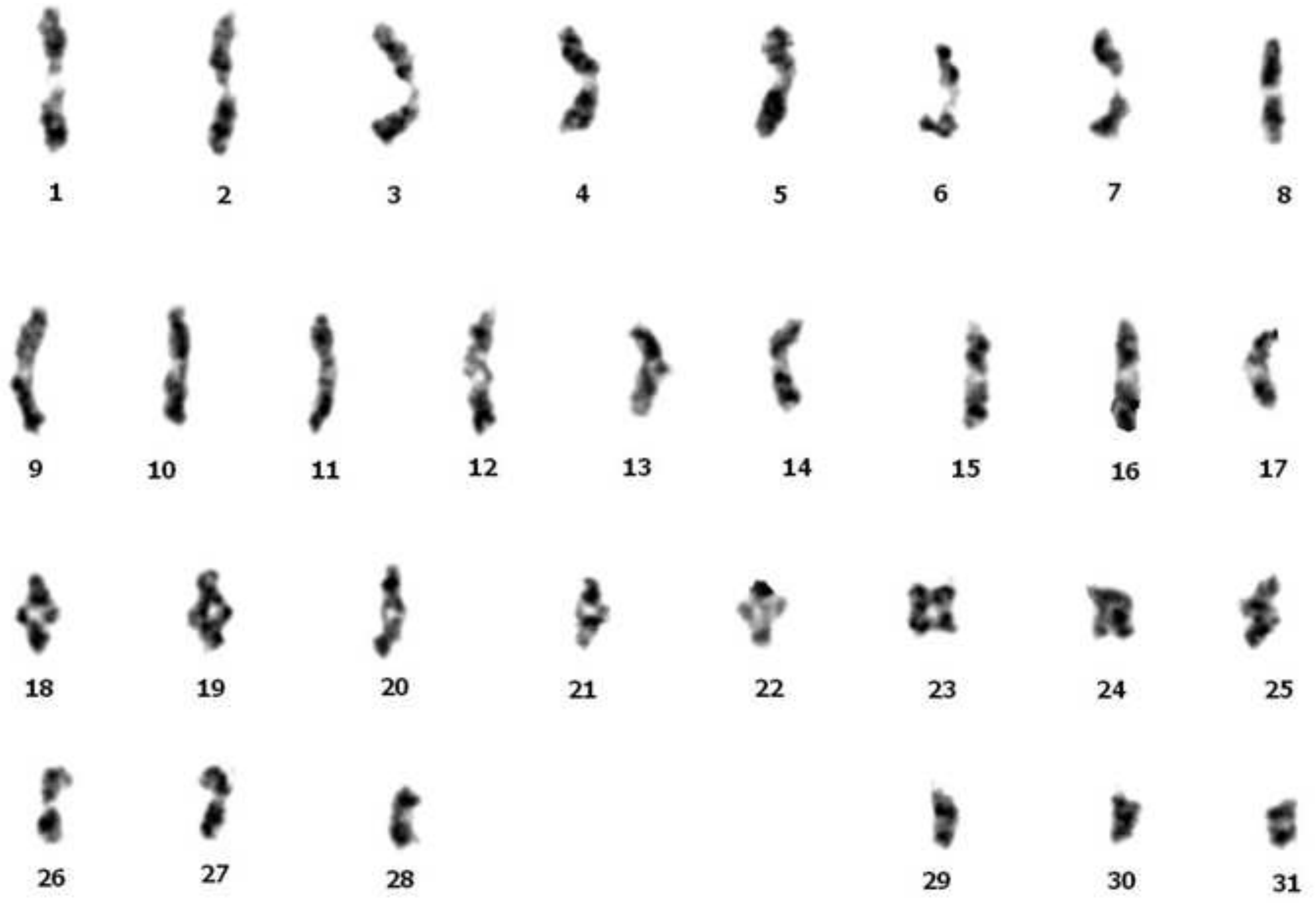

Supplementary Information 1

Click here to access/download
Supplementary Material
Supplementary_Information.docx

Supplementary Information 2

Click here to access/download
Supplementary Material
Supplementrary_information2.xlsx

Response to Reviewer 1

Click here to access/download
Supplementary Material
Response_to_Reviewer_1_ENG.docx

Click here to access/download
Supplementary Material
Response_to_Reviewer_2_ENG.docx

Click here to download Personal Cover Cover_letter_ENG.docx

Sep 17, 2017

Dear Editor of *Gigascience*,

Thank you for sending our manuscript out for review and for obtaining constructive feedback from two expert referees. We appreciate the careful reading of our manuscript by the reviewers. We would also like to thank you for allowing us to submit a fully revised version that addresses all points of the reviewers and the academic editor.

We appreciate suggestions of all the reviewers on our manuscript; all the comments made by the reviewers were quite valid. We have responded to all comments point-by-point in the rebuttal.

Followed the reviewer's suggestion, we tried our best to conduct manual curation for all predicted genes (>20,000). We also changed all related analysis results in the manuscript and added one co-author who helped with this revision.

We hope that our revised manuscript, strengthened by reviewer comments, will meet the high-quality standard of *Gigascience*. We are always ready to strengthen our manuscript once again following the comments of the editors and reviewers at the next revision if necessary.

Looking forward to hearing from you again.

Thank you.

With best regards,

Prof. Seung-Won Park
Department of Biotechnology,
Catholic University of Daegu, Gyeongsan-si, Gyeongsangbuk-do 38430, Republic of Korea,
Tel: +82-53-850-3176, E-mail: microsw@cu.ac.kr
