# Supplementary material for: Genome sequence of the Japanese oak silk moth, Antheraea yamamai: the first draft genome in the family Saturniidae
Source: Gigascience. 2017 Nov 27;7(1):gix113. doi: 10.1093/gigascience/gix113 (PMC5774507; doi:10.1093/gigascience/gix113)

Figure S1. Top 20 terms in each 7 Interproscan5 analysis. (CDD, Pfam, PRINTS, ProSitePatterns, ProSiteProfiles, TIGRF, Gene Ontology)


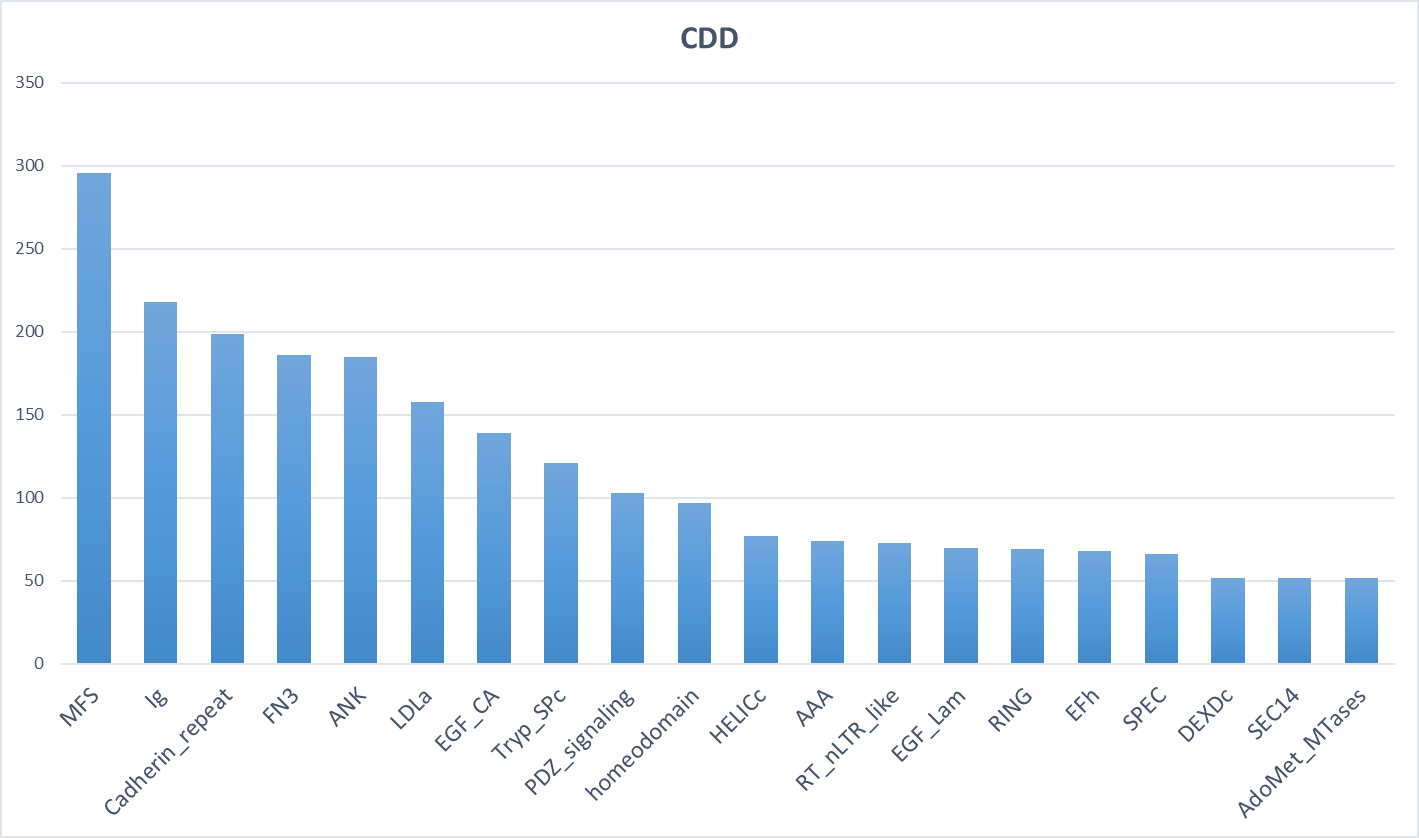


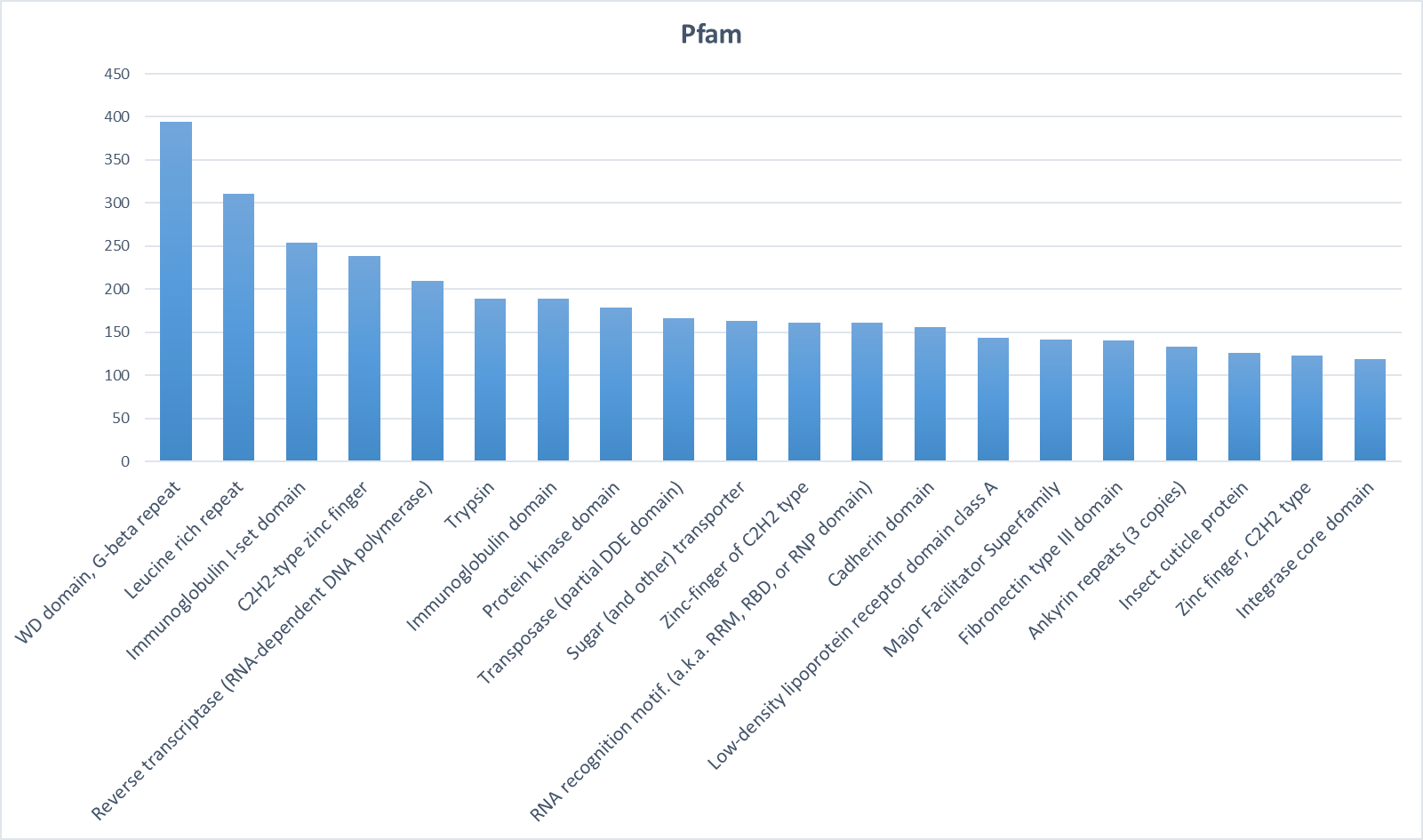


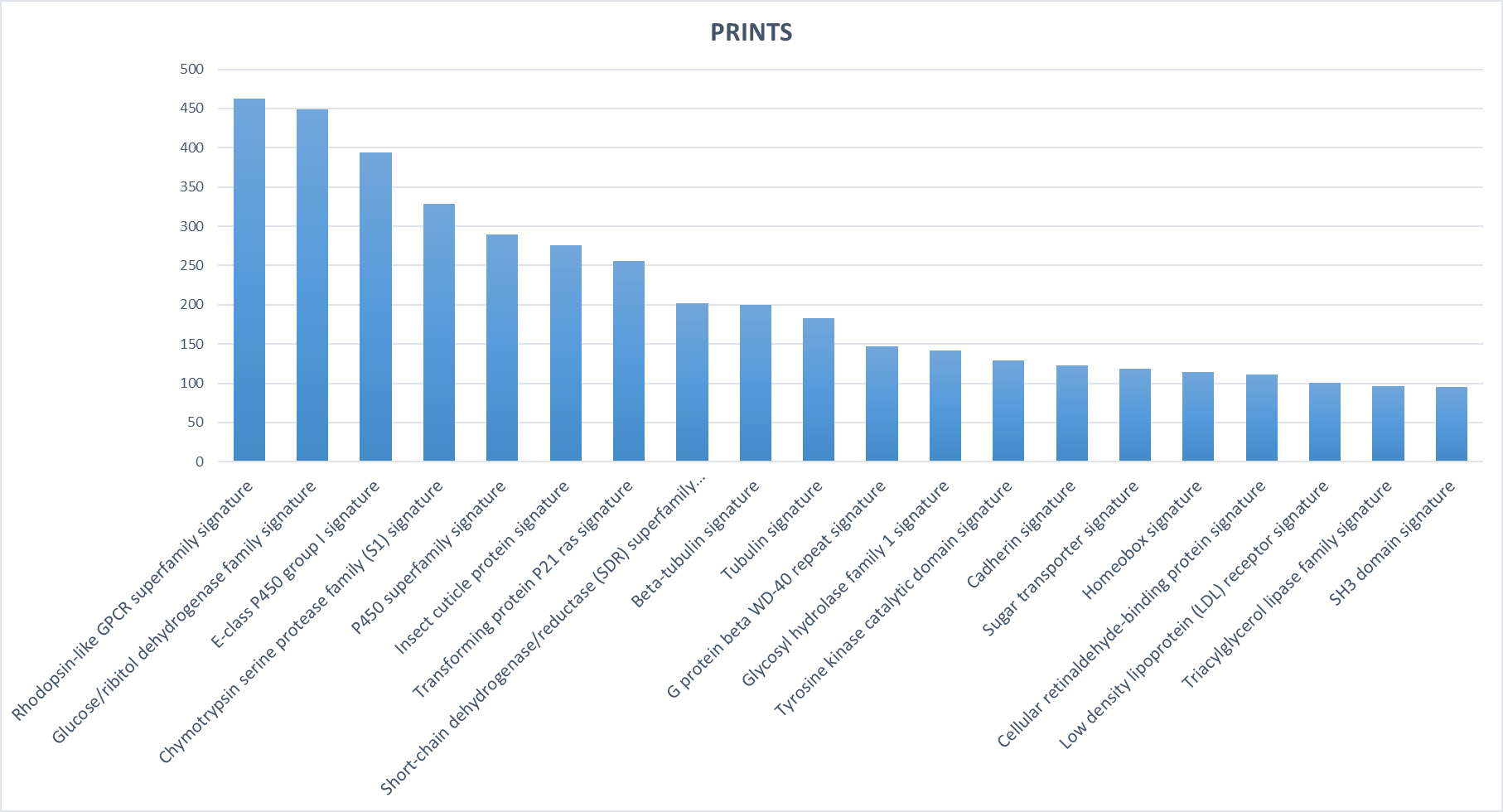


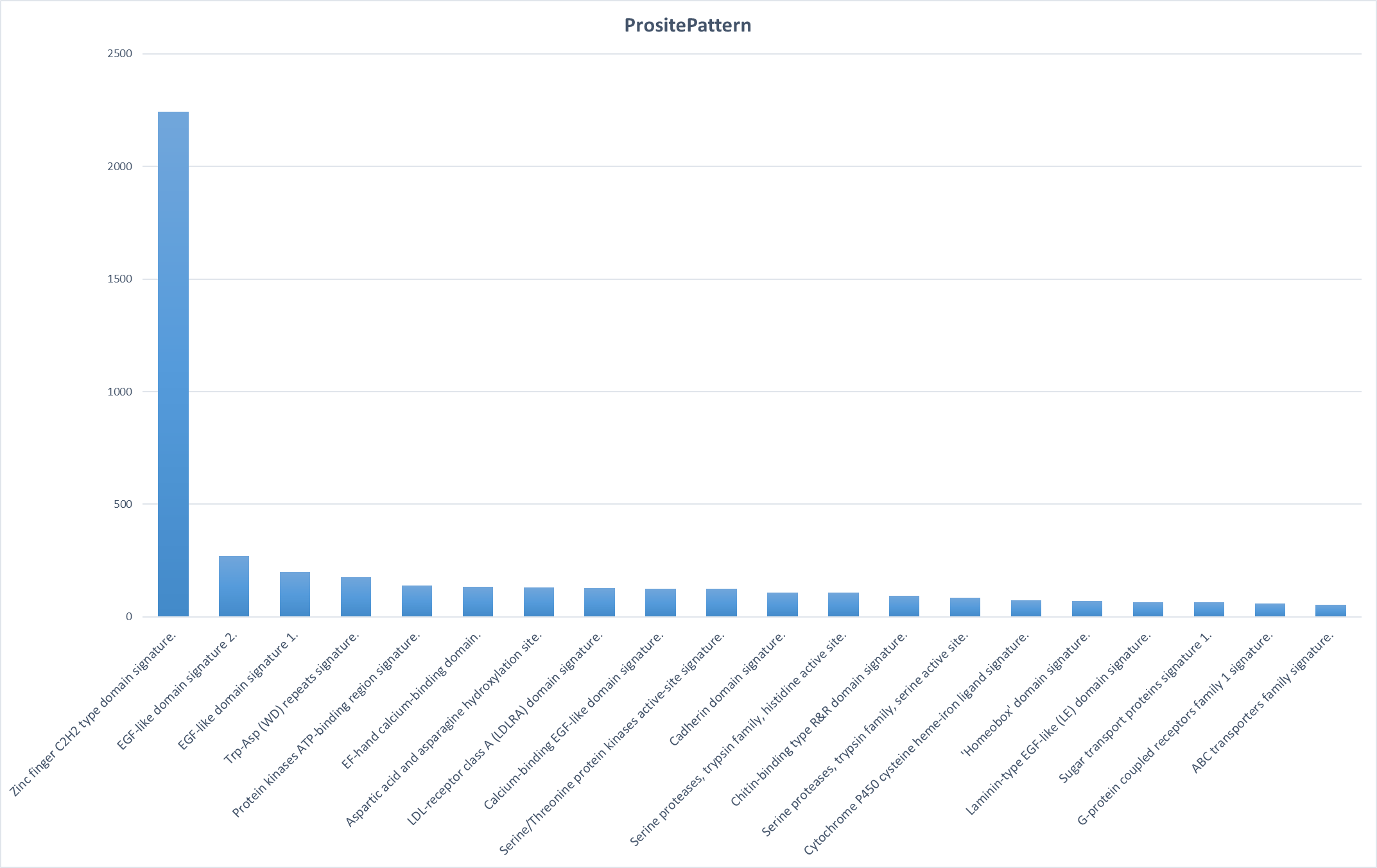


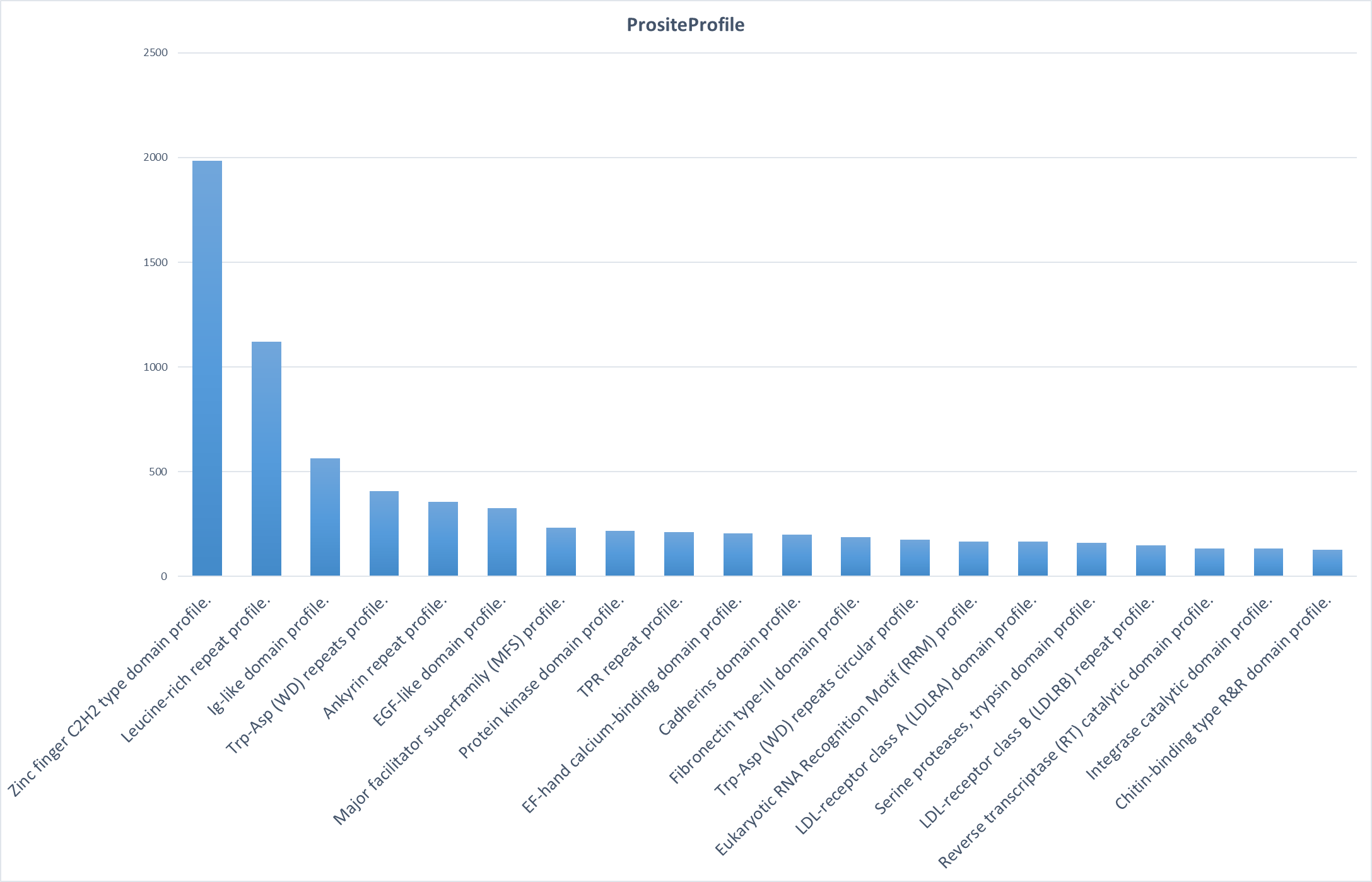


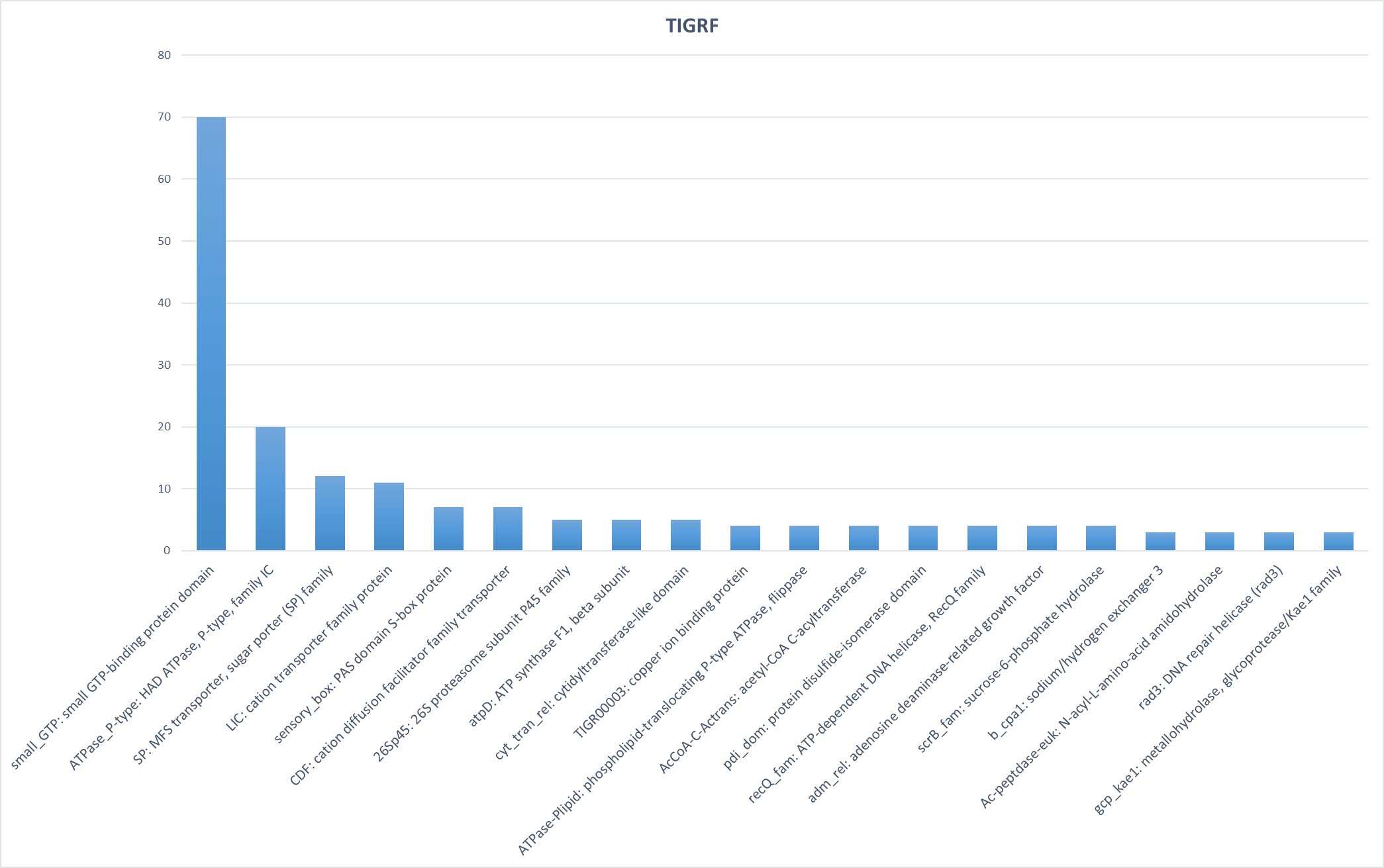


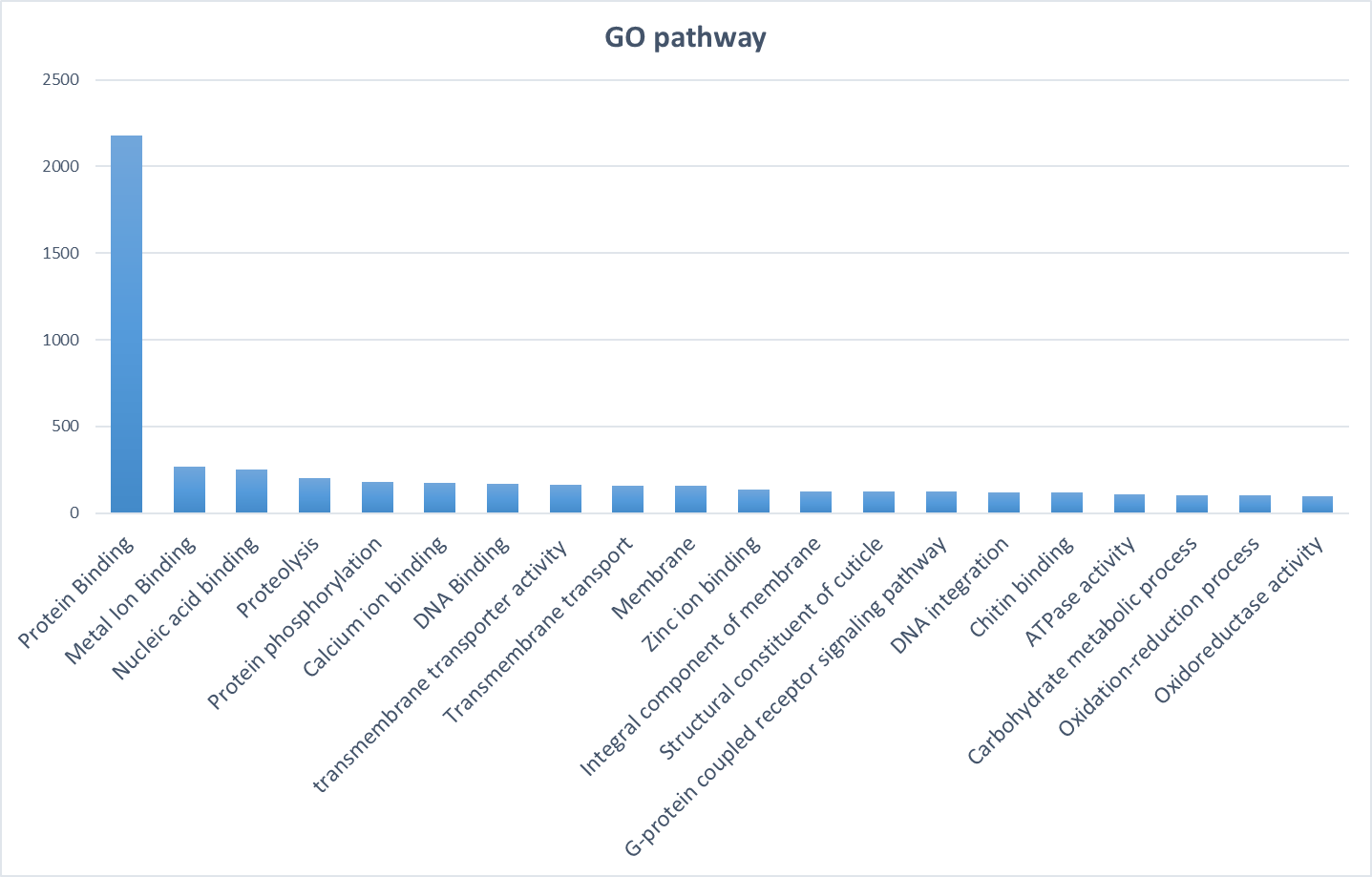

Supplement: Additional files [file gix113_supp.zip › Supplementary_Information.docx]
