# Supplementary material for: Genome sequence of the Japanese oak silk moth, Antheraea yamamai: the first draft genome in the family Saturniidae
Source: Gigascience. 2017 Nov 27;7(1):gix113. doi: 10.1093/gigascience/gix113 (PMC5774507; doi:10.1093/gigascience/gix113)
Supplement: Response-to-Reviewer-Comments_Original-Submission.pdf [file gix113_response-to-reviewer-comments_original-submission.pdf]

Author's Response To Reviewer Comments

Dear Editor,

We corrected our manuscript followed the editor's comment.

The response and details of revision can be found in memo section.

We hope our revised manuscript can meet the quality standard of the editor and future peer reviewers.

If you need anything, just let us know.

On behalf of all authors,
Seung-Won Park
