# Supplementary material for: Genome sequence of the Japanese oak silk moth, Antheraea yamamai: the first draft genome in the family Saturniidae
Source: Gigascience. 2017 Nov 27;7(1):gix113. doi: 10.1093/gigascience/gix113 (PMC5774507; doi:10.1093/gigascience/gix113)
Supplement: Response-to-Reviewer-Comments_Revision-1.pdf [file gix113_response-to-reviewer-comments_revision-1.pdf]

Author's Response To Reviewer Comments

We appreciate suggestions of all the reviewers on our manuscript; all the comments made by the reviewers were quite valid. We have responded to all comments point-by-point in the seperate rebuttal. We hope our revised manuscript can meet the quality standard of reviewers.
