# Supplementary material for: Genome sequence of the Japanese oak silk moth, Antheraea yamamai: the first draft genome in the family Saturniidae
Source: Gigascience. 2017 Nov 27;7(1):gix113. doi: 10.1093/gigascience/gix113 (PMC5774507; doi:10.1093/gigascience/gix113)
Supplement: Reviewer-1-Report-(Revision-1) -- Reuben William Nowell — 09 Jun 2017 Reviewed [file gix113_reviewer-1-report-(revision-1).pdf]

Reviewer Report

Title: Genome sequence of Japanese oak silk moth, Antheraea yamamai: the first draft genome in family Saturniidae

Version: Revision 1 **Date:** 6/9/2017

Reviewer name: Reuben William Nowell

Reviewer Comments to Author:

Overall commentsThe manuscript of Kim et al. describes the genome sequencing, assembly and annotation of the Japanese oak silk moth, Antheraea yamamai, using a range of both short- and long-read technologies. The data appear to be of high quality and coverage, and the methods employed during sequencing, assembly and annotation are clear and concise, resulting in a large genome but with high overall quality scores. My only major comments regard the potential effects of both heterozygosity and contamination on their final results, and I would like to see some evidence that these issues, which may be minor, have at least been considered by the authors prior to the release of their genome. Once these issues have been addressed, I agree that this genome will be a useful resource for future studies, particularly as a comparison to the related Bombyx mori, and therefore will be suitable for publication in GigaScience.Major commentsComment #1. Heterozygosity of these data is mentioned a number of times, including in Fig. 3 which shows a bimodal kmer distribution and is a strong indication of heterozygosity. However I cannot see in the manuscript how or indeed if this issue was addressed during the assembly procedure. Therefore it is possible that heterozygous regions have been coassembled and are present in the assembly. I wonder if this may in part explain the large genome span for this species (~180 Mb longer than B. mori), and, perhaps, the detected gene-family expansions. I feel that more should be done to convince me that these are not artefacts of coassembled heterozygous regions present in the assembly. For example, the authors may wish to perform a trial assembly using the 'heterozygous aware' assembler Platanus [1], or attempt to remove highly similar scaffolds from the assembly using software such as Redundans [2]. These approaches may or may not lead to substantial differences in final assembly, however I think the work would benefit greatly from some further investigations on the matter.Comment #2. Similarly, I do not see evidence of any investigations into the extent to which the data may be contaminated with the DNA of non-target organisms, or how/if potential contaminants were detected and removed prior to final assembly. I note the removal of the guts from samples prior to DNA extraction, which is a good idea, but contamination from non-target organisms such as bacteria, fungi etc is very common (usually occurring during sample preparation from organisms present on the surface of the animal) and can also result in bimodal kmer distributions as observed here. I suspect the bimodality of these data is indeed due to heterozygosity, as inferred by the authors, but it would be wise to check. A number of tools exist for this purpose, e.g., Blobtools [3]; this tool is also useful for visualising coverage distributions and therefore detecting heterozygosity. Again, I think the work would benefit from some indication that the data have been scrutinized for potential contaminants prior to assembly.Minor commentsLine 14: Genome span is quoted as 656 Mb, but is this only including scaffolds of 2 kb and greater (as indicated in the

parentheses)? I find this cut-off threshold rather large - it is common to discard short sequences of perhaps less than 200 - 500 bp in length, but 2 kb seems excessive. I suspect that using a lower length cutoff will result in a substantial increase the span and decrease the N50. I also suspect that many of these short scaffolds may be cleaned up as part of my other suggestions in this review (eg #1), but I would urge caution in employing unusually high thresholds as these may favourably bias assembly statistics.Line 30: Missing word: "largest family in the Lepidoptera"Line 38: Suggested change: "is their silk" to "is the silk"Line 83: Suggested rephrasing: "In the 19-mer distribution, there was a second peak in the half x-axis of the main peak which indicates heterozygosity." to "In the 19-mer distribution, a second peak at approximately half the coverage value (x-axis) of the main peak indicates heterozygosity." or something similar.Line 87: See major comment #1.Line 103: Typo: "previous study" to "previous studies" as multiple references are cited.Line 109: The authors may with to try further scaffolding with their assembled transcriptomes using tools such as SCUBAT [4] and L_RNA_Scaffolder [5], although their assembly stats are already excellent.Line 126: What exactly does CENSOR do?Line 127: Again, a very brief explanation of the 'no_is' option would be useful here.Line 129: Typo: "genome was LINE element" to "were LINE elements"Line 144: "This indicates that there are differences in the genome evolution process… " - I find this statement vague and unsatisfactory. What are these "genome evolution process[es]" the authors allude to? I suggest the authors are more explicit in how they are interpreting their results.Line 165: "To identify the function of predicted genes, Swiss-Prot[47], Uniref100[47], NCBI NR[48] database, and gene information of B. mori and D. melanogaster was employed for sequence similarity search using blastp." - the sentence is clunky and could be rephrased.Line 168: Typo: "protein domain search was" to "protein domain searches were"Line 189: The authors should state clearly on which data the OrthoMCL clustering analysis was performed. I assume it is the same set of taxa as in the phylogeny, but this is not made explicit.Line 205: Typo: "(D.plexippus, M.cinxia and H. meplmene)" to "(D. plexippus, M. cinxia and H. melpomene)" [also note missing spaces in taxa names]Line 213: Typo: "expended" to "expanded"Line 215: Missing word: "specific" to "lineage-specific" to make the point clearerLine 229: Typo: "cocoon is serves" to "cocoon serves"Line 239: Typo: "unlike B. mori who feed" to "which feed"Line 249: Again, some details regarding what is meant by "genome evolution processes" would be goodLine 255: Grammar: "And constructed … " to "In addition, constructed … "Refs: Reference #7 contains Chinese charactersTable 1: I suggest inclusion of another column in the data tables showing the proportion of reads / bases retained after trimming / filtering as a useful measure of the overall quality of the data.Table 5: "Average scaffold length" should be N50 scaffold length I thinkTable 8: Does "genome coverage" value have units of % or Mb?Fig 5: Typo: "node value" to "node values"References1. Kajitani R, Toshimoto K, Noguchi H, Toyoda A, Ogura Y, Okuno M, et al. Efficient de novo assembly of highly heterozygous genomes from whole-genome shotgun short reads. Genome Res. 2014;24: 1384-1395.2. Pryszcz LP, Gabaldón T. Redundans: an assembly pipeline for highly heterozygous genomes. Nucleic Acids Res. 2016;44: e113.3. Laetsch DR, Koutsovoulos G, Stajich J, Kumar S. Drl/Blobtools: Blobtools V0.9.19.5 [Internet]. Zenodo; 2016. doi:10.5281/zenodo.1776994. Koutsovoulos G. SCUBAT [Internet]. Available: https://github.com/GDKO/SCUBAT25. Xue W, Li J-T, Zhu Y-P, Hou G-Y, Kong X-F, Kuang Y-Y, et al. L_RNA_scaffolder: scaffolding genomes with transcripts. BMC Genomics. 2013;14: 604.

Level of Interest

Please indicate how interesting you found the manuscript: An article whose findings are important to those with closely related research interests

Quality of Written English

Please indicate the quality of language in the manuscript: Acceptable

Declaration of Competing Interests

Please complete a declaration of competing interests, considering the following questions:

- Have you in the past five years received reimbursements, fees, funding, or salary from an organisation that may in any way gain or lose financially from the publication of this manuscript, either now or in the future?
- Do you hold any stocks or shares in an organisation that may in any way gain or lose financially from the publication of this manuscript, either now or in the future?
- Do you hold or are you currently applying for any patents relating to the content of the manuscript?
- Have you received reimbursements, fees, funding, or salary from an organization that holds or has applied for patents relating to the content of the manuscript?
- Do you have any other financial competing interests?
- Do you have any non-financial competing interests in relation to this paper?

If you can answer no to all of the above, write 'I declare that I have no competing interests' below. If your reply is yes to any, please give details below.

I declare that I have no competing interests

I agree to the open peer review policy of the journal. I understand that my name will be included on my report to the authors and, if the manuscript is accepted for publication, my named report including any attachments I upload will be posted on the website along with the authors' responses. I agree for my report to be made available under an Open Access Creative Commons CC-BY license (http://creativecommons.org/licenses/by/4.0/). I understand that any comments which I do not wish to be included in my named report can be included as confidential comments to the editors, which will not be published.

I agree to the open peer review policy of the journal
