# Supplementary material for: Genome sequence of the Japanese oak silk moth, Antheraea yamamai: the first draft genome in the family Saturniidae
Source: Gigascience. 2017 Nov 27;7(1):gix113. doi: 10.1093/gigascience/gix113 (PMC5774507; doi:10.1093/gigascience/gix113)
Supplement: Reviewer-2-Report-(Revision-1) -- Simon Baxter — 17 Jun 2017 Reviewed [file gix113_reviewer-2-report-(revision-1).pdf]

Reviewer Report

Title: Genome sequence of Japanese oak silk moth, Antheraea yamamai: the first draft genome in family Saturniidae

Version: Revision 1 **Date:** 6/17/2017

Reviewer name: Simon Baxter

Reviewer Comments to Author:

Kim et al. use Illumina and PacBio sequencing generate an assembly of A. yamamai, plus illumina transcriptomes of 10 tissues in triplicate. This species spins desirable silk with commercial interest. This manuscript is largely a presentation of new genomic resources, which will no doubt be analysed in detail in the future. I recommend submitting the genomic data to LepBase, which will enable the community to maximise the use of the dataset and increase citations for this work. Sequencing and assembling insect genomes into large scaffolds can be extremely difficult, so I congratulate the authors on achieving such good assembly statistics.Major concerns1. The number of predicted genes is pretty high compared to other Leipdoptera. It would be worth speculating why this is the case. As automated gene predictions often contain errors, it's becoming more common for research labs and communities to manually check and improve annotations on a subset of predicted genes. This doesn't appear to have been done here, but the authors should consider doing so, using programs such as Apollo.2. The biological justification for this genome project was to learn more about A. yamamai silk production. It would have been nice to see some comparative analysis of silk gland transcriptomes with Bombyx mori. Although transcriptome libraries were sequenced in triplicate, it wasn't clear whether variation in gene expression was analysed for this data, or whether this work represents a resource for future work. It appears the transcriptomes were simply pooled and used for genome annotation.3. It's not clear how the karyotyping links in with the geonome sequencing, as scaffolds don't appear to be assigned to chromosomes. Perhaps this section could be moved to the end of the paper, along with the future research goal of assigning scaffolds to one of the 31 chromosomes.4. The individual sequence is claimed to be an inbred male, yet the PSMC is applied to generate insight into the past effective population size. This is entirely inappropriate, as genetic bottlenecks from a single individual may severely bias the result. This should be removed from the study, or multiple wild individuals sequenced and analysed using this approach.Minor commentsAbstract: It would be useful to say what the 'drastic' difference is between tensan silk and common silk.I used the timetree.org function Estimated Time to check the divergence between Antheraea yamamai and Bombyx mori. It came up with 84 MYA, rather than 87 MYA as presented in the manuscript. Perhaps there were some differences in the settings used, but it would be worth checking this and including any specific settings applied.Line 37. I couldn't locate a reference for 0.0041 MYA split between Bombyx mori and Mandariana. Perhaps you could clarify this, or remove it, if it's not necessary. As Bombyx mori and mandariana shared the most common recent ancestor, it's difficult to follow the logic behind the claim that mandarina is 'evolutionarily further away' from A. yamamai than mori. This section needs more support.Line 37. 'a' rather than 'as'.Line 38. The section beginning "The most unique species-specific phenotypic trait of A. yamamai…" Could be modified to

something like "A. yamamai produces tensan silk[5] which shows distinctive characteristics compared to common silk from Bombyx mori[6-8], such as thickness, bulkiness, compressive elasticity and resistance to [specific?] chemicals."Line 42. The reference to 'peptides' is a bit ambiguous. Do you mean anti-microbial peptides?Line 44. Antheraea yamamai has 43822 nucleotide sequences available on NCBI, so claiming there is no genomic information isn't strictly true.Line 46. You could include "present the annotated genome sequence…" or "annotated with transcriptome datasets form 10 different tissues"Line 47. "Gene expression" could be replaced with transcriptomeLine 51. It may be appropriate to remind readers a male was sequenced as they are the homogametic sex in lepidopterans and this avoids sequencing excessive repeats on the W chromosome.Lines 57-58. Delete 'transcriptomic library construction' and re-write this sentence. You say the gut was removed before genome and transcriptome sequencing, but then sequenced the midgut transcriptome.Line 68. Provide complete names for tissue types and specific developmental stages. You may mean integument rather than 'skin'?Line 85. Was inbreeding accomplished using single pair sib-matings for 10 generations? Or did you just rear the colony for 10 generations. Please specify.Line 87. Diamondback moth, rather than 'black diamond moth' (the diamonds are typically very pale)Line 89. To predict genome size, flow cytometry of stained nuclei is often used. Predicting a 709 Mb genome from a polymorphic dataset seems a little risky as heterozygous haplotypes presumably increase genome size. This caveat should be included.144-145. Using fast evolving repetitive elements to claim "there are differences in the genome evolution process between Saturniidae and Bombycidae families" isn't very surprising, or useful, as they diverged about 84 million years ago.239-244. It would be appropriate to include a figure or more detail of the flavonoid data, as the claims are not supported with clear data.The manuscript needs further language review, perhaps with some help from the editors.

Level of Interest

Please indicate how interesting you found the manuscript: An article of importance in its field

Quality of Written English

Please indicate the quality of language in the manuscript: Needs some language corrections before being published

Declaration of Competing Interests

Please complete a declaration of competing interests, considering the following questions:

- Have you in the past five years received reimbursements, fees, funding, or salary from an organisation that may in any way gain or lose financially from the publication of this manuscript, either now or in the future?
- Do you hold any stocks or shares in an organisation that may in any way gain or lose financially from the publication of this manuscript, either now or in the future?

- Do you hold or are you currently applying for any patents relating to the content of the manuscript?
- Have you received reimbursements, fees, funding, or salary from an organization that holds or has applied for patents relating to the content of the manuscript?
- Do you have any other financial competing interests?
- Do you have any non-financial competing interests in relation to this paper?

If you can answer no to all of the above, write 'I declare that I have no competing interests' below. If your reply is yes to any, please give details below.

I declare that I have no competing interests

I agree to the open peer review policy of the journal. I understand that my name will be included on my report to the authors and, if the manuscript is accepted for publication, my named report including any attachments I upload will be posted on the website along with the authors' responses. I agree for my report to be made available under an Open Access Creative Commons CC-BY license (http://creativecommons.org/licenses/by/4.0/). I understand that any comments which I do not wish to be included in my named report can be included as confidential comments to the editors, which will not be published.

I agree to the open peer review policy of the journal
